# The snoRNA-like lncRNA LNC-SNO49AB drives leukemia by activating the RNA-editing enzyme ADAR1

**DOI:** 10.1038/s41421-022-00460-9

**Published:** 2022-11-01

**Authors:** Wei Huang, Yu-Meng Sun, Qi Pan, Ke Fang, Xiao-Tong Chen, Zhan-Cheng Zeng, Tian-Qi Chen, Shun-Xin Zhu, Li-Bin Huang, Xue-Qun Luo, Wen-Tao Wang, Yue-Qin Chen

**Affiliations:** 1 Guangdong Province Key Laboratory of Pharmaceutical Functional Genes, MOE Key Laboratory of Gene Function and Regulation, State Key Laboratory for Biocontrol, School of Life Sciences, Sun Yat-sen University, Guangzhou, Guangdong China; 2 The First Affiliated Hospital of Sun Yat-sen University, Guangzhou, Guangdong China

**Keywords:** Non-coding RNAs, Leukaemia

## Abstract

Long noncoding RNAs (lncRNAs) are usually 5′ capped and 3′ polyadenylated, similar to most typical mRNAs. However, recent studies revealed a type of snoRNA-related lncRNA with unique structures, leading to questions on how they are processed and how they work. Here, we identify a novel snoRNA-related lncRNA named LNC-SNO49AB containing two C/D box snoRNA sequences, SNORD49A and SNORD49B; and show that LNC-SNO49AB represents an unreported type of lncRNA with a 5′-end m7G and a 3′-end snoRNA structure. LNC-SNO49AB was found highly expressed in leukemia patient samples, and silencing LNC-SNO49AB dramatically suppressed leukemia progression in vitro and in vivo. Subcellular location indicated that the LNC-SNO49AB is mainly located in nucleolus and interacted with the nucleolar protein fibrillarin. However, we found that LNC-SNO49AB does not play a role in 2′-O-methylation regulation, a classical function of snoRNA; instead, its snoRNA structure affected the lncRNA stability. We further demonstrated that LNC-SNO49AB could directly bind to the adenosine deaminase acting on RNA 1(ADAR1) and promoted its homodimerization followed by a high RNA A-to-I editing activity. Transcriptome profiling shows that LNC-SNO49AB and ADAR1 knockdown respectively share very similar patterns of RNA modification change in downstream signaling pathways, especially in cell cycle pathways. These findings suggest a previously unknown class of snoRNA-related lncRNAs, which function via a manner in nucleolus independently on snoRNA-guide rRNA modification. This is the first report that a lncRNA regulates genome-wide RNA A-to-I editing by enhancing ADAR1 dimerization to facilitate hematopoietic malignancy, suggesting that LNC-SNO49AB may be a novel target in therapy directed to leukemia.

## Introduction

Long noncoding RNAs (lncRNAs) constitute a class of noncoding transcripts longer than 200 nucleotides with no apparent protein-coding potential. They are emerging as potential key regulators in numerous biological processes across every branch of life, exhibiting a surprising range of shapes and sizes^1–4^. Remarkably, recent studies have reported that the unusual biogenic processes of small nucleolar RNAs (snoRNAs) generate a group of lncRNAs, named snoRNA-related lncRNAs^5,6^, adding a novel layer to lncRNA biogenesis. SnoRNAs are evolutionarily conserved ncRNAs which are 60–300 nt in length and function mainly as guide RNAs during site-specific rRNA modification^7–9^. They are mostly processed from introns of protein-coding or noncoding genes called “host genes”^10,11^. Two types of snoRNA-related lncRNAs, sno-lncRNAs with a snoRNA at both ends and SPAs (5′ snoRNA capped and 3′ polyadenylated lncRNAs), have been identified^5,12,13^. The high abundance of these lncRNAs indicates that they are unlikely to be precursors of snoRNAs, suggesting previously unrecognized regulatory potential of snoRNA sequences.

To date, a set of putative snoRNA-related lncRNAs have been identified in human cells^6,12^, with only a small fraction characterized. For example, sno-lncRNA1-5^5^ and SPAs^12^, encoded in the deletion chromosome region of Prader-Willi syndrome (PWS), a neurodevelopmental genetic disorder^14^, sequester multiple RNA-binding proteins away from their normal functional sites to affect mRNA metabolism and contribute to PWS pathogenesis. A sno-lncRNA named SLERT was reported to control rRNA synthesis by directly binding to the DEAD-box RNA helicase DDX21 via a 143-nt non-snoRNA sequence to alter the conformation of DDX21 in cancer^15^. SnoRD86-cSPA is speculated to operate as a decoy for snoRNP core proteins, sequestering them away from the nuclear^16^. These studies suggested the potential functional diversity of these ncRNAs. Given their important roles in cellular processes, additional snoRNA-related lncRNAs will be of great interest to be characterized and investigated for their roles in the disease context.

Leukemia is the most prevalent and aggressive blood cancer and can be subdivided into different subtypes according to cell maturity (acute or chronic) and cell type (lymphocytic or myeloid)^17,18^. With standard chemotherapies, the event-free survival of acute lymphocytic leukemia (ALL) is ~60%–80%, and only 35%–40% of younger (aged < 60 years)^19^ and 5%–15% of older (aged > 60 years) patients with acute myeloid leukemia (AML) survive more than 5 years^20^. Thus, there is an ever-present need to better understand the genetic and molecular mechanisms of leukemia biogenesis and progression. Recent advances in understanding the noncoding RNA (ncRNA) transcriptome have highlighted the importance of various ncRNA species in leukemia^21–23^. Interestingly, functional validation studies have indicated that snoRNAs are required for leukemogenesis^9,24–26^, which has challenged the view that snoRNAs merely function as housekeeping genes for the posttranscriptional modification of rRNAs. Among these snoRNAs, two snoRNAs, SNORD49A and SNORD49B, attracted our attention. Although they are classical C/D box snoRNAs, both function independent of rRNA 2′-O-methylation^24^, suggesting alternative mechanisms by which they play oncogenic roles in leukemia. Interestingly, they were predicted to generate a snoRNA-related lncRNA^6^. Thus, we questioned whether SNORD49A/B can also function as snoRNA-related lncRNAs and contribute to hematopoietic malignancy.

In this study, we identified a novel and highly expressed snoRNA-related lncRNA, named as LNC-SNO49AB, in leukemia patient samples. Sequence and structure analyses showed that LNC-SNO49AB contains two C/D box snoRNA sequences, SNORD49A and SNORD49B, and a 5′-end m7G and a 3′-end SNORD49A, which is a new type of snoRNA-related lncRNA. We further showed that LNC-SNO49AB directly binds to adenosine deaminase acting on RNA 1 (ADAR1) and promotes its dimerization and function to elevate the global rate of RNA A-to-I editing, thereby regulating hematopoietic malignancy. Our study reveals a pivotal role for a snoRNA-related lncRNA with a previously unknown structure, LNC-SNO49AB, in ADAR-mediated RNA editing and leukemia progression.

## Results

### Characterization of LNC-SNO49AB in leukemia

**Fig. 1:**
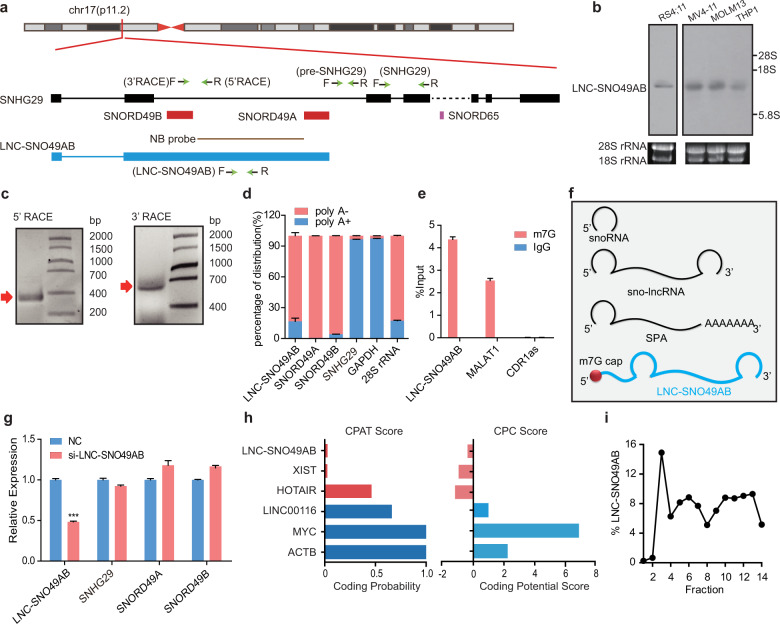
Characterization of LNC-SNO49AB in leukemia. **a** The schematic of SNORD49AB gene locus. Top: chr17(p11.2) locus; Middle: the schematic represents SNHG29, SNORD65 and SNORD49A/B. Bottom: Physical map depicts LNC-SNO49AB gene locus. The qRT-PCR and RACE primer were designed as indicated (green arrow). The probe used for northern blotting (NB) is also shown. **b** Northern blot analysis of LNC-SNO49AB expression in total RNA obtained from four leukemia cell lines. Representative results are shown from three replicates. **c** Agarose gel electrophoresis analysis of 5′ (right) and 3′ RACE (left) for cDNA generated from RS4;11 cells. Representative results are shown from three replicates. **d** Poly(A)+ and poly(A)– RNAs were extracted from RS4;11 cells and subjected to RT-qPCR analysis. LNC-SNO49AB, SNORD49A and SNORD49B were enriched in the poly(A)– fraction. SNHG29 is polyadenylated. GAPDH and 28 S rRNA were used as controls for poly(A)+ and poly(A)– RNAs, respectively. Error bars represent SEM in triplicate experiments. **e** RNA immunoprecipitation (RIP) using an anti-m7G cap antibody and IgG followed by RT-qPCR analysis showed that LNC-SNO49AB is capped by m7G. MALAT1 and circular RNA CDR1as were used as the controls for m7G capped positive and negative RNA, respectively. Error bars represent SEM in triplicate experiments. **f** Schematic representation of snoRNAs and two known kinds of snoRNA-related lncRNAs, including sno-lncRNA and SPA. LNC-SNO49AB represents a novel kind of snoRNA-related lncRNA with a m7G cap and snoRNA end. **g** Expression levels of LNC-SNO49AB, SNHG29 and SNORD49AB after knockdown of LNC-SNO49AB by siRNAs. Data were normalized to *GAPDH* mRNA. *P < 0.05, **P < 0.01, and ***P < 0.001 by Student’s *t*-test. Error bars represent SEM of the means. **h** Scores of LNC-SNO49AB and other known coding and noncoding RNAs as determined by analysis with CPAT (http://lilab.research.bcm.edu/) and CPC (http://cpc.cbi.pku.edu.cn). **i** Sucrose sedimentation analysis of RNA levels from each fraction of MOLM13 cells as measured by RT-qPCR. Relative levels of *LNC-SNO49AB*, *ACTB* (mRNA) and *HY1* (noncoding RNA) are shown for each collected fraction.

To determine whether SNORD49A/B can function as a snoRNA-related lncRNA, we first validated the existence of these lncRNAs by northern blotting and rapid amplification of cDNA ends (RACE). As shown in Fig. 1a, SNORD49A and SNORD49B are both located in the second intron of the noncoding gene SNHG29 on p11.2 of chromosome 17. Northern blot analysis with a probe for detecting the sequence between SNORD49A and SNORD49B confirmed the existence of only one mature transcript in four leukemic cell lines (Fig. 1b). We next performed 5′ and 3′ RACE and then sequenced to conform the length of the transcript. Sequence analysis revealed that the transcript contains the first exon of *SNHG29* and the 5′ end of second exon to the 3′ end of SNORD49A of *SNHG29* and is 804 nt (Fig. 1c and Supplementary Fig. S1a, b). We also overexpressed LNC-SNO49AB into 293T cells to further support the length of mature LNC-SNO49AB (Supplementary Fig. S1c, d). These results suggest that this gene locus forms a snoRNA-related lncRNA that has SNORD49A/B sequences, thus, we named this lncRNA LNC-SNO49AB (Fig. 1a). We then characterized the structure of LNC-SNO49AB. LNC-SNO49AB lacks the poly(A) tail (Fig. 1d) and is terminated by SNORD49A at the 3′-end (Fig. 1a). Unexpectedly, we found that SNORD49B is in the middle of LNC-SNO49AB (Fig. 1a), not at the 5′-end as those previous reports^5,12^, showing that LNC-SNO49AB has a unique structure. We further showed that LNC-SNO49AB can be precipitated by anti-m7G antibody (Fig. 1e), indicating that it is capped by m7G, similar to most mRNAs and lncRNAs, not m3G or any other modification of classical eukaryotic snoRNA^27,28^. We name the lncRNAs “lnc-snoRNAs”, with a 5′-end m7G and a 3′-end snoRNA structure (Fig. 1f).

Next, we characterized the features of LNC-SNO49AB. The relative expression level of LNC-SNO49AB is lower than SNHG29 but higher than the precursor pre-SNHG29 (Supplementary Fig. S1e). The addition of siRNAs that targeted the non-snoRNA regions of LNC-SNO49AB did not alter the expression of SNORD49A or SNORD49B (Fig. 1g), suggesting that LNC-SNO49AB is a stable lncRNA, not a snoRNA precursor. As LNC-SNO49AB was terminated by a snoRNA at the 3′ end, we sought to determine whether the 3′-end snoRNA can provide lncRNAs with stability comparable to that of an RNA with a poly(A) tail. We examined the RNA stability with Actinomycin D treatment and results showed that the half-life of LNC-SNO49AB is similar to that of *GAPDH* mRNA and lncRNAs, which have poly(A) tails (Supplementary Fig. S1f). In addition, the DNA sequence of LNC-SNO49AB is relatively conserved among primates but not in other mammals; SNORD49A and SNORD49B are also conserved in mice (Supplementary Fig. S1g). Finally, bioinformatics and polysome profiling analysis suggested that LNC-SNO49AB has no coding potential (Fig. 1h, i and Supplementary Fig. S1h). In brief, LNC-SNO49AB represents an unreported type of lncRNA with a 5′-end m7G and a 3′-end snoRNA structure.

### LNC-SNO49AB processing requires intact site-specific snoRNA

**Fig. 2:**
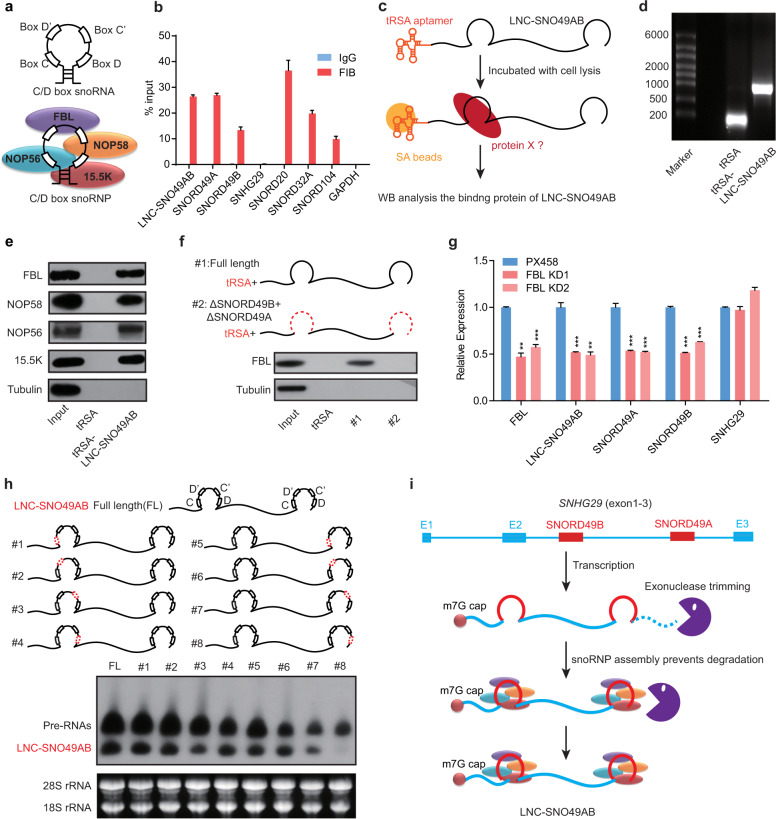
The role of SNORD49A/B in LNC-SNO49AB processing. **a** Schematic representation of C/D box snoRNA (upper) and snoRNP (below). **b** FBL RIP followed by RT-qPCR analysis of copurified RNA in RS4;11 cells. Representative results are shown from three replicates. **c** Schematic representation of the tRSA RNA pull-down assay. **d** Formaldehyde denaturing agarose gel analysis of the in vitro-transcribed tRSA and LNC-SNO49AB. Representative results are shown from three replicates. **e** The results of western blot showed that FBL, NOP58/56 and 15.5 K proteins in the cell lysis were precipitated by in vitro-transcribed LNC-SNO49AB. **f** Schematic of deletion mutants of LNC-SNO49AB (upper). The FBL binding capability of truncated LNC-SNO49AB is shown below. Tubulin was used as a negative control. **g** qRT-PCR analysis of FBL, LNC-SNO49AB, SNORD49A/B and SNHG29 in RS4;11 cells treated with FBL sgRNA. The empty PX458 vector was transfected as control. Data were normalized to *GAPDH* mRNA. Values are the means ± SEM of three independent experiments. *P < 0.05, **P < 0.01, and ***P < 0.001 by Student’s *t*-test. **h** Schematic of full-leng*t*h LNC-SNO49AB (upper) and deletions of box C/D (middle, #1–8, red dashed lines). Northern blot of LNC-SNO49AB and the indicated mutants(bottom), 28S and 18S rRNA were acted as the total RNA controls. Representative results are shown from three replicates. **i** Schematic of the biogenesis and processing of LNC-SNO49AB.

The unusual structure of LNC-SNO49AB leads to the question whether snoRNA is involved in the processing of LNC-SNO49AB. SNORD49A and SNORD49B belong to C/D box snoRNAs, which contain two boxes near their termini (box C and box D) and two boxes away from their termini (boxes D’ and C’)^8^ (Fig. 2a). Typically, the conserved motifs of C/D box snoRNAs directly bind core proteins (snoRNPs), including fibrillarin (FBL), NOP58, NOP56, and 15.5 K (Fig. 2a), which are essential for snoRNA processing and function^29,30^. Therefore, we asked if these core proteins interact with LNC-SNO49AB. RNA immunoprecipitation (RIP) assays showed that LNC-SNO49AB could be enriched by FBL, which is similar to SNORD49A and SNORD49B, while lncRNA SNHG29 transcribed by its host gene showed a lack of enrichment of FBL binding (Fig. 2b). Consistently, a tRSA-based RNA pull-down confirmed that LNC-SNO49AB could bind to the four core proteins that bind C/D box snoRNAs (Fig. 2c–e). In addition, when deleting both SNORD49A and SNORD49B domains of LNC-SNO49AB, the lnc-snoRNA could no longer interact with FBL (Fig. 2f), suggesting that these two snoRNA sequences determined the interaction between LNC-SNO49AB and the core C/D box snoRNA proteins. Importantly, suppression of FBL expression resulted in the dramatic downregulation of both snoRNAs and the lnc-snoRNA (Fig. 2g), indicating that the SNORD49A/B sequences could mediate snoRNP complex formation and is critical for the stability of LNC-SNO49AB.

It was previously reported that snoRNAs at the ends of lncRNAs protect the intronic sequences from exonuclease trimming after splicing, leading to the formation of snoRNA-related lncRNAs^5,13^. However, different from the previous findings^5,12^, SNORD49A and B are at the 3′-end and in the middle of LNC-SO49AB respectively, raising the question of how these two snoRNAs participate in LNC-SNO49AB formation. We sequentially deleted the C, D, C’ and D’ boxes of SNORD49B and SNORD49A to see which one is important for the lnc-snoRNA. We found that LNC-SNO49AB formation was completely eliminated only when deleting the D box of SNORD49A, the rest boxes did not show significant effects (Fig. 2h). In particular, deletion of the conserved motifs of SNORD49B did not impair the generation of LNC-SNO49AB. These results indicate that only snoRNAs at the ends are crucial for the lnc-snoRNA formation. Therefore, we proposed a model in which LNC-SNO49AB biogenesis is associated with intronic cleavage, a splicing-independent mechanism^31^. During transcription initiation, m7G is installed at the 5′ cap of LNC-SNO49AB cotranscriptionally. The sequence of unspliced intron 2 is retained in the pre-LNC-SNO49AB and is trimmed to the point where the exonuclease reaches the SNORD49A snoRNP, which prevents further degradation, thereby generating a snoRNA at the 3′-end (Fig. 2i). At the current stage of study, we do not know why lnc-snoRNA needs a snoRNA positioned in the middle.

### LNC-SNO49AB is highly expressed in leukemia and promotes cell proliferation in vitro and in vivo

**Fig. 3:**
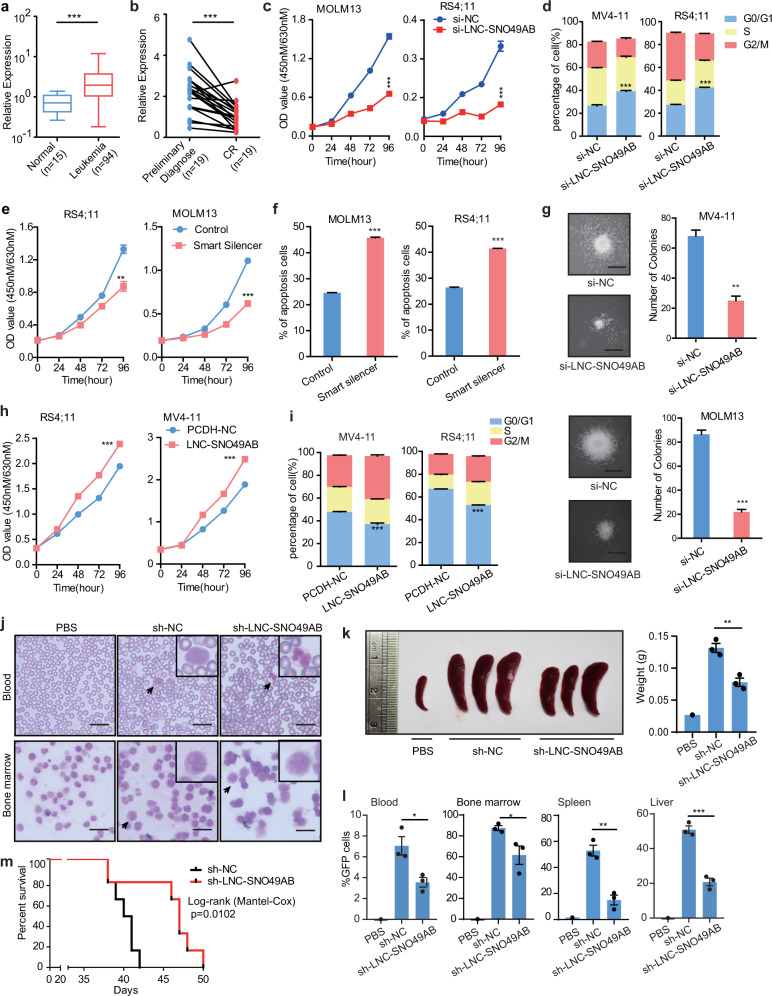
In vitro and in vivo studies showing LNC-SNO49AB functioned as a pro-leukemic lncRNA in leukemia. **a** qRT-PCR analysis of LNC-SNO49AB expression in normal (*n* = 15) and leukemia bone marrow (*n* = 94) samples. Data were normalized to *GAPDH* mRNA. ***P < 0.001 by Mann-Whitney test. **b** qRT-PCR analysis of LNC-SNO49AB in 19 pairs of patients with leukemia at preliminary diagnosis and complete remission (CR). Data were normalized to *GAPDH* mRNA. ***P < 0.001 by Wilcoxon matched-pairs signed rank test. **c**, **d** Effects of knocking **d**own LNC-SNO49AB expression by siRNA on cell proliferation (**c**) and the cell cycle (**d**). Values are the mean ± SEM of three independent experiments. ***P < 0.001 by Student’s *t* test. **e**, **f** Effects of knocking down LNC-SNO49AB expression by smart silencing of cell proliferation (**e**) and 2 μM ATO (arsenic trioxide)-induced apoptosis (**f**) in RS4;11 and MOLM13 cells. Values are the mean ± SEM of three independent experiments. **P < 0.01, and ***P < 0.001 by Student’s *t* test. **g** Morphology of colonies of leukemia cells upon siRNA-mediated knockdown of LNC-SNO49AB. Scale bars, 100 μm. Error bars reflect ±SEM of three independent experiments. **P < 0.01, ***P < 0.001. **h**, **i** Effects of forced expression of LNC-SNO49AB on cell proliferation (**h**) and arrest of the cell cycle (**i**) in leukemia cells. Values are the mean ± SEM of three independent experiments. ***P < 0.001 by Student’s *t* test. **j** Wright-Giemsa staining of peripheral blood (*P*B) and BM of mouse recipients of PBS, sh**-**NC cells or sh-LNC-SNO49AB RS4;11 cells. RS4;11 cells were indicated by the black arrows. Scale bars, 50 μm. **k** Spleen photographs from transplant mice (left) and the weight statistic at the time of the final analysis (right). Values are the mean ± SD of three independent experiments. **P < 0.01 by Student’s *t* test. **l** Flow cytometry analysis of GFP+ cells in blood, BM, spleen and liver. Values are the mean ± SEM of three independent experiments. *P < 0.05, and ***P < 0.001 by Student’s *t* test. **m** Effect of knocking down LNC-SNO49AB expression on the survival of the recipient mice. Kaplan-Meier curves are shown for two cohorts of transplanted mice, sh-NC and sh-LNC-SNO49AB cells. Six mice per group. The *P*-values were calculated by log-rank test.

We next investigated the expression pattern and function of LNC-SNO49AB. Both SNORD49A and SNORD49B have been shown abnormally expressed in leukemia^26^, and thus we mainly investigated the potential role of LNC-SNO49AB in leukemia. In analysis of our in-house clinical samples with 15 normal control and 94 leukemia samples with 84 AML and 10 ALL (the detailed clinical parameters are presented in Supplementary Table S1), we found that LNC-SNO49AB displayed a significantly higher expression level both in the ALL and AML patient groups than that in the normal group (Fig. 3a and Supplementary Fig. S2a). A high copy number of LNC-SNO49AB was also detected in a panel of leukemia cell lines (Supplementary Fig. S2b, c). When classifying patient samples into different subtypes according to mutational background with different types of chromosomal rearrangements, we found that LNC-SNO49AB expression did not correlate with a specific mutational background, instead, it shows a widely high expression level in leukemia (Supplementary Fig. S2d). In a 19 paired preliminary diagnosis-complete remission (CR) samples, we showed that CR samples showed a lower expression level of LNC-SNO49AB (Fig. 3b). ROC analysis indicated that LNC-SNO49AB could efficiently predict CR (AUC = 0.8366, 95% CI: 0.7035–0.9696, *P* < 0.001), with 78.95% sensitivity and 84.21% specificity at the optimal likelihood ratio (Supplementary Fig. S2e). Moreover, high level of LNC-SNO49AB was correlated with high-risk, poor-prednisone response and unfavorable recurrence-free survival (RES) (Supplementary Fig. S2f–h). Together, these results indicate that LNC-SNO49AB was generally highly expressed in various types of leukemia and associated with leukemia progression and therapeutic outcomes, highlighting its potential oncogenic role.

We next investigated the function of LNC-SNO49AB in leukemia. Introduction of siRNAs targeting LNC-SNO49AB in multiple leukemia cells downregulated LNC-SNO49AB expression (Supplementary Fig. S3a) and significantly reduced cell proliferation in the cell counting test (Fig. 3c and Supplementary Fig. S3b) and EdU incorporation assay (Supplementary Fig. S3c). We also found that silencing LNC-SNO49AB caused cell cycle arrest (Fig. 3d and Supplementary Fig. S3d) and induced the apoptosis of various leukemia cells (Supplementary Fig. S3e). Similar phenomena were observed when LNC-SNO49AB was knocked down with a mix of additional siRNAs and antisense oligonucleotides (smart silencer) targeting sequences different from that of by si-LNC-SNO49AB that we previously used (Fig. 3e, f and Supplementary Fig. S3f–h). In addition, clonogenicity was significantly impaired after LNC-SNO49AB expression was reduced (Fig. 3g and Supplementary Fig. S3i). Moreover, overexpressing LNC-SNO49AB in leukemia cell lines (Supplementary Fig. S4a showed the overexpression efficiency), including NB4, MV4-11 and RS4;11 cells, promoted cell proliferation (Fig. 3h and Supplementary Fig. S4b), released cell cycle arrest (Fig. 3i and Supplementary Fig. S4c), and inhibited apoptosis (Supplementary Fig. S4d).

We further investigated the ability of LNC-SNO49AB to regulate leukemia progression in patient-derived leukemia samples and a mouse model. Primary leukemia cells were first isolated from the bone marrow of four patients with newly diagnosed acute leukemia (numbers H485, H521, H527 and H686) (Supplementary Fig. S4e). Silencing LNC-SNO49AB expression by siRNAs significantly impaired the DNA synthesis rate and increased the cell apoptosis rate of all the primary leukemia cells (Supplementary Fig. S4f–h), which is concordant with the phenotypes observed in cell lines. We also examined the effect of LNC-SNO49AB depletion on leukemic progression in vivo by injecting GFP^+^ RS4;11 cells transduced with sh-NC or sh-LNC-SNO49AB in the tail vein of mice (Supplementary Fig. S5a). Supplementary Fig. S5b, c showed knocked down efficiencies of LNC-SNO49AB. The success of mouse model establishment was confirmed by the appearance of RS4;11 cells in blood and bone marrow (BM) (Fig. 3j). Reducing LNC-SNO49AB caused a decrease in leukemia engraftment in the spleen, BM, and liver, as determined by haematoxylin & eosin (H&E) staining (Supplementary Fig. S5d) and the reduced spleen size and weight of xenograft tumors (Fig. 3k). Similarly, flow cytometry analysis revealed a sharp decrease in the infiltration of RS4;11 cells in blood, BM, spleen, and liver (Fig. 3l and Supplementary Fig. S5e). Furthermore, mice injected with sh-LNC-SNO49AB cells displayed better survival outcomes (Fig. 3m). In contrast, overexpression of LNC-SNO49AB by PCDH-LNC-SNO49AB significantly promoted hematopoietic malignancy in the recipient mice (Supplementary Fig. S5f–h). Taken together, both in vitro and in vivo experimental results have demonstrated that LNC-SNO49AB plays an important oncogenic role in leukemia progression.

### LNC-SNO49AB interacts with ADAR1 in the nucleolus

**Fig. 4:**
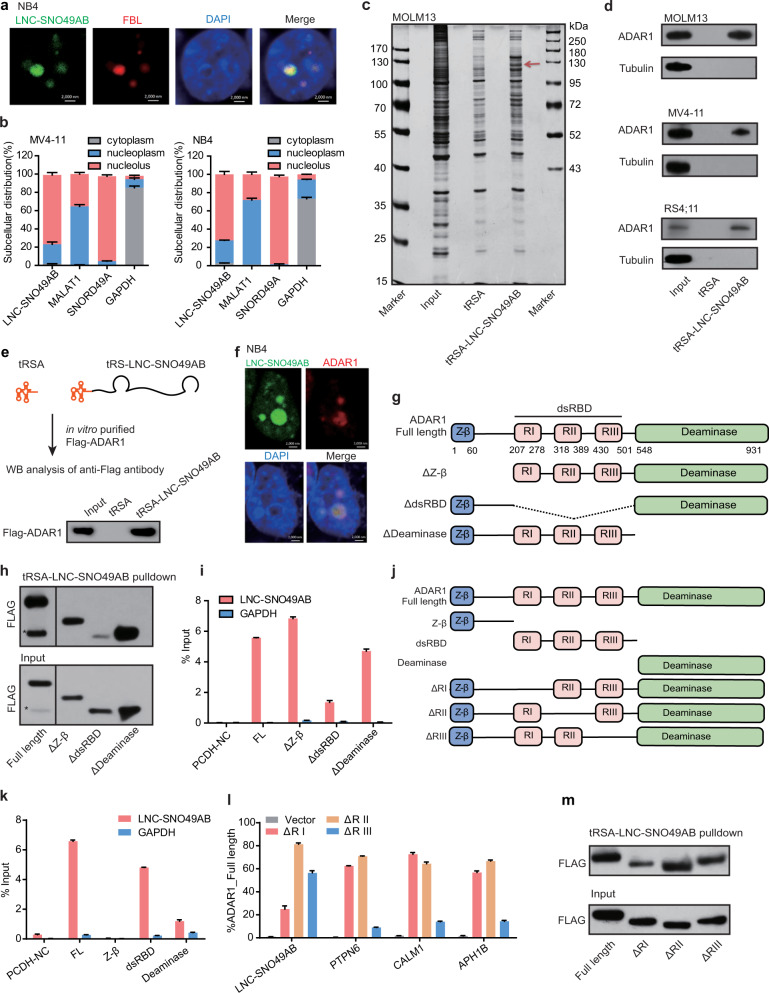
LNC-SNO49AB binds ADAR1 in the nucleolus. **a** Representative images of dcas13-mediated LNC-SNO49AB labeling (green); LNC-SNO49AB colocalized with FBL (red) in NB4 cells. Scale bars, 2 μm. **b** Subcellular localization of LNC-SNO49AB in MV4-11 and NB4 cells. MALAT1, SNORD49A and *GAPDH* RNAs were used as controls for nucleolar, nuclear, and cytoplasmic fractions, respectively. **c** Silver staining of proteins pulled down by in vitro transcribed tRSA and tRSA-LNC-SNO49AB from total protein extracts of MOLM13 cells. Red arrow shows the specific band. **d** ADAR1 was pulled down by tRSA-LNC-SNO49AB but not tRSA in MOLM13, MV4-11 and RS4;11 cells, as determined by western blotting. **e** In vitro RNA pull-down assay with purified recombinant Flag-ADAR1 and tRSA-LNC-SNO49AB. Representative results are shown from three replicates. **f** Representative images of dcas13-mediated LNC-SNO49AB labeling (green); LNC-SNO49AB colocalized with ADAR1 (red) in NB4 cells. Scale bars, 2 μm. **g** Schematic representation of the ADAR1 deletion mutants used in the RNA pull-down and RIP assays shown in **h** and **i**. **h** Western blot of the indicated Flag-tagged ADAR1 deletion mutants retrieved by in vitro transcribed tRSA-LNC-SNO49AB obtained from 293T cell extracts. *Represented the non-specific band. Representative results are shown from three replicates. **i** qRT-PCR analysis of endogenous LNC-SNO49AB enriched by the indicated Flag-tagged ADAR1 deletion mutants in 293T cells. *GAPDH* mRNA served as the negative control. Representative results are shown from three replicates. **j** Schematic representation of the ADAR1 deletion mutants used in the RNA pull-down and RIP assays shown in **k**–**m**. **k** qRT-PCR analysis of endogenous LNC-SNO49AB enriched by the indicated Flag-tagged ADAR1 deletion mutants in 293T cells. *GAPDH* served as the negative control. Representative results are shown from three replicates. **l** qRT-PCR analysis of endogenous LNC-SNO49AB enriched by the indicated Flag-tagged ADAR1 deletion mutants in 293T cells. PTPN6, CALM1 and APH1B are the representative targets of ADAR1. Representative results are shown from three replicates. **m** Western blot of the indicated Flag-tagged ADAR1 deletion mutants retrieved by in vitro transcribed tRSA-LNC-SNO49AB obtained from 293T cell extracts. Representative results are shown from three replicates.

Next, we explored the molecular mechanism underlying the oncogenic activity of LNC-SNO49AB in the disease. We first examined the subcellular localization of LNC-SNO49AB. RNA imaging by CRISPR-Cas13 system^32,33^ (Fig. 4a) and subcellular fraction analysis (Fig. 4b and Supplementary Fig. S6a) showed that LNC-SNO49AB was mainly located in the nucleus, especially in the nucleolus. NEAT1, enriched in paraspeckle^34^, was used as a negative control for nucleolus-located lncRNAs (Supplementary Fig. S6b). Although the canonical role of C/D box snoRNA is mediating the 2′-O-methylation of rRNA in the nucleolus, we found no alteration of the methylation abundance at 28 S rRNA cytidine^4426^ (predicted to be guided by SNORD49A and SNORD49B^35,36^) or global rRNA when silencing LNC-SNO49AB (Supplementary Fig. S6c–e). These results suggest that LNC-SNO49AB might function through a novel molecular mechanism in the nucleolus, independent of snoRNA-guide rRNA modification.

We then performed tRSA RNA pull-down assays^37,38^ to identify the potential proteins associated with LNC-SNO49AB in leukemia. The tRSA-LNC-SNO49AB-specific bands at 100~130 kDa were repeatedly observed in two independent RNA pull-down experiments using leukemia cell lines (Fig. 4c and Supplementary Fig. S6f), then they were subjected to mass spectrometry (MS) (Supplementary Table S2). Among the proteins identified by MS, one specific protein, ADAR1, a known RNA-editing enzyme located in the nucleolus^39–41^, attracted our attention (Supplementary Fig. S6g). The interaction between ADAR1 and LNC-SNO49AB was further validated by RNA pull-down followed by western blotting. As shown in Fig. 4d, endogenous ADAR1 interacted with tRSA-LNC-SNO49AB but not tRSA. A similar interaction was readily detected by anti-Flag antibodies in RIP assays of 293T cells transiently expressing the Flag-ADAR1 protein (Supplementary Fig. S6h). Moreover, in vitro transcribed LNC-SNO49AB could bind to purified Flag-ADAR1 protein in a cell-free system (Fig. 4e). Immunofluorescence assays also showed that ADAR1 locates in the nucleolus, and LNC-SNO49AB and ADAR1 predominantly colocalized in the nucleolus (Fig. 4f and Supplementary Fig. S6i). Together, these data indicate that LNC-SNO49AB binds directly to ADAR1.

ADAR1, belonging to the ADAR family, is critical for the majority of A-to-I editing activity in mammals^42,43^. ADAR1 contains three main domains, including a Z-DNA-binding domain in the N-terminus; three dsRNA-binding domains (dsRBDs), namely, RI, RII and RIII, in the middle; and a catalytic deaminase domain in the C-terminus^42^. We next conducted an RNA pull-down assay using a series of Flag-tagged *ADAR1* with deletions based on structural features to map its functional motif, which was associated with LNC-SNO49AB (Fig. 4g). The results showed that deletion of dsRBDs of ADAR1 strongly impaired the ADAR1 interaction with LNC-SNO49AB (Fig. 4h), while the remaining two domains exerted negligible effects. Accordingly, a RIP analysis revealed the high affinity of dsRBDs for LNC-SNO49AB (Fig. 4i), and a truncated ADAR1 fragment with three dsRBDs bound to LNC-SNO49AB efficiently (Fig. 4j, k), suggesting that dsRBDs are necessary and sufficient to mediate the interaction of ADAR1 and LNC-SNO49AB. Within the dsRBDs, deletion of RI significantly decreased the interaction between ADAR1 and LNC-SNO49AB, while RII and RIII showed less efficiency in binding LNC-SNO49AB (Fig. 4l, m), indicating that RI of the dsRBDs in ADAR1 is critical for LNC-SNO49AB binding.

It is known that the dsRBDs of ADAR1 can make direct contact with dsRNAs to edit these dsRNAs^44,45^. However, no A-to-I editing sites of LNC-SNO49AB were found in three major RNA-editing databases RADAR^46^, http://rnaedit.com/; REDIportal^47^, http://srv00.recas.ba.infn.it/atlas/; and DARNED^48^, http://darned.ucc.ie/about/, indicating that LNC-SNO49AB was not the gene targeted for editing by ADAR1. In agreement with this result, deletion of RIII diminished ADAR1 binding of its known RNA-editing substrate but had less effect on its binding to LNC-SNO49AB, as shown in the RIP assay (Fig. 4l). In addition, LNC-SNO49AB did not regulate the protein levels of ADAR1 (Supplementary Fig. S6j), and silencing ADAR1 did not affect the LNC-SNO49AB expression (Supplementary Fig. S6k). Together, these results raise a question, what is the biological role of specific LNC-SNO49AB binding to dsRBDs in ADAR1?

### LNC-SNO49AB promotes ADAR1 dimerization

**Fig. 5:**
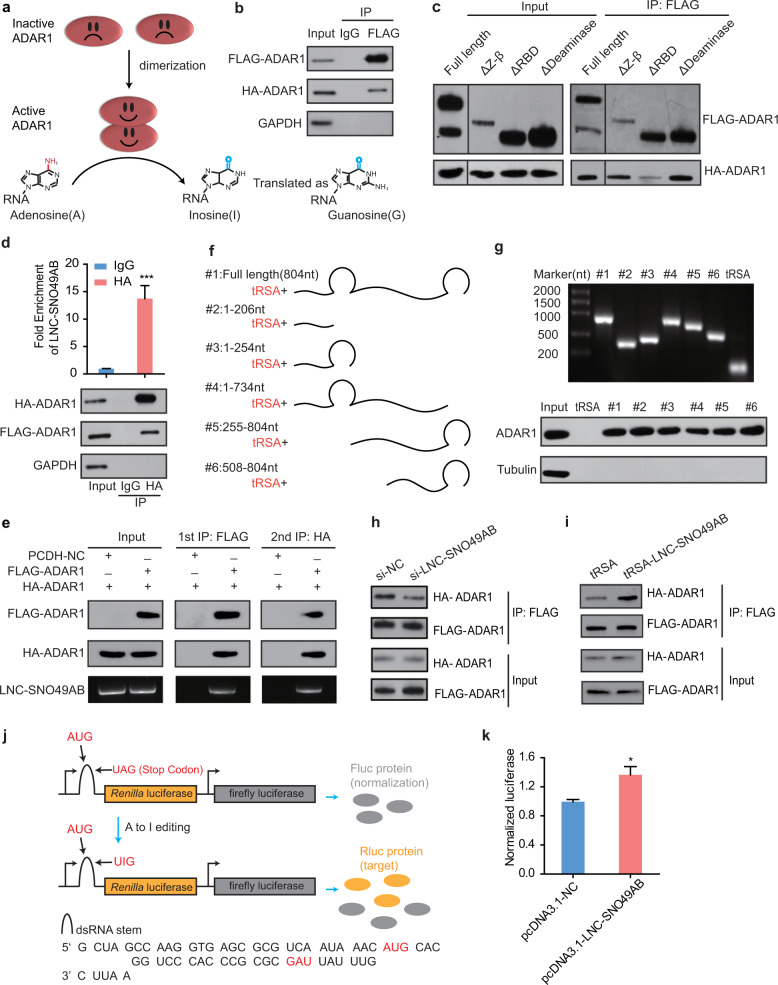
LNC-SNO49AB regulates the dimerization of ADAR1. **a** Schematic representation of the function of ADAR1. The editing activity of ADAR1 requires homodimerization. **b** Immunoprecipitation of FLAG-ADAR1 with HA-ADAR1 was performed using 293T cells. Immunoprecipitation of FLAG-ADAR1 was performed using 293T cells. IgG, immunoglobulin G. Representative results are shown from three replicates. **c** Immunoprecipitation of the indicated FLAG-ADAR1 deletion mutants with HA-ADAR1 was performed using 293T cells. Representative results are shown from three replicates. **d** FLAG-ADAR1 and LNC-SNO49AB were coprecipitated with HA-ADAR1 in whole-cell lysates of 293T cells. **e** FLAG-ADAR1, HA-ADAR1 and LNC-SNO49AB were coprecipitated with an anti-FLAG antibody, and after elution with FLAG peptide, all were further coprecipitated with an anti-HA antibody in the resultant precipitates. Representative results are shown from three replicates. **f** Schematic of deletion mutants of LNC-SNO49AB. **g** Immunoblot detection of endogenous ADAR1 retrieved by in vitro-transcribed tRSA-tagged LNC-SNO49AB sections from MOLM13 cell lysates. All of the LNC-SNO49AB sections presented significant enrichment. Tubulin as the negative control. **h**, **i** LNC-SNO49AB promoted the binding between recombinant FLAG-ADAR1 and HA-ADAR1 in vivo (**h**) and in vitro (**i**). Representative results are shown from three replicates. **j** Schematic overview of the RNA-editing reporter assay. **k** Normalized luciferase in 293T cells transfected with LNC-SNO49AB and control. Values are the means ± SEM of three independent experiments. *P < 0.05 by Student’s *t*-test. Representative results are shown from three replicates.

The RNA-editing activity of ADAR1 depends on homodimerization^41,43^ (Fig. 5a). Indeed, we found that ectopically expressed Flag-ADAR1 coprecipitated with HA-tagged ADAR1 in 293T cells (Fig. 5b). Importantly, deleting the dsRBDs of Flag-ADAR1 severely impaired of the Flag-ADAR1 interaction with full-length HA-ADAR1 (Fig. 5c), suggesting a pivotal role of dsRBDs in dimerization. Thus, we hypothesized that LNC-SNO49AB may affect the homodimerization of ADAR1 by binding the dsRBDs. The premise of this hypothesis is that LNC-SNO49AB can form a complex with the ADAR1 homodimer. We first overexpressed FLAG-ADAR1, HA-ADAR1 and LNC-SNO49AB simultaneously in 293T cells, and then an HA-ADAR1 coimmunoprecipitation assay showed that both FLAG-ADAR1 and LNC-SNO49AB were significantly enriched (Fig. 5d). Moreover, when performing a two-step RIP assay, we found that the first-phase of the RIP using anti-FLAG antibodies captured high levels of HA-ADAR1 and LNC-SNO49AB in addition to FLAG-ADAR1, whereas antibodies against the HA epitope tag precipitated HA-ADAR1 together with FLAG-ADAR1 and LNC-SNO49AB (Fig. 5e), indicating that LNC-SNO49AB and dimerized ADAR1 formed a functional structure. To further explore the potential motifs of LNC-SNO49AB that function in ADAR1 homodimerization, we dissected the LNC-SNO49AB sequence (Fig. 5f). Strikingly, an RNA pull-down assay showed that every truncated LNC-SNO49AB fragment interacted with ADAR1 in MOLM13 leukemia cells (Fig. 5g), implying that LNC-SNO49AB contains several ADAR1 binding sites over its entire sequence. Next, we explored whether LNC-SNO49AB affected the dimerization of ADAR1. As a result, LNC-SNO49AB knockdown decreased the relative amount of HA-ADAR1 associated with FLAG-ADAR1 in 293T cells, whereas LNC-SNO49AB overexpression increased the interaction between Flag-ADAR1 and HA-ADAR1 in a cell-free system (Fig. 5h, i), indicating that LNC-SNO49AB stabilized ADAR1 dimerization. Since the RNA-editing activity of ADAR1 requires dimerization, we designed an editing reporter vector based on a dual luciferase system to investigate whether LNC-SNO49AB affects the activity of ADAR1^49^ (Fig. 5j). A stop codon was embedded near the start codon of *Renilla* luciferase (hRluc) in a short stretch of dsRNA, resulting in a premature stop to translation. Importantly, this dsRNA structure was recognized as an editing substrate by ADAR1, and RNA A-to-I editing converted the stop codon (UAG) into UIG, allowing the translation of mature hRluc. Thus, the expression of hRluc was editing dependent. The reporter vector was transfected with either pcDNA3.1 or pcDNA3.1-LNC-SNO49AB into 293T cells, and the frequency of A-to-I RNA editing was measured by luciferase activity. We found that LNC-SNO49AB overexpression produced more hRluc than the control (Fig. 5k), suggesting a role of LNC-SNO49AB in regulating ADAR1 activity. Together, these data demonstrate that LNC-SNO49AB elevates ADAR1 activity by promoting its homodimerization.

### The oncogenic role of LNC-SNO49AB partially depends on its regulation of ADAR1 activity

**Fig. 6:**
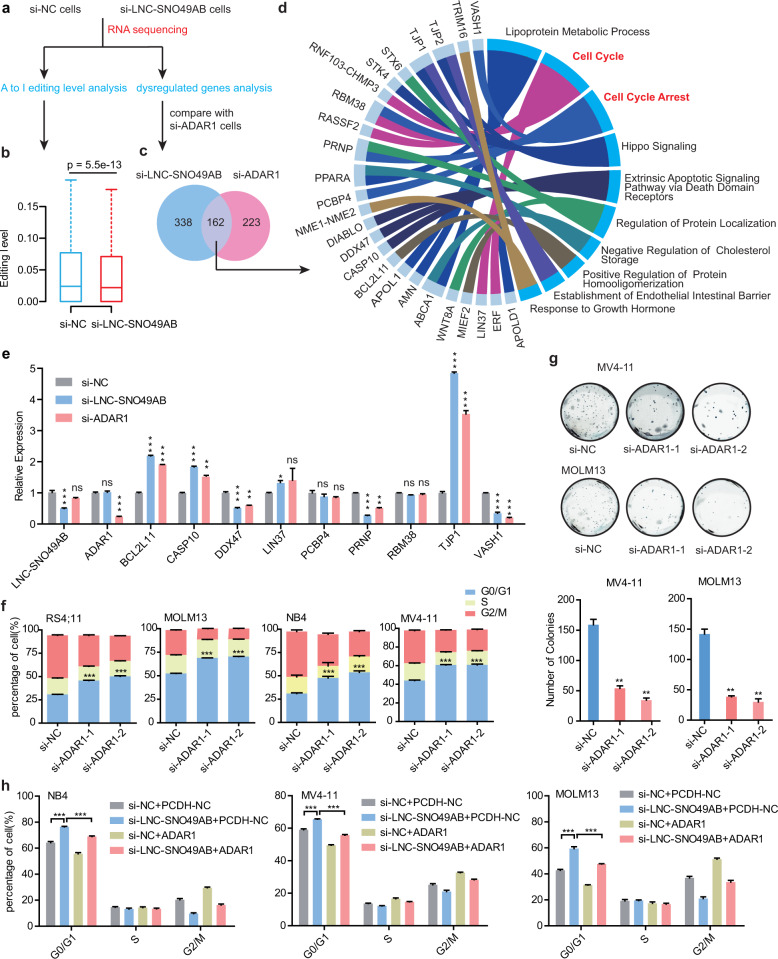
LNC-SNO489AB is required for ADAR1 to play a regulatory role in cancer. **a** The analysis workflow of the A-to-I editing rate and dysregulated genes when LNC-SNO49AB is silenced in RS4;11. **b** The box plots showed a global decrease of RNA A-to-I editing levels in si-LNC-SNO49AB group. **c** Venn diagram showing overlap of genes that are dysregulated both in si-LNC-SNO49AB cells and si-ADAR1 cells. **d** GO analysis of 162 genes that are dysregulated in both si-LNC-SNO49AB cells and si-ADAR1 cells. **e** qRT-PCR validation of genes that were dysregulated in both si-LNC-SNO49AB cells and si-ADAR1 cells. Gene expression was normalized to *GAPDH* mRNA. Values are the means ± SEM of three independent experiments. ns, not significant, *P < 0.05, **P < 0.01, and ***P < 0.001 by Student’s *t*-test. **f** Cell cycle analysis when ADAR1 was silenced via two independent siRNAs. Values are the means ± SEM of three independent experiments. ***P < 0.001 by Student’s *t*-test. **g** Colony numbers from leukemia cells with ADAR1 knocked down by siRNAs compared with the control (si-NC). Values are the means ± SEM of three independent experiments. **P < 0.01 by Student’s *t*-test. **h** Effects of ADAR1 overexpression and/or LNC-SNO49AB knockdown on the cell cycle in NB4, MV4-11 and MOLM13 cells. Values are the means ± SEM of three independent experiments. ***P < 0.001 by Student’s *t*-test.

Elevated activity of RNA A-to-I editing is observed in various cancer types and is associated with poor prognosis, and systematic exploration of cancer vulnerabilities informs the dependency of ADAR1 in a subset of cancer cells^50–52^. Thus, we hypothesized that LNC-SNO49AB might elevate the RNA A-to-I editing rate by promoting ADAR1 homodimerization to exert its oncogenic effects. Unbiased transcriptome profiling was performed in RS4;11 cells transfected with si-NC or si-LNC-SNO49AB to investigate the change in RNA A-to-I editing rates (Fig. 6a). We observed a global decrease (*P* = 5.5e−13) in the RNA A-to-I editing rate when LNC-SNO49AB was silenced (Fig. 6b), confirming that LNC-SNO49AB regulates RNA-editing activity in leukemia. Candidates showing major variation in editing were experimentally verified (Supplementary Fig. S7a). We then analysed the potential signaling pathway of 499 genes with significant variation in RNA-editing rate and found that most of the genes were significantly enriched in the cell cycle and DNA repair process (Supplementary Fig. S7b, Table S3). Previous studies reported that RNA A-to-I editing has a profound impact on RNA translation, splicing and stability^53,54^. It has also been shown that the A-to-I sites are largely distributed in the 3′-UTR of mature mRNA^55^. Remarkably, editing the 3′-UTR can create or destroy microRNA recognition, which may be involved in either translation repression or mRNA degradation^56^. Combined with the RNA expression level identified in the RNA-seq data, we found that oncogenes, BRI3BP^57^, involved in apoptosis, was hypoedited in the 3′-UTR in the si-LNC-SNO49AB group and were significantly downregulated in both si-LNC-SNO49AB and si-ADAR1 groups (Supplementary Fig. S7c). To further investigate the potential downstream genes of LNC-SNO49AB and ADAR1 in leukemia, we compared the RNA-seq data between si-ADAR1 group and si-LNC-SNO49AB group. 162 of 384 dysregulated genes in the si-ADAR1 group were also found to be dysregulated in the si-LNC-SNO49AB group (|fold change (FC)| > 2, FDR < 0.05) (Fig. 6c and Supplementary Tables S4, S5), further confirming that LNC-SNO49AB can promote leukemic progression by regulating ADAR1 activity. Interestingly, many common downstream genes of LNC-SNO49AB and ADAR1 were significantly enriched in gene ontology (GO) terms, including the cell cycle, Hippo signaling, and lipoprotein metabolic processes (Fig. 6d). We also found that knocking down LNC-SNO49AB or ADAR1 significantly regulated the expression of these potential target genes, further suggesting the common roles of SNO49AB and ADAR1 in cell viability (Fig. 6e).

**Fig. 7:**
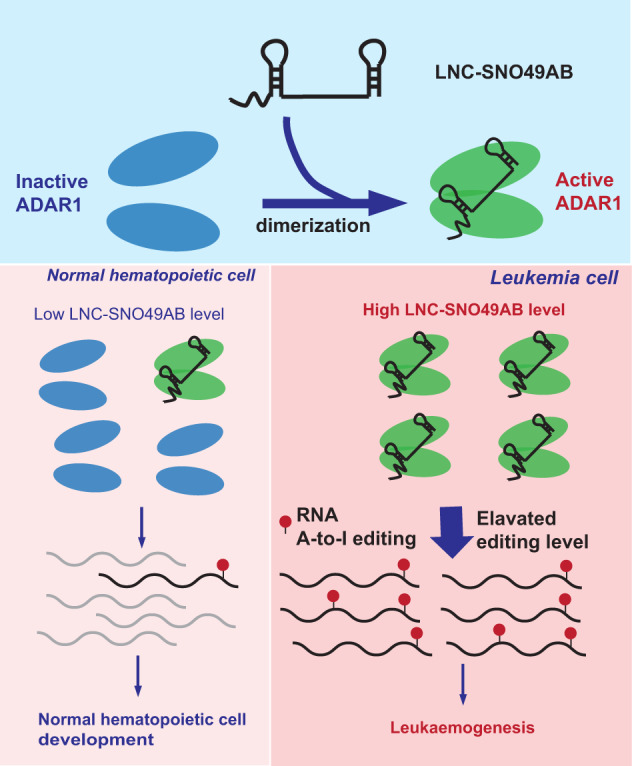
Working model of the role and underlying mechanism of LNC-SNO49AB in leukaemogenesis. LNC-SNO49AB improves ADAR1 dimerization and its editing activity. Compared to normal hematopoietic cell, leukemia cell has a higher expression of LNC-SNO49AB, which can significantly enhance the activation of ADAR1-mediated RNA A-to-I editing.

To further investigate whether the function of LNC-SNO49AB in the disease is ADAR1 dependent, we next knocked down ADAR1 in leukemia cells. Specific siRNAs targeting ADAR1 reduced the levels of ADAR1 protein (Supplementary Fig. S7d) and resulted in cell cycle arrest (Fig. 6f) and impaired clonogenic growth (Fig. 6g), confirming that ADAR1 is a tumor-promoting gene in leukemia. Similarly, forced expression of ADAR1 recapitulated the phenotypes acquired through LNC-SNO49AB overexpression (Supplementary Fig. S7e, f). Furthermore, the effects of LNC-SNO49AB knockdown were largely rescued by forced expression of ADAR1 (Fig. 6h) and ADAR1 ablation partially counteracted the effect of LNC-SNO49AB overexpression on cell cycle promotion (Supplementary Fig. S7g). Collectively, our data demonstrate that LNC-SNO49AB promotes hematopoietic malignancy by enhancing the activation of ADAR1-mediated RNA A-to-I editing. The proposed working model is summarized in Fig. 7.

## Discussion

LncRNAs, potential key elements in gene expression regulation networks, are reshaping modern oncology^58–60^. Due to the association between lncRNA signatures and pathological processes and the ability to profoundly influence carcinogenesis, lncRNAs can be utilized as potential cancer biomarkers and therapeutic targets in the clinic^61,62^. In this study, we identified an abundant lncRNA, LNC-SNO49AB, containing the sequences of SNORD49A and SNORD49B in leukemia. The expression of LNC-SNO49AB was correlated with therapeutic outcomes, and silencing LNC-SNO49AB impaired leukemia progression in vitro and in vivo. We further determined that LNC-SNO49AB is a stable lncRNA and interfering the lncRNA did not impair the expression level of its host gene and mature snoRNAs in the same gene locus, indicating that LNC-SNO49AB may represent a potential prognostic biomarker and has potential application values of targeted therapy in leukemia.

As a class of newly identified stable lncRNAs with unique structures, snoRNA-related lncRNAs have attracted much attention. While most lncRNAs are processed to be 5′ m7G capped and 3′ polyadenylated, the ends of snoRNA-related lncRNAs contain snoRNA sequences^5^. The majority of snoRNAs are encoded within the introns of their host genes, which are trimmed^11,63,64^. When two snoRNAs are embedded within a single intron, trimming can result in a sno-lncRNA, which consists of an intronic lncRNA flanked by two snoRNAs^6,63^. Notably, only a small fraction of known snoRNAs appear as a pair in the same intron, whereas the majority, ~85%, of snoRNAs are embedded in introns individually^6,63^. However, whether the snoRNAs located in introns individually are capable of generating snoRNA-related lncRNAs is largely unknown. Here, we identified a 5′-end m7G and 3′-end snoRNA lncRNA, LNC-SNO49AB, with SNORD49B in the middle and SNORD49A at the 3′ end. In particular, only one snoRNA (SNORD49A) is required for LNC-SNO49AB biogenesis, raising the possibility that snoRNAs embedded in introns individually might mediate snoRNA-related lncRNA generation. Interestingly, in addition to snoRNAs, microRNAs, other types of small RNAs located within introns, were reported to form lncRNAs called lnc-pri-miRNAs^65,66^. Lnc-pri-miRNAs are flanked by miRNAs at the 3′ end and thus lack poly(A)-tails^65,66^. We believe that the total number and diversity of lncRNAs with small RNA-ends will continue to climb, because of more-sensitive RNA sequencing and new computational pipelines, especially the improvement in non-polyadenylated RNA-seq.

Assembly into macromolecular complexes is crucial for most proteins to become functional, which requires that subunits find each other precisely and efficiently in the crowded cellular environment^67–69^. Recent studies indicated that oligomerization is facilitated by timely coupling translation and subunit interaction (cotranslational assembly)^69^. Here, our results demonstrated that LNC-SNO49AB promotes ADAR1 dimerization at the posttranslational level, as shown by (1) LNC-SNO49AB interacting with ADAR1 in nucleoli; (2) LNC-SNO49AB forming a functional structure with an ADAR1 dimer; and (3) silencing of LNC-SNO49AB diminishing the interaction between the ADAR1s, inhibiting dimerization. Thus, LNC-SNO49AB acts as an RNA scaffold to facilitate the association between ADAR1s forming dimers posttranslationally. Similar to LNC-SNO49AB, a few lncRNAs have also been reported to be involved in protein complex formation^70^. Although co-translational assembly is an efficient method, lncRNA-mediated homodimerization or multimerization may be an important mechanism for protein activity control at the posttranslational level in response to cellular stresses and cancer progression since lncRNA expression levels vary^71,72^. This work will inspire future investigations to dissect the effects of lncRNAs on protein assembly at different developmental stages and in various diseases.

Cancer is driven by alterations in genomic information. The recently coined term ‘epitranscriptome’ describes RNA modifications, including RNA editing and methylation, that induce relevant changes to the transcriptome^73–75^. Despite increasing evidence showing that the epitranscriptome contributes to oncogenesis through the determination of RNA function and gene expression diversity^74^, our understanding of how it can be dynamically controlled is limited. Two antisense lncRNAs, FOXM1-AS^76^ and PCA3^77^, have been reported to specifically regulate the m6A and A-to-I editing of their sense protein-coding genes by recruiting the m6A demethylases ALKBH5 and ADAR1, respectively, showing that lncRNAs can give rise to the specificity of RNA modification. However, the epitranscriptome (i.e., m6A, A-to-I editing, pseudouridine, etc.) has often been shown to be globally dysregulated in cancer^78–80^. Therefore, it is important to determine the global effects of the epitranscriptome and its associated machinery. In this study, we identified that targeting LNC-SNO49AB decreases the global A-to-I RNA-editing rate by impairing ADAR1 dimerization, revealing a previously unrecognized strategy by which lncRNAs regulate the global epitranscriptome in cancer. Notably, very recent study demonstrated that endogenous ADAR-mediated RNA editing can persistently edit the target RNAs with oligonucleotides AIMers in non-human primate liver and has enormous potentials for specific cancer therapies in the nucleic acid-editing level^81^. In this study, we revealed that LNC-SNO49AB can specifically regulate the activity of ADAR1, which may provide a novel insight into the precision regulation of ADAR1-based RNA editing therapeutic strategy. It may be promising method of combining AIMers and LNC-SNO49AB overexpression to improve the efficiency in this ADAR1-based approach.

In summary, we identified a novel lncRNA, LNC-SNO49AB, with a 5′ m7G and 3′ snoRNA structure and revealed the mechanism of LNC-SNO49AB dependence on its ADAR1 interaction that promotes ADAR1 dimerization and elevates RNA A-to-I editing rates to enhance leukaemogenesis. These findings broaden our understanding of lncRNA diversity and provide a potential therapeutic target for leukemia treatment.

## Materials and methods

### Cell culture and treatment

Human leukemia cell lines RS4;11, SUPB15, CCRF-CEM, MOLM13, THP1, MV4-11, NB4, HL60 and human embryonic kidney cell line HEK293T were obtained from American Type Culture Collection (ATCC) and grown according to standard protocols. The primary cells were culture in IMDM (HyClone) supplemented with 10% FBS. All cells were culture at 37 °C in a 5% CO_2_ atmosphere. Transcription was blocked by adding 2 μg/mL actinomycin D or dimethylsulphoxide (Sigma) as a control to the cell culture medium.

### Leukemia patient samples collection

The clinical leukemia samples were obtained at the time of diagnosis and with informed consent from the First Affiliated Hospital of Sun Yat-sen University. Sample collection was approved by the Ethics Committee of the First Affiliated Hospital of Sun Yat-sen University. The study was conducted in accordance with the Declaration of Helsinki. The detail clinicopathological characteristics of the patients were summarized in Supplementary Table S1. The leukemia samples were stored in liquid nitrogen until used.

### RNA preparation and qRT–PCR

Total RNA was extracted from bone marrow and cell samples using an Invitrogen™ TRIZOL according to the manufacturer’s instructions. All RNA samples were stored at –80 °C before reverse transcription and quantitative RT-PCR. RNA was reverse transcribed into cDNA with the PrimeScript^®^ RT reagent Kit with Gdna Eraser (Takara, Japan). Quantitative RT-PCR was performed using the SYBR Premix ExTaq real-time PCR Kit (Takara, Japan) according to the manufacturer’s instructions. Data were normalized to GAPDH expression as a control. The relative expression level for LNC-SNO49AB and other lncRNA or mRNA was determined using the 2^−ΔΔCt^ method. The primers are listed in Supplementary Table S6.

### 5′ RACE and 3′ RACE

Total RNA from RS4;11 cells was extracted using TRIzol according to the manufacturer’s guidelines. The 5′ and 3′ ends of Cdna were acquired using a FirstChoice^®^ RLM-RACE Kit (Invitrogen™) according to the manufacturer’s instructions. Notably, in 3′ RACE assay, RNA was reverse transcribed by TransScript^®^ miRNA First-Strand cDNA Synthesis SuperMix (TransGen Biotech) to add polyA tails. PCR products were obtained and then cloned into Peasy-T3 (TransGen Biotech, China) for further sequencing.

### Cell proliferation, cell cycle, cell apoptosis, Edu, and CFU assay

Cell proliferation was measured using Cell Counting Kit-8 (Dojindo Molecular Technologies, China). Cells were seeded at a density of 20,000 cells per well in 100 μL of complete medium in 96-well plates. Absorbance was measured by a VICTOR™ X5 Multilabel Plate Reader (PerkinElmer, USA) at wavelengths of 480 and 630 nm at 0, 24, 48, 72, and 96 h. For the cell cycle and apoptosis assay, cells were collected and washed once by PBS. Cell pellets were resuspended in 0.5 μL of PI/Rnase staining buffer or Annexin V/PI staining buffer (Dojindo Molecular Technologies, China), respectively, and incubated for 30 min at room temperature. Cells were immediately measured and analyzed using flow cytometer (BD Biosciences, USA). For CFU assay, leukemia cells were plated into methylcellulose at a density of 500–1000 cells per 1.5 μL methylcellulose per 35 mm dish. Colonies (> 50 cells) were scanned after 10 days in culture.

### Subcellular fraction isolation

The nuclear and cytoplasmic fractions were extracted using NE-PER Nuclear and Cytoplasmic Extraction Reagents (Thermo Scientific) according to the manufacturer’s instructions. Total RNA from whole-cell lysates or the nuclear and cytoplasmic fractions were isolated using TRIzol (Life Technologies, Carlsbad, CA). Nucleoli isolation was performed as described with modification^15^. Briefly, 2 × 10^7^ NB4 cells were collected and suspended in 200 μL lysis buffer (10 mM Tris, pH 8.0, 140 mM NaCl, 1.5 mM, MgCl_2_, 0.5% Igepal, add RNAse inhibitor before use) and incubated on ice for 10 min. The lysate was centrifuged at 1000× *g* for 5 min. The supernatant (cytoplasmic extract) was transfered to a clean tube immediately. The insoluble pellet (nuclear extract) was suspended in 200 μL 340 mM sucrose solution containing 5 mM MgCl_2_. To obtain nucleoplasmic and nucleolar fraction, nuclei were broken by sonication until intact nuclei cannot be detected by microscope. 200 μL 880 mM sucrose solution containing 5 mM MgCl_2_ was added gently to the sonicated nuclei and then centrifuged 20 min at 2000× *g*. The supernatant was the nucleoplasmic fraction and the pellet was nucleolar fraction. All centrifugation steps were performed at 4 °C and cell samples and extracts were always kept on ice during experiment. Fractioned RNAs from the same number of cells were used for reverse transcription and qRT-PCR.

### Xenotransplantation model

NOD-SCID mice were maintained under specific pathogen-free conditions in the Laboratory Animal Center of Sun Yat-sen University. All experiments on animals were performed according to the institutional ethical guidelines for animal experiments. RS4;11 cells stably expressing sh-NC, sh-LNC-SNO49AB, PCDH or PCDH-LNC-SNO49AB (GFP^+^ cell populations) were tail vein injected into the mice (5 × 10^6^ cells for LNC-SNO49AB knockdown groups and 3 × 10^6^ cells for overexpressed ones in 150 Μl PBS per mice). Here, PCDH refers to the overexpression vector, PCDH-MSCV-MCS-EF1-Puro-copGFP. For the control, 150 μL of PBS without cells was injected. Thirty days after inoculation, xenografted mice were sacrificed for analysis. Human cell engraftment (GFP^+^ cell populations) in bone marrow was evaluated by flow cytometry^82,83^. The remaining mice were used to perform the survival assay.

### Northern blotting

Biotin-labeled RNA probes were prepared by adding biotin-labeled UTP (Roche) in in vitro transcription using TranscriptAid T7 High Yield Transcription Kit (Thermo Scientific). The PCR primers for RNA probe are listed in Supplementary Table S6. A total of 20 μg RNA run on formaldehyde denaturing agarose gel and transferred to a Hybond-N^+^ membrane (GE Healthcare) capillary transfer. Membranes were dried and ultraviolet-crosslinked. Pre-hybridization was done at 42 °C for 1 h and hybridization was performed at 42 °C overnight.

### Measurement of LNC-SNO49AB copy number

A serial dilution of the plasmin Blunt-LNC-SNO49AB was used in qPCR to generate a standard curve. To measure the copy number of LNC-SNO49AB in leukemia cells, total RNA extracted from 1 × 10^6^ cells of each line was reverse transcribed into cDNAs for qPCR analysis and the copy number was quantitated from the standard curve.

### Protein recombination and purification

Protein recombination and purification were performed as described previously^22,38^. Briefly, recombinant proteins were expressed in *E. coli* strain BL21 [Transetta (DE3) chemically competent cell (Transgen biotech, CD801)]. In brief, 5 μL Luria-Bertani (LB) culture supplemented with 100 μg/μL ampicillin was inoculated with a single colony at 37 °C. After overnight growth, the culture was diluted 100-fold into 300 mL LB supplemented with 100 μg/mL ampicillin. Protein expression was induced in the presence of 0.4 mM IPTG at 16 °C overnight. Then the cell pellets were collected by centrifugation at 5000 rpm, 4 °C for 10 min and purified recombinant proteins using a His or Flag tag Protein Purification Kit (BeaverBeads™) according to the manufacturer’s instruction.

### RNA Immunoprecipitation (RIP)

In the RIP experiment, anti-FLAG, anti-HA, or anti-IgG antibodies were used along with an EZ-Magna RIP™ RNA-Binding Protein Immunoprecipitation Kit (17–701) (Merck Millipore, Germany) according to the manufacturer’s instructions. In HA-tagged Flag-ADAR1 fragments RIP assays, 1 × 10^6^ 293T cells were transfected into 5 μg indicated plasmid and collected after 48 h. All proteins for RIP were lysed with cell lysis buffer supplemented with Thermo Scientific™ Halt™ Protease Inhibitor Cocktail (Thermo Fisher, USA). To prepare antibody-coated beads, 50 μL Protein A/G magnetic beads were incubated with 3 μg antibody or control IgG in 500 μL wash buffer at 4 °C for 1 h. Then the beads were washed three times and mixed with the cell lysates in new tubes. The tubes were rotated at 4 °C overnight. Finally, RNA extraction from the beads was further collected by using Trizol according to the manufacturer’s instructions. Reverse transcription and qPCR were performed as previously described.

### Trsa RNA pull-down assay

The LNC-SNO49AB sequence was cloned into the BLUNT plasmid with the tRSA tag at its 5′ end. RNA products were transcribed in vitro using the TranscriptAid T7 High Yield Transcription Kit (Thermo, USA) and were then purified using the GeneJET RNA Purification Kit (Thermo, USA). The RNA pull-down assay was performed using 50 pmol RNA for each sample with the manufacturer’s instructions of Pierce Magnetic RNA-Protein Pull-down Kit (Thermo, USA). Briefly, folded tRSA and tRSA-labeled LNC-SNO49AB were mixed with RS4;11 cell extract (containing 2 mg total protein) in 400 µL RIP buffer and incubated at RT (room temperature) for one hour. Next, 50 µL of washed streptavidin magnetic beads was added to each reaction and further incubated at RT for another hour. Beads were washed briefly with wash buffer six times and then boiled in SDS loading buffer. Finally, the enriched proteins were resolved via SDS-PAGE and silver stained followed by mass spectrometry (MS) identification (FitGene Biotechnology, China) and western blot.

### In vitro RNA pull-down

The 50 pmol tRSA-RNA products were first mixed with 1–3 μg recombinant proteins, and then the mixture was rotated at 4 °C for 1 h in RNA binding buffer according to Pierce Magnetic RNA-Protein Pull-down Kit. Beads were washed three times with RNA wash buffer and then boiled in SDS loading buffer. Finally, the enriched proteins were resolved via SDS-PAGE and analyzed western blotting.

### Immunoblotting

Total protein was extracted from cells using RIPA lysis buffer (Beyotime, China) with 1× complete ULTRA (Roche, USA). Proteins were resolved by 10% or 12% BisTrispolyacrylamide gels and then transferred to polyvinylidene fluoride membranes. Membranes were blocked in 5% BSA for 1 h, and followed by the appropriate antibody overnight at 4 °C and then incubated with horseradish peroxidase-conjugated secondary antibodies at room temperature for 1 h. Membranes were visualized with an enhanced chemoluminescence detection system.

### RNA sequencing and A-to-I editing level

The si-NC, si-LNC-SNO49AN, and si-ADAR1 samples (5 × 10^6^ cells/sample) were obtained from RS4;11 cells. The next-generation sequencing in this study was performed by poly-A RNA-seq on an illumina noveSeq instrument. The sequencing depth of the RNA-Seq is up to 6 G in 150-base paired-end mode. For the data analysis, pair-end reads were adapter and quality trimmed using Trim Galore (v0.6.4), and high-quality reads were aligned to reference transcriptome (Gencode v34) and quantified using Salmon (v1.3.0) with fragment GC bias and positional bias corrections^84^. DESeq2 was used for normalizing gene expression level and identifying differentially expressed genes^85^. Fragments Per Kilobase of exon model per Million mapped fragments (FPKM) were used to quantify the gene expression. False discovery rate (FDR) was used to compare the si-LNC-SNO49AB, si-ADAR1 and si-NC. A-to-I editing analysis was performed as described previously^86^. Briefly, we collected all mismatches between the aligned reads and the reference genome, discarding mismatches in read positions with quality Phred score < 30 and those located at sites reported as genomic SNPs in dbSNP (SNP build 135)^87^.

### 2-OMe-seq

2-OMe-seq was carried out as previously described with some modifications^88^. Briefly, for each experiment, 1 μg of total RNA were reverse transcribed using either 1 mM final dNTP (high dNTP sample), or 0.004 mM final dNTP (low dNTP sample), 1 U/μL AMV Reverse Transcriptase (NEB) and random primers. Template RNA was degraded by the addition of 1 μL RNase H (BioLegend) and 1 μL of RNase A (NEB). Following purification using VAHTSTM DNA Beads (Vazyme), cDNAs were converted into a dedicated library by VAHTS ssDNA library prep kit for Illumina (Vazyme). This approach allowed us to keep the strand-specificity of the library, so that each read started 1 nt downstream of the RT stopping point. Libraries were subjected to sequencing on the Illumina™ NextSeq 500 Sequencer. Then, we aligned the sequence data to GRCh38.p12 genome by bowtie2 v2.4.5 (default parameter), and normalized each same treatment sample by mapped reads count. Then, we made 5 bp windows by bedtools v2.30.0 and summed the total counts in the window. Finally, we calculate 2-OMe ratio following the previous method^88^.

### Validations by Sanger sequencing

To validate whether the identified editing sites are bona fide we performed regular PCR to amplify a selection of sites. We used SYBR Premix ExTaq real-time PCR Kit (Takara, Japan) for the PCR reactions. All Sanger sequencing was carried out by RuiBiotech (Beijing, China).

### Statistical analysis

Mann-Whitney test was used to analyze the LNC-SNO49AB level between patients with or without leukemia. Kruskal-Wallis test was performed to compare multiple patient groups, and the Dunn’s multiple comparisons test was used to analyze multiple comparisons. Fisher’s exact test was used to determine the significance of differentially expressed lncRNA and mRNA levels between two groups. Data are expressed as the means ± SEM or ±SD of three independent experiments. Kaplan–Meier method with a log-rank test was used to analyze the mice survival. Two-tailed tests were used for univariate comparisons. Throughout the text: ns, not significant; ^∗^*P* < 0.05; ^∗∗^*P* < 0.01; ^∗∗∗^*P* < 0.001.

## Supplementary information

Supplemental Fig S1

Supplemental Fig S2

Supplemental Fig S3

Supplemental Fig S4

Supplemental Fig S5

Supplemental Fig S6

Supplemental Fig S7

Supplemental Tab S1

Supplemental Tab S2

Supplemental Tab S3

Supplemental Tab S4

Supplemental Tab S5

Supplemental Tab S6
