# Supplementary material for: The snoRNA-like lncRNA LNC-SNO49AB drives leukemia by activating the RNA-editing enzyme ADAR1
Source: Cell Discov. 2022 Nov 1;8:117. doi: 10.1038/s41421-022-00460-9 (PMC9622897; doi:10.1038/s41421-022-00460-9)
Supplement: Supplementary file 1 — Supplemental Fig S1 [file 41421_2022_460_MOESM1_ESM.pdf]

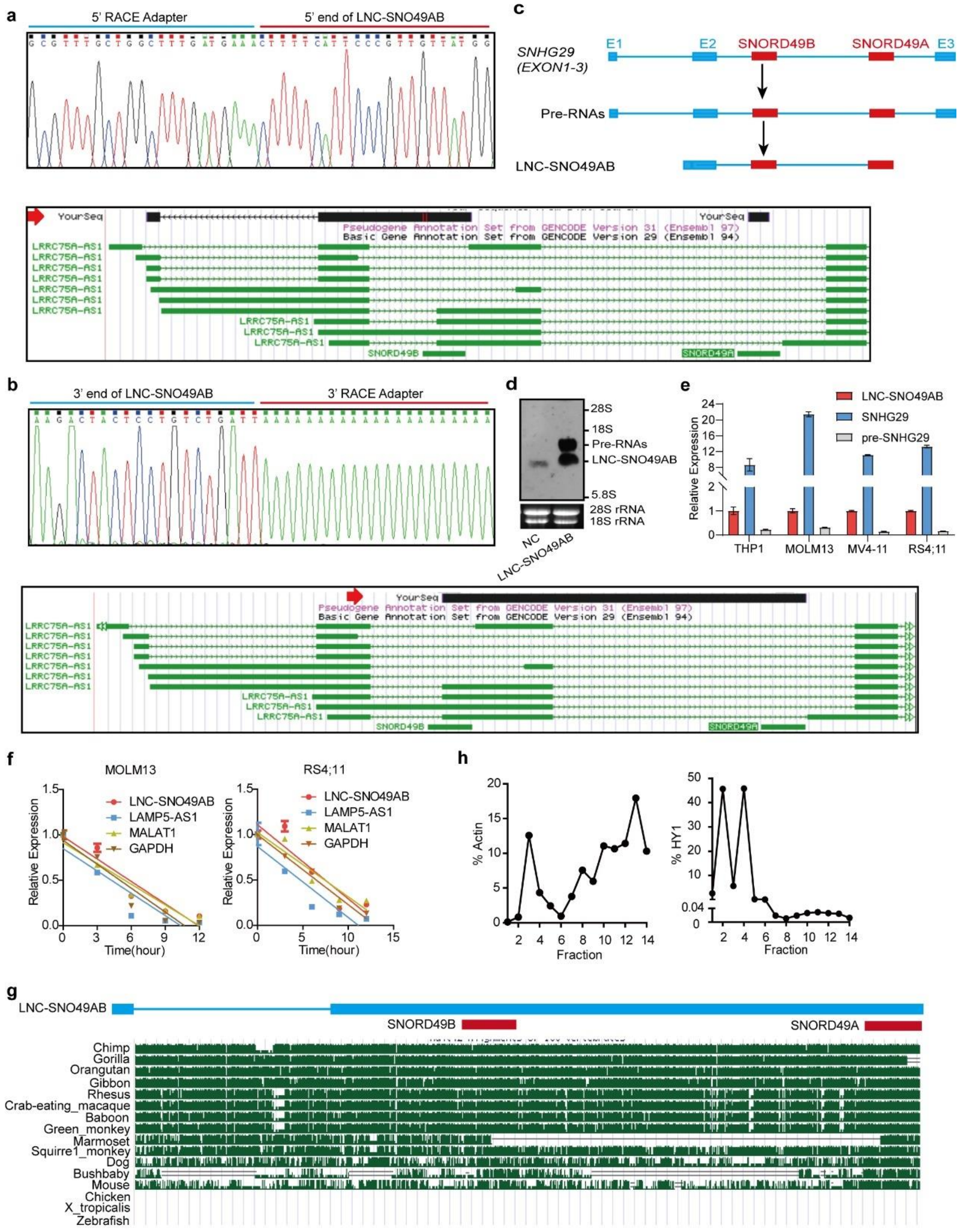

Supplementary Fig. S1 The full-length, conservation and coding potential of LNC-SNO49AB. a, **b** Sanger sequencing of the 5' (**a**, upper) and 3' ends (**b**, upper) of LNC-SNO49AB and the genome browser depiction of the related sequence in UCSC (**a**, **b**, below). we have rephrased SNORD49A/B host gene as SNHG29 (GENCODE

Version 39) instead of LRRC75A-AS1 (GENCODE Version 29). **c** Schematic of the strategy to overexpress LNC-SNO49AB. **d** Northern blot analysis confirmed the overexpression of LNC-SNO49AB. **e** The expression level of LNC-SNO49AB, SNHG29 and pre-SNHG29 in THP1, MOLM13， MV4-11 and RS4;11, as measured by qRT-PCR. **f** The abundance of LNC-SNO49AB, MALAT1 and GAPDH in MOLM13 and MV4-11 cells treated with actinomycin D at the indicated time points. **g** Multiz alignment of LNC-SNO49AB in multiple vertebrate species depicted as well as the per base phastCons conservation score. **h** Sucrose sedimentation analysis of RNA levels from each fraction of MOLM13 cells as measured by RT-qPCR. Relative levels of ACTB (mRNA) and HY1 (noncoding RNA) are shown for each collected fraction.
