# Supplementary material for: The snoRNA-like lncRNA LNC-SNO49AB drives leukemia by activating the RNA-editing enzyme ADAR1
Source: Cell Discov. 2022 Nov 1;8:117. doi: 10.1038/s41421-022-00460-9 (PMC9622897; doi:10.1038/s41421-022-00460-9)
Supplement: Supplementary file 2 — Supplemental Fig S2 [file 41421_2022_460_MOESM2_ESM.pdf]

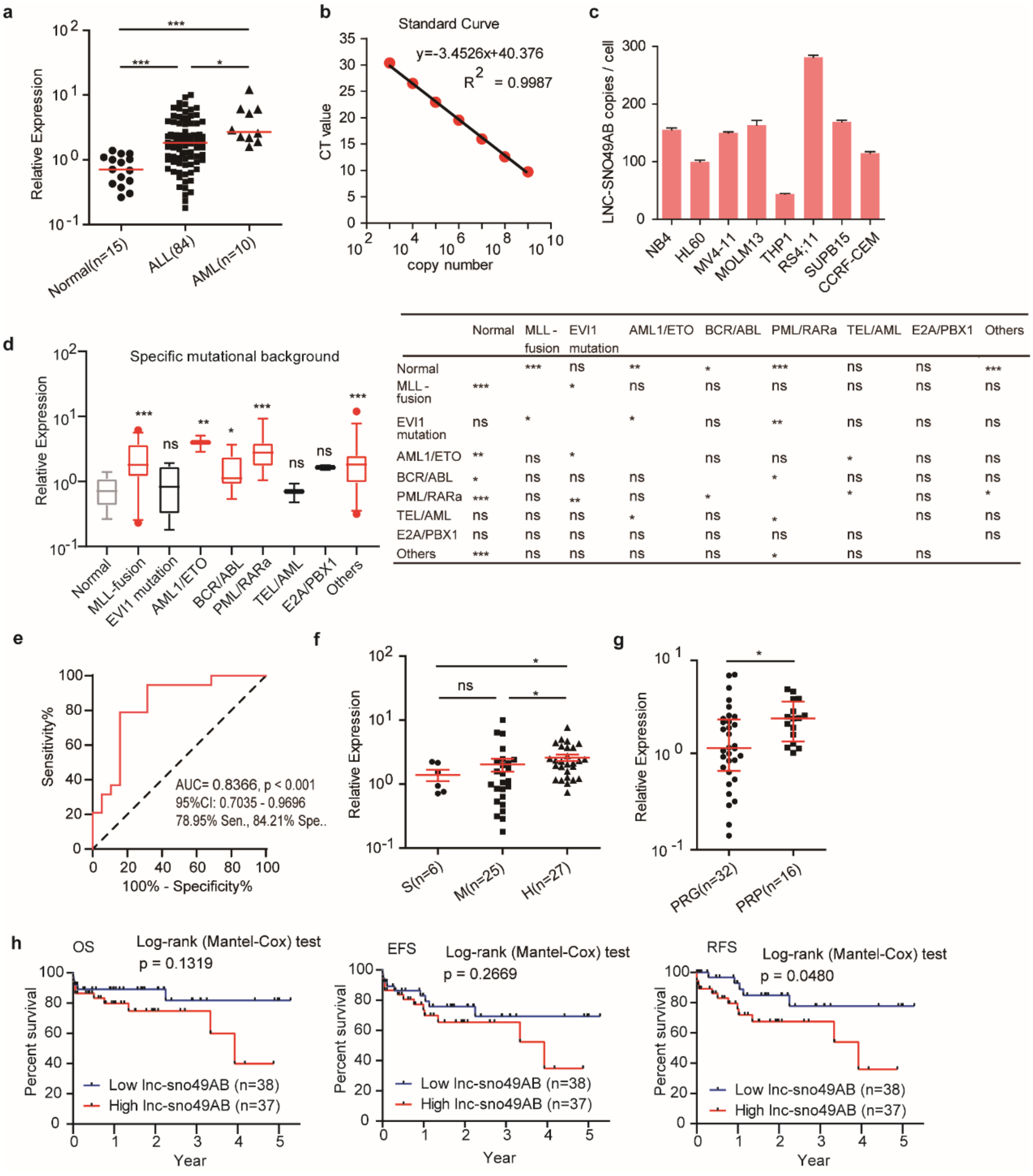

	Normal	MLL-fusion	EVI1 mutation	AML1/ETO	BCR/ABL	PML/RARa	TEL/AML	E2A/PBX1	Others
Normal		***	ns	**	*	***	ns	ns	***
MLL-fusion	***		*	ns	ns	ns	ns	ns	ns
EVI1 mutation	ns	*		*	ns	**	ns	ns	ns
AML1/ETO	**	ns	*		ns	ns	*	ns	ns
BCR/ABL	*	ns	ns	ns		*	ns	ns	ns
PML/RARa	***	ns	**	ns	*		*	ns	*
TEL/AML	ns	ns	ns	*	ns	*		ns	ns
E2A/PBX1	ns	ns	ns	ns	ns	ns	ns		ns
Others	***	ns	ns	ns	ns	*	ns	ns	

Supplementary Fig. S2 LNC-SNO49AB is highly expressed in leukemia patient samples and could be a predictor of poor outcomes. a The relative expression of LNC-SNO49AB in acute lymphoblastic leukemia (ALL) and acute myeloid leukemia (AML). *$p < 0.05$, and ***$p < 0.001$. Kruskal-Wallis test and the Dunn's multiple comparisons test were used. **b** Generation of a standard curve for calculating LNC-SNO49AB copy number. The resultant CT values decreased linearly with increasing LNC-SNO49AB copy number, indicating sensitive detection from a wide range of template amounts. **c** Absolute quantification of LNC-SNO49AB transcript copy number per cell in various leukemia cell lines. **d** The expression level of LNC-SNO49AB

in diverse leukemia subtypes with different mutational background. Left panel showing the comparison of LNC-SNO49AB expression levels between specific mutational backgrounds and normal samples. Right panel showing the multiple comparisons analysis of LNC-SNO49AB expression among these specific mutational backgrounds and normal. Kruskal-Wallis test and Dunn's test has been used. ***, p<0.001, **, p<0.01, *, p<0.05, ns, not significant. **e** ROC curve analysis showed that LNC-SNO49AB had high AUC values of 0.8366 (95% confidence interval (CI) 0.7035–0.9696, p< 0.001) with with 78.95% sensitivity (sen.) and 84.21% specificity (spe.) at the optimal cutoff point calculated by likelihood ratio. **f** LNC-SNO49AB expression in different risk groups for ALL was confirmed using qRT-PCR. Gene expression was normalized to *GAPDH* mRNA. Patients were clustered into standard risk (S), middle risk (M) and high risk (H) groups according to the risk stratification system from ALLIC BFM 2002. *p < 0.05, ns, not significant. Kruskal-Wallis test and the Dunn's multiple comparisons test were used. **g** Differently expressed LNC-SNO49AB in ALL patients with varying responses to prednisone treatment. Gene expression was normalized to *GAPDH* mRNA. PGR, prednisone good response. PPR, prednisone poor response. *p < 0.05 by Mann Whitney test. **h** Overall survival (OS) (p = 0.1319), event-free survival (EFS) (p = 0.2669) and recurrence-free survival (RFS) (p = 0.0480) were analysed by Kaplan-Meier method with a log-rank test between LNC-SNO49AB high (n=37) and LNC-SNO49AB low (n=38) leukemia patients.
