# Supplementary material for: The snoRNA-like lncRNA LNC-SNO49AB drives leukemia by activating the RNA-editing enzyme ADAR1
Source: Cell Discov. 2022 Nov 1;8:117. doi: 10.1038/s41421-022-00460-9 (PMC9622897; doi:10.1038/s41421-022-00460-9)
Supplement: Supplementary file 3 — Supplemental Fig S3 [file 41421_2022_460_MOESM3_ESM.pdf]

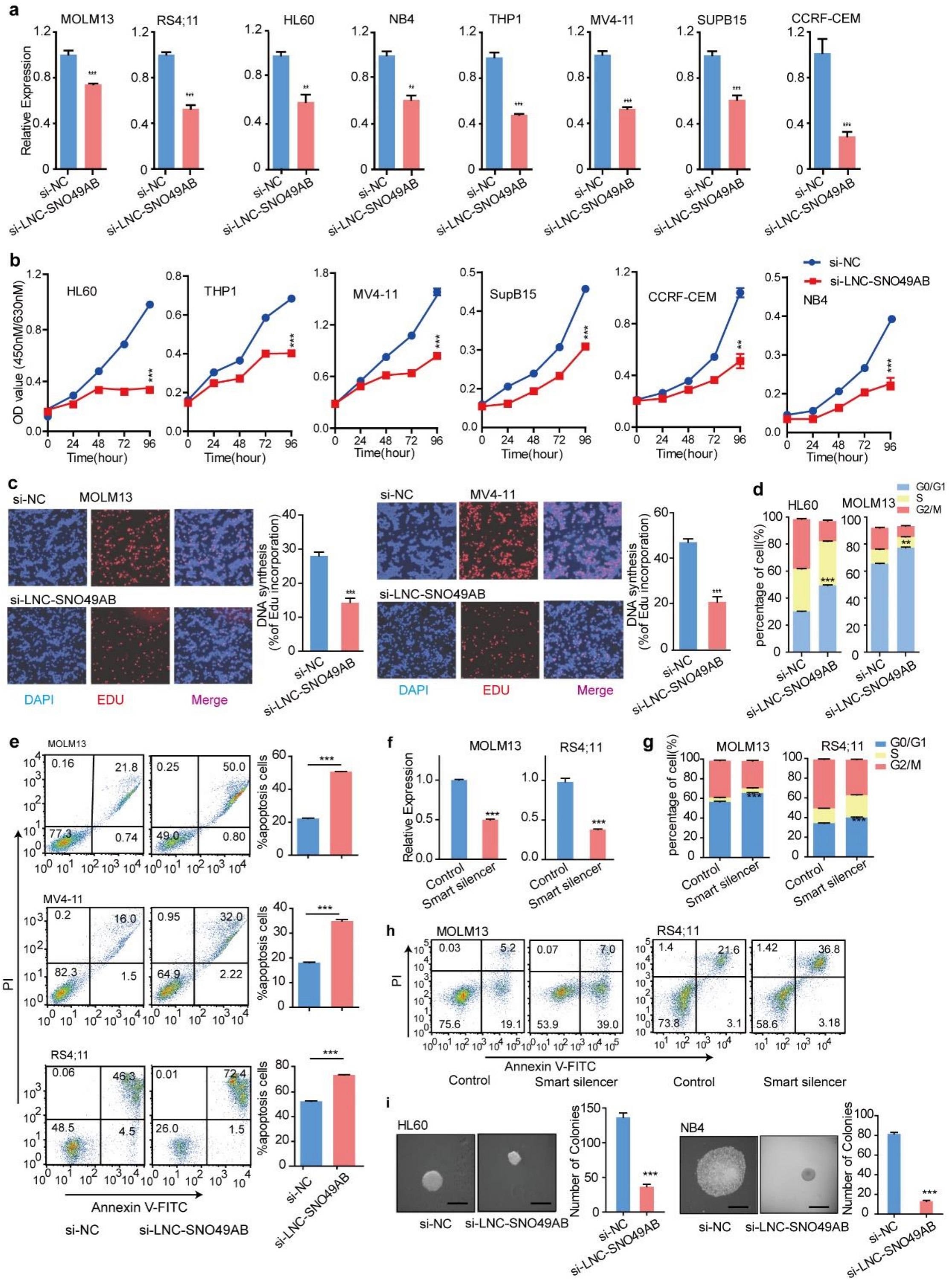

Supplementary Fig. S3 The expression pattern of LNC-SNO49AB in leukemia and its oncogenic role *in vitro*. a qRT-PCR confirmation of LNC-SNO49AB knockdown by siRNAs in various leukemia cell lines. Gene expression was normalized to *GAPDH* mRNA. Values are the mean ± SEM of three independent experiments. $**p < 0.01$, and $***p < 0.001$ by Student's t test. **b** Effects of knocking down LNC-SNO49AB

expression on the proliferation of multiple leukemia cell lines as determined by CCK-8 assay. Values are the mean ± SEM of three independent experiments. **$p < 0.01$, and ***$p < 0.001$ by Student's t test. **c** DNA synthesis was assessed using an EdU (5-ethynyl-2'-deoxyuridine) assay of MOLM13 and MV4-11 cells transfected with si-NC or si-LNC-SNO49AB for 48 h. The cells were fluorescently stained with EdU (red). Nuclei were stained with DAPI (blue). Micrographs represent at least three experiments. Values are the mean ± SEM of three independent experiments. ***$p < 0.001$ by Student's t test. **d** Effects of knocking down LNC-SNO49AB expression on the cell cycle. Values are the mean ± SEM of three independent experiments. **$p < 0.01$, and ***$p < 0.001$ by Student's t test. **e** Effects of knocking down LNC-SNO49AB expression on the 2μM ATO apoptosis of RS4;11, MV4-11 and MOLM13 cells. Values are the mean ± SEM of three independent experiments. ***$p < 0.001$ by Student's t test. **f** qRT-PCR confirmation of LNC-SNO49AB knockdown by smart silencing in RS4;11 and MOLM13 cells. Values are the mean ± SEM of three independent experiments. ***$p < 0.001$ by Student's t test. **g, h** Effects of knocking down LNC-SNO49AB expression on the cell cycle(**g**) and 2μM ATO apoptosis(**h**) of RS4;11 and MOLM13 cells. Values are the mean ± SEM of three independent experiments. ***$p < 0.001$ by Student's t test. **i** Colony number formed by si-NC or si-LNC-SNO49AB cells. Scale bars, 100 μm. Values are the mean ± SEM of three independent experiments. ***$p < 0.001$ by Student's t test.
