# Supplementary material for: The snoRNA-like lncRNA LNC-SNO49AB drives leukemia by activating the RNA-editing enzyme ADAR1
Source: Cell Discov. 2022 Nov 1;8:117. doi: 10.1038/s41421-022-00460-9 (PMC9622897; doi:10.1038/s41421-022-00460-9)
Supplement: Supplementary file 4 — Supplemental Fig S4 [file 41421_2022_460_MOESM4_ESM.pdf]

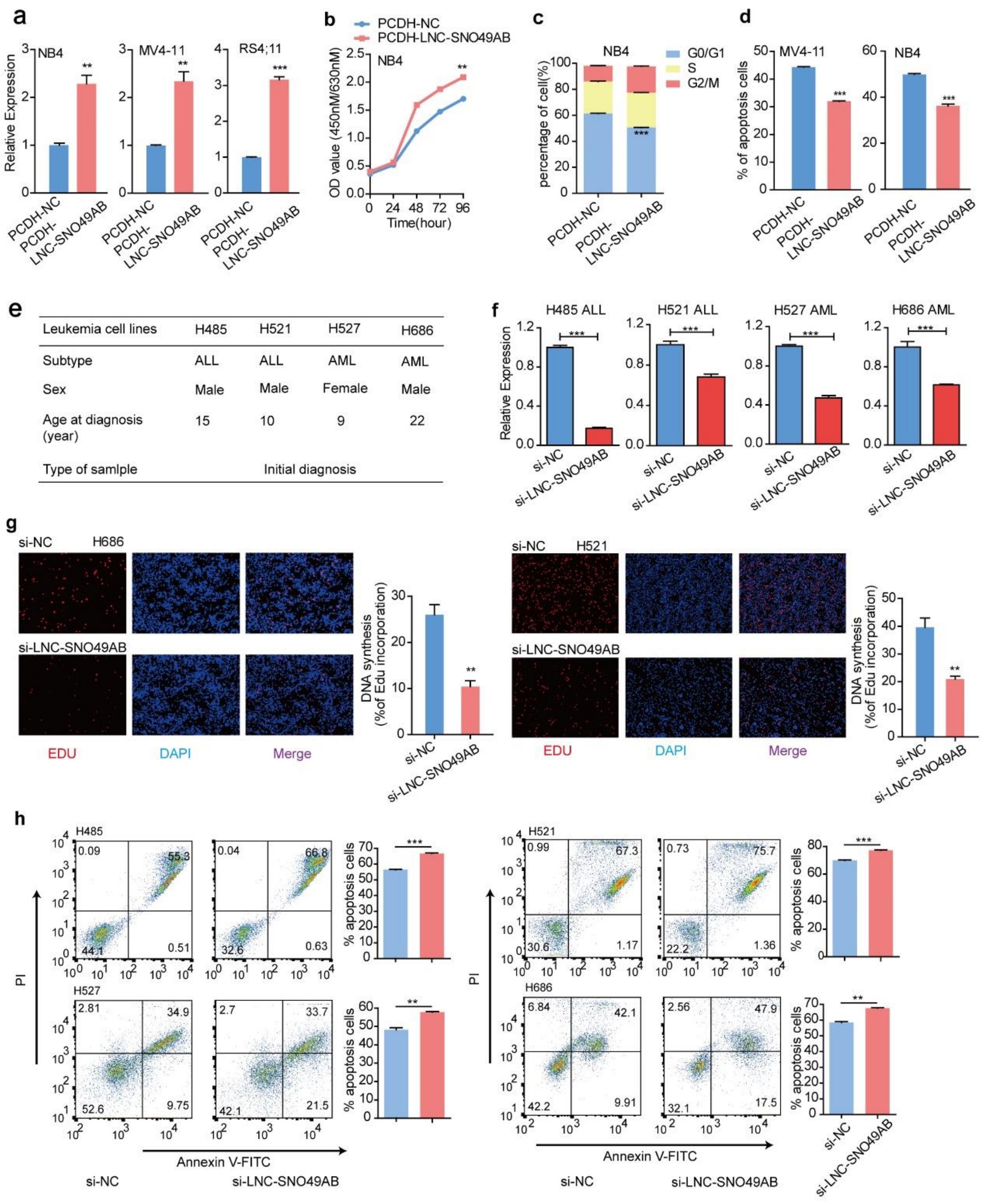

Supplementary Fig. S4 Overexpression and silencing of LNC-SNO49AB influence the growth of leukeamia cells. a qRT-PCR confirmation of forced expression of LNC-SNO49AB in NB4, RS4;11 and MV4-11 cells. Values are the mean ± SEM of three independent experiments. **p < 0.01, and ***p < 0.001 by Student's t test. **b-d** Effects of forced expression of LNC-SNO49AB of cell proliferation (**b**), arrest of the cell cycle (**c**) and 2μM ATO-induced apoptosis (**d**). Values are the mean ± SEM of three independent experiments. **p < 0.01, and ***p < 0.001 by Student's t test. **e** A list of the characteristics of the leukemia patients whose bone marrow samples were used in

this study. **f** qRT-PCR analysis of LNC-SNO49AB expression in primary leukemia cells transfected with si-NC or si-LNC-SNO49AB. Values are the mean ± SEM of three independent experiments. ***$p < 0.001$ by Student's t test. **g, h**. Effects of knocking down LNC-SNO49AB expression on DNA synthesis (**g**) and cell apoptosis (**h**). Values are the mean ± SEM of three independent experiments. **$p < 0.01$, and ***$p < 0.001$ by Student's t test.
