# Supplementary material for: The snoRNA-like lncRNA LNC-SNO49AB drives leukemia by activating the RNA-editing enzyme ADAR1
Source: Cell Discov. 2022 Nov 1;8:117. doi: 10.1038/s41421-022-00460-9 (PMC9622897; doi:10.1038/s41421-022-00460-9)
Supplement: Supplementary file 5 — Supplemental Fig S5 [file 41421_2022_460_MOESM5_ESM.pdf]

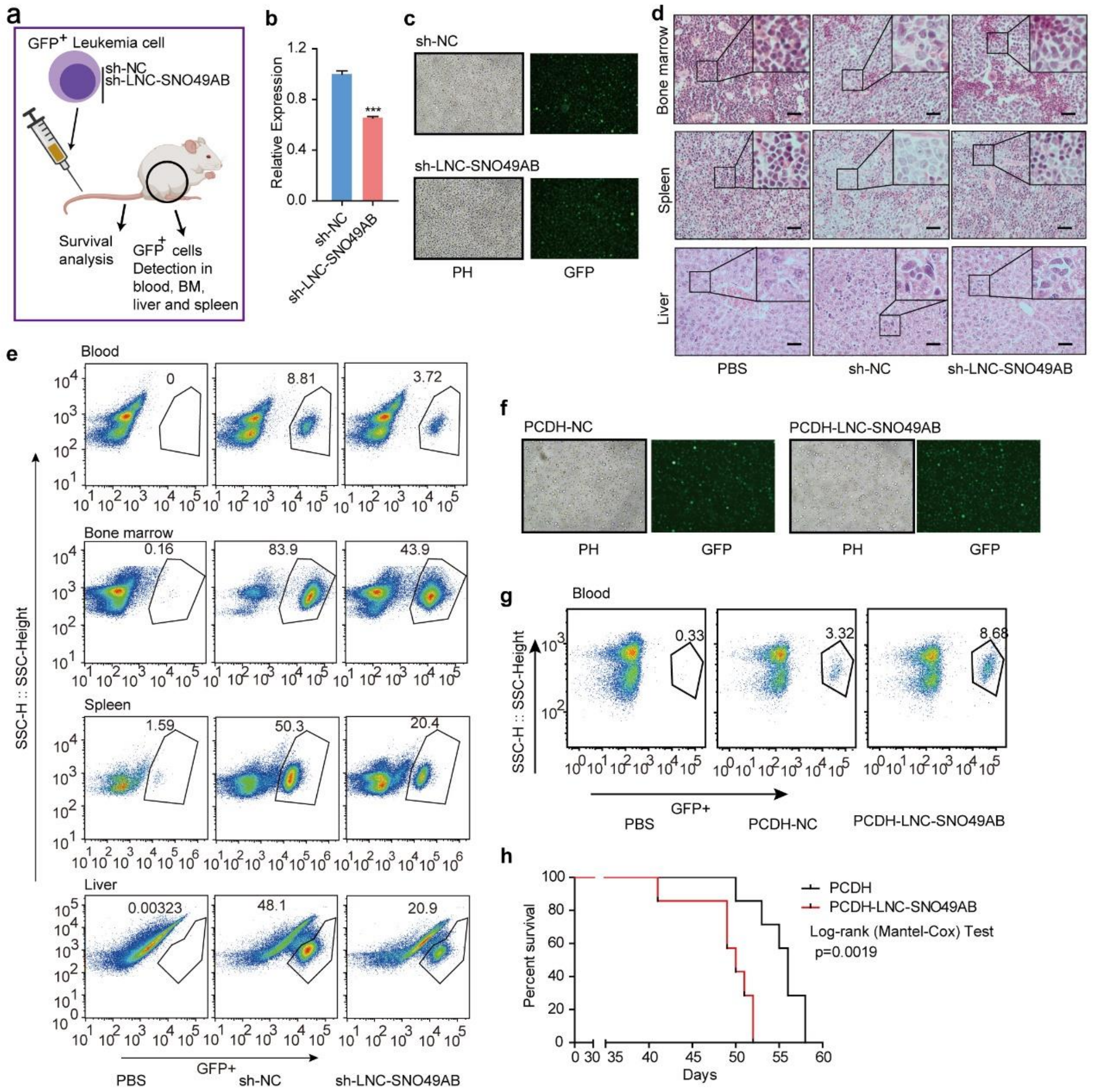

Supplementary Fig. S5 LNC-SNO49AB promotes leukemia progression *in vivo*.

a Schematic representation of the xenotransplantation model. **b**, **c** qRT-PCR (**b**) and fluorescence photography (**c**) confirmation of the establishment of GFP+ stable knockdown LNC-SNO49AB cells and control cells. Gene expression was normalized to *GAPDH* mRNA. Values are the mean ± SEM of three independent experiments. ***p

< 0.001 by Student's t test. **d** Haematoxylin and eosin (H&E) staining of the bone marrow (BM), spleen and liver of recipient mice. The magnified images showed the infiltrated leukemia cells. Scale bars, 50 μm. **e** Representative flow cytometry plot showing GFP+ leukemia cells in peripheral blood, BM, spleen and liver of the mice treated with PBS, sh-NC cells and sh-LNC-SNO49AB cells. **f** Fluorescence photography confirmation of the establishment of LNC-SNO49AB overexpression cells stably overexpressing GFP+ and control cells. **g** Representative flow cytometry plot showing GFP+ leukemia cells in the peripheral blood of the mice treated with PBS, PCDH-NC cells or PCDH-LNC-SNO49AB cells. **h** Kaplan-Meier survival curves of NOD-SCID mice transplanted with PCDH-NC and PCDH-LNC-SNO49AB-treated RS4;11 cells (n=7 mice per group). P-values were calculated by log-rank (Mantel-Cox) test.
