# Supplementary material for: The snoRNA-like lncRNA LNC-SNO49AB drives leukemia by activating the RNA-editing enzyme ADAR1
Source: Cell Discov. 2022 Nov 1;8:117. doi: 10.1038/s41421-022-00460-9 (PMC9622897; doi:10.1038/s41421-022-00460-9)
Supplement: Supplementary file 6 — Supplemental Fig S6 [file 41421_2022_460_MOESM6_ESM.pdf]

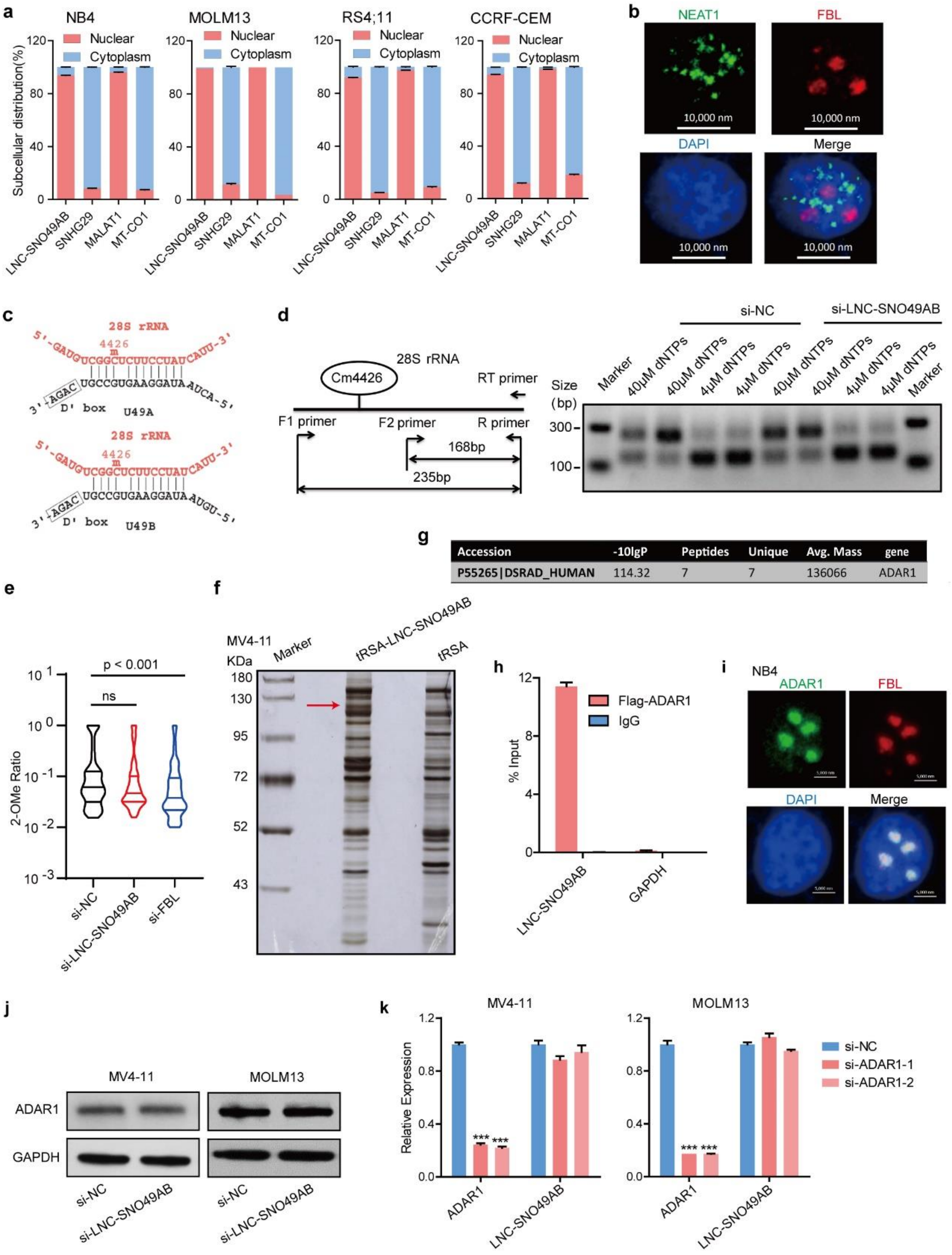

Supplementary Fig. S6 The effects of LNC-SNO49AB on 2'-O-methylation and the expression of ADAR1. a Subcellular localization of LNC-SNO49AB and SNHG29 RNAs in NB4, MOLM13, RS4;11 and CCRF-CEM cells. MALAT1 and MT-CO1 RNAs were used as controls for nuclear and cytoplasmic fractions, respectively. **b** Representative images of dcas13-mediated NEAT1 labelling (green); NEAT1 was not colocalized with FBL (red) in NB4 cells. Scale bars, 10 μm. **c** SNORD49A and

SNORD49B mediate the 2’-O-methylation of 28S rRNA cytidine4426. **d** Schematic of the primer extension strategy to detect 2’-O-methylation on 28S rRNA cytidine4426. LNC-SNO49AB did not alter the 2’-O-methylation on 28S rRNA cytidine4426. **e** Violin plot of global 2-O-methylation abundance (2-OMe-seq) on rRNA measured by 2-OMe Ratio in si-NC and si-LNC-SNO49AB group, si-FBL as the positive control. Significance is given by Student’s t test. ns, not significant, ***$p < 0.001$. **f** Silver staining of proteins pulled down by LNC-SNO49AB in MV4-11 cells. Red arrow indicates the specific band. **g** ADAR1 protein identified in LC-MS. **h** qRT-PCR measurement of LNC-SNO49AB in an RNA immunoprecipitation (RIP) assay using a Flag antibody. IgG was the negative control, and GAPDH as a specific control. Representative results are shown from three replicates. **i** Cellular localization of endogenous ADAR1 as determined by immunofluorescence. Scale bars, 5 μm. **j** Relative expression of ADAR1 when LNC-SNO49AB was silenced, as detected by western blot analysis. **k** Relative expression of LNC-SNO49AB when ADAR1 was silenced, as detected by qRT-PCR. Values are the mean ± SEM of three independent experiments. ***$p < 0.001$ by Student’s t test.
