# Supplementary material for: The snoRNA-like lncRNA LNC-SNO49AB drives leukemia by activating the RNA-editing enzyme ADAR1
Source: Cell Discov. 2022 Nov 1;8:117. doi: 10.1038/s41421-022-00460-9 (PMC9622897; doi:10.1038/s41421-022-00460-9)
Supplement: Supplementary file 7 — Supplemental Fig S7 [file 41421_2022_460_MOESM7_ESM.pdf]

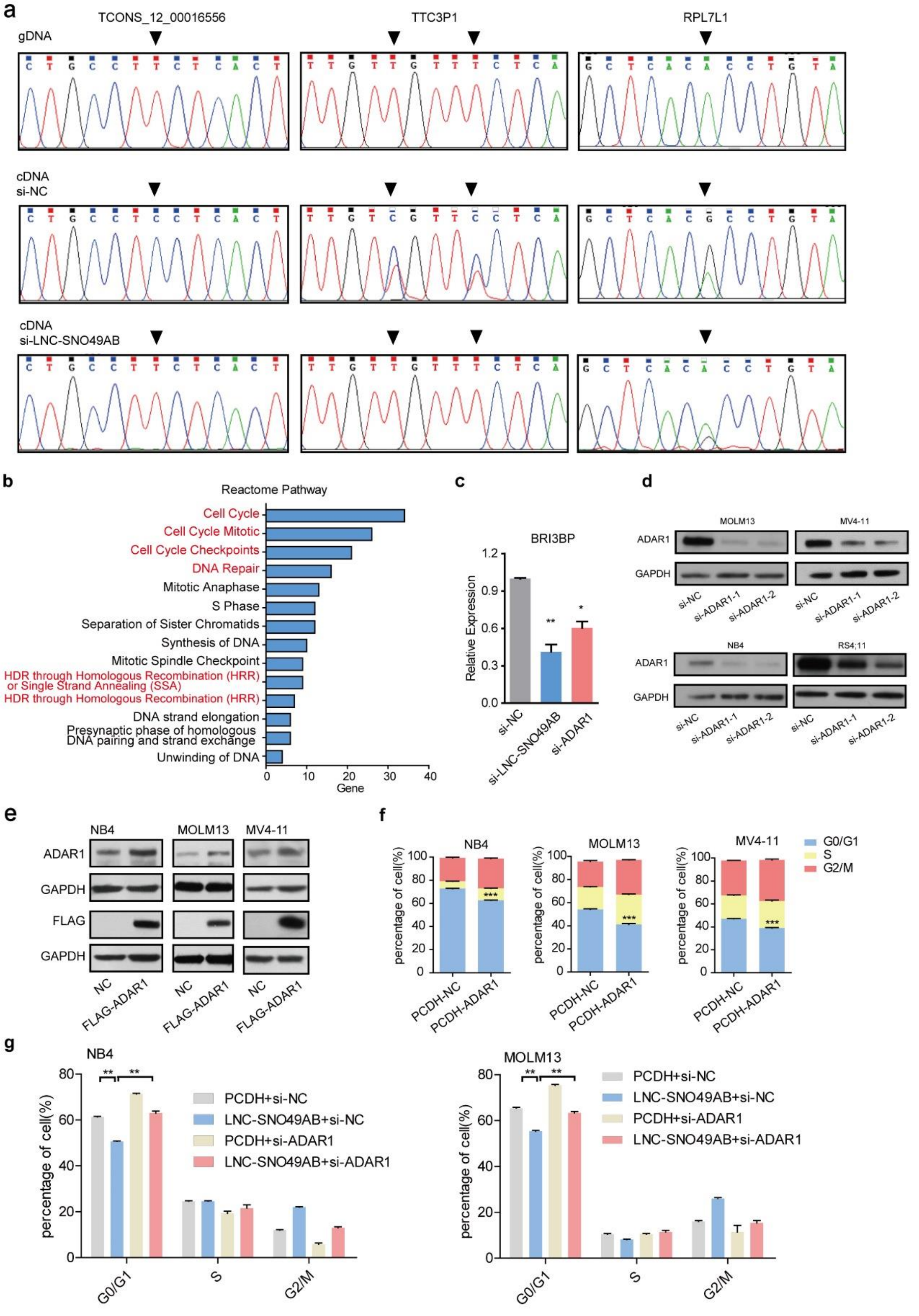

Supplementary Fig. S7 LNC-SNO49AB regulates RNA A-to-I editing in leukemia. **a** Sequence chromatograms of the TCONS_12_00016556, TTC3P1 and RPL7 L1 transcripts in the indicated cell lines; the arrowheads indicate the edited positions. **b** Gene ontology analysis of genes with dysregulated editing rates. **c** Relative expression

of BRI3BP when LNC-SNO49AB or ADAR1 was silenced as determined by qRT-PCR. Values are the mean ± SEM of three independent experiments. *$p < 0.05$, and **$p < 0.01$ by Student's t test. **d**, **e** Western blot confirming the knockdown (**d**) or overexpression (**e**) of ADAR1 in in RS4;11. **f** Effects of overexpression of ADAR1 expression on the cell cycle. Values are the mean ± SEM of three independent experiments. ***$p < 0.001$ by Student's t test. **g** Effects of LNC-SNO49AB overexpression and ADAR1 knockdown on cell cycle regulation in NB4 and MOLM13 cells. Values are the mean ± SEM of three independent experiments. **$p < 0.01$ by Student's t test.
