# Supplementary material for: The snoRNA-like lncRNA LNC-SNO49AB drives leukemia by activating the RNA-editing enzyme ADAR1
Source: Cell Discov. 2022 Nov 1;8:117. doi: 10.1038/s41421-022-00460-9 (PMC9622897; doi:10.1038/s41421-022-00460-9)
Supplement: Supplementary file 8 — Supplemental Tab S1 [file 41421_2022_460_MOESM8_ESM.pdf]

Supplementary Table S1. Characteristics of test cohort.

Type of sample	Characteristics	Median (range)	No.(%)
Initial diagnosed	**Age at diagnosis,years**	5(0.3-13)	
(N=94)	**Sex**		
	Male		50(53.2)
	Female		32(34.0)
	N/A		12(12.8)
	**ALL**		84(89.4)
	**AML**		10(10.6)
	**Immunophenotype**		
	B		41(48.8)
	T		20(23.8)
	N/A		23(27.4)
	**Mutational background**		
	MLL-fusion		27(28.7)
	AML1/ETO		2(2.1)
	PML/RARa		13(13.8)
	E2A/PBX1		2(2.1)
	EVI1 mutation		5(5.3)
	BCR/ABL		9(9.6)
	TEL/AML		2(2.1)
	Other		34(36.2)
	**Risk group**		
	SR		6(7.1)
	MR		25(29.8)
	HR		27(32.1)
	N/A		26(31.0)
	**Prednisone response**		
	Good response		32(34.0)
	Poor response		16(17.0)
	N/A		46(49.0)
After therapy	CR		19
(N=19)			
Normal			
(N=15)			

N/A : not applicable.
