# Supplementary material for: The snoRNA-like lncRNA LNC-SNO49AB drives leukemia by activating the RNA-editing enzyme ADAR1
Source: Cell Discov. 2022 Nov 1;8:117. doi: 10.1038/s41421-022-00460-9 (PMC9622897; doi:10.1038/s41421-022-00460-9)
Supplement: Supplementary file 9 — Supplemental Tab S2 [file 41421_2022_460_MOESM9_ESM.pdf]

Supplementary Table S2 Proteins (100~130 KDa) identified in specific bands by MS may interact with LNC-SNO49AB.

Name	-10lgP	Peptides	Unique	Avg. Mass(Da)	Description
SND1	230.51	27	27	101997	Staphylococcal nuclease domain-containing protein 1 OS=Homo sapiens GN=SND1 PE=1 SV=1
C1TC	223.78	24	23	101559	C-1-tetrahydrofolate synthase cytoplasmic OS=Homo sapiens GN=MTHFD1 PE=1 SV=3
UBA1	205.93	17	17	117849	Ubiquitin-like modifier-activating enzyme 1 OS=Homo sapiens GN=UBA1 PE=1 SV=3
SYAC	199.77	19	19	106810	Alanine--tRNA ligase cytoplasmic OS=Homo sapiens GN=AARS PE=1 SV=2
NNTM	194.69	21	21	113895	NAD(P) transhydrogenase mitochondrial OS=Homo sapiens GN=NNT PE=1 SV=3
ACLY	190.14	18	18	120839	ATP-citrate synthase OS=Homo sapiens GN=ACLY PE=1 SV=3
XRN2	189.94	17	17	108583	5'-3' exoribonuclease 2 OS=Homo sapiens GN=XRN2 PE=1 SV=1
VINC	185.24	23	23	123799	Vinculin OS=Homo sapiens GN=VCL PE=1 SV=4
ZFR	178.48	14	13	117012	Zinc finger RNA-binding protein OS=Homo sapiens GN=ZFR PE=1 SV=2
DHX9	171.84	16	16	140958	ATP-dependent RNA helicase A OS=Homo sapiens GN=DHX9 PE=1 SV=4
RRP44	163.55	13	13	109003	Exosome complex exonuclease RRP44 OS=Homo sapiens GN=DIS3 PE=1 SV=2
USO1	162.87	14	14	107895	General vesicular transport factor p115 OS=Homo sapiens GN=USO1 PE=1 SV=2
ERAP1	160.42	15	15	107235	Endoplasmic reticulum aminopeptidase 1 OS=Homo sapiens GN=ERAP1 PE=1 SV=3
SK2L2	154.02	15	15	117805	Superkiller viralicidic activity 2-like 2 OS=Homo sapiens GN=SKIV2L2 PE=1 SV=3
XPO2	148.27	13	13	110417	Exportin-2 OS=Homo sapiens GN=CSE1L PE=1 SV=3
DDB1	146.43	18	17	126968	DNA damage-binding protein 1 OS=Homo sapiens GN=DDB1 PE=1 SV=1
U5S1	141.32	11	10	109436	116 kDa U5 small nuclear ribonucleoprotein component OS=Homo sapiens GN=EFTUD2 PE=1 SV=1

PUR2	131.84	9	9	107767	Trifunctional purine biosynthetic protein adenosine-3 OS=Homo sapiens GN=GART PE=1 SV=1
SC24C	128.79	6	6	118325	Protein transport protein Sec24C OS=Homo sapiens GN=SEC24C PE=1 SV=3
RB	126.55	9	9	106159	Retinoblastoma-associated protein OS=Homo sapiens GN=RB1 PE=1 SV=2
EIFCL	125.25	8	8	105473	Eukaryotic translation initiation factor 3 subunit C-like protein OS=Homo sapiens GN=EIF3CL PE=3 SV=1
EIF3C	125.25	8	8	105344	Eukaryotic translation initiation factor 3 subunit C OS=Homo sapiens GN=EIF3C PE=1 SV=1
AT2A2	122.99	8	7	114757	Sarcoplasmic/endoplasmic reticulum calcium ATPase 2 OS=Homo sapiens GN=ATP2A2 PE=1 SV=1
OGT1	121.66	11	11	116924	UDP-N-acetylglucosamine--peptide N-acetylglucosaminyltransferase 110 kDa subunit OS=Homo sapiens GN=OGT PE=1 SV=3
DHX30	118.26	9	9	133938	Putative ATP-dependent RNA helicase DHX30 OS=Homo sapiens GN=DHX30 PE=1 SV=1
KINH	117.83	6	6	109685	Kinesin-1 heavy chain OS=Homo sapiens GN=KIF5B PE=1 SV=1
DSRAD	114.32	7	7	136066	Double-stranded RNA-specific adenosine deaminase OS=Homo sapiens GN=ADAR PE=1 SV=4
UBA6	112.85	9	8	117970	Ubiquitin-like modifier-activating enzyme 6 OS=Homo sapiens GN=UBA6 PE=1 SV=1
IPO5	111.39	8	8	123630	Importin-5 OS=Homo sapiens GN=IPO5 PE=1 SV=4
HXK1	109.77	9	6	102486	Hexokinase-1 OS=Homo sapiens GN=HK1 PE=1 SV=3
HXK2	108.28	8	5	102380	Hexokinase-2 OS=Homo sapiens GN=HK2 PE=1 SV=2
NU107	106.28	7	7	106374	Nuclear pore complex protein Nup107 OS=Homo sapiens GN=NUP107 PE=1 SV=1
PSMD1	105.84	6	6	105836	26S proteasome non-ATPase regulatory subunit 1 OS=Homo sapiens GN=PSMD1 PE=1 SV=2
XPO1	100.2	6	6	123386	Exportin-1 OS=Homo sapiens GN=XPO1 PE=1 SV=1

RBM10	99.52	6	5	103533	RNA-binding protein 10 OS=Homo sapiens GN=RBM10 PE=1 SV=3
IDE	98.38	7	6	117968	Insulin-degrading enzyme OS=Homo sapiens GN=IDE PE=1 SV=4
MOV10	96.61	11	11	113671	Putative helicase MOV-10 OS=Homo sapiens GN=MOV10 PE=1 SV=2
NCKPL	95.57	8	7	128153	Nck-associated protein 1-like OS=Homo sapiens GN=NCKAP1L PE=1 SV=3
CORO7	94.95	4	4	100605	Coronin-7 OS=Homo sapiens GN=CORO7 PE=1 SV=2
PK3CD	94	9	6	119479	Phosphatidylinositol 4 5-bisphosphate 3-kinase catalytic subunit delta isoform OS=Homo sapiens GN=PIK3CD PE=1 SV=2
AP1B1	92.98	9	4	104637	AP-1 complex subunit beta-1 OS=Homo sapiens GN=AP1B1 PE=1 SV=2
PRP6	92.84	7	7	106925	Pre-mRNA-processing factor 6 OS=Homo sapiens GN=PRPF6 PE=1 SV=1
ZCCHV	89.81	5	5	101431	Zinc finger CCCH-type antiviral protein 1 OS=Homo sapiens GN=ZC3HAV1 PE=1 SV=3
PARP1	89.46	6	5	113084	Poly [ADP-ribose] polymerase 1 OS=Homo sapiens GN=PARP1 PE=1 SV=4
DHX36	88.72	7	7	114760	ATP-dependent RNA helicase DHX36 OS=Homo sapiens GN=DHX36 PE=1 SV=2
ARHG2	88.28	7	7	111543	Rho guanine nucleotide exchange factor 2 OS=Homo sapiens GN=ARHGEF2 PE=1 SV=4
RASL3	87.56	5	5	111898	RAS protein activator like-3 OS=Homo sapiens GN=RASAL3 PE=1 SV=2
C1TM	83.62	5	4	105790	Monofunctional C1-tetrahydrofolate synthase mitochondrial OS=Homo sapiens GN=MTHFD1L PE=1 SV=1
UBP11	83.09	3	3	109817	Ubiquitin carboxyl-terminal hydrolase 11 OS=Homo sapiens GN=USP11 PE=1 SV=3
PREP	78.99	6	6	117413	Presequence protease mitochondrial OS=Homo sapiens GN=PITRM1 PE=1 SV=3
RNBP6	76.71	3	3	124713	Ran-binding protein 6 OS=Homo sapiens GN=RANBP6 PE=1 SV=2
AP2B1	72.98	6	1	104553	AP-2 complex subunit beta OS=Homo sapiens GN=AP2B1 PE=1 SV=1

CND3	72.95	3	3	114334	Condensin complex subunit 3 OS=Homo sapiens GN=NCAPG PE=1 SV=1
LONM	71.38	5	5	106489	Lon protease homolog mitochondrial OS=Homo sapiens GN=LONP1 PE=1 SV=2
IPO7	70.68	3	3	119516	Importin-7 OS=Homo sapiens GN=IPO7 PE=1 SV=1
EMAL4	70.41	3	2	108916	Echinoderm microtubule-associated protein-like 4 OS=Homo sapiens GN=EML4 PE=1 SV=3
AT2A1	65.33	3	3	110252	Sarcoplasmic/endoplasmic reticulum calcium ATPase 1 OS=Homo sapiens GN=ATP2A1 PE=1 SV=1
KIF5C	61.02	3	3	109495	Kinesin heavy chain isoform 5C OS=Homo sapiens GN=KIF5C PE=1 SV=1
KIF5A	61.02	2	2	117378	Kinesin heavy chain isoform 5A OS=Homo sapiens GN=KIF5A PE=1 SV=2
NAA25	60.46	4	3	112292	N-alpha-acetyltransferase 25 NatB auxiliary subunit OS=Homo sapiens GN=NAA25 PE=1 SV=1
POTEF	59.69	3	3	121444	POTE ankyrin domain family member F OS=Homo sapiens GN=POTEF PE=1 SV=2
POTEE	59.69	3	3	121363	POTE ankyrin domain family member E OS=Homo sapiens GN=POTEE PE=2 SV=3
POTEI	59.69	3	3	121282	POTE ankyrin domain family member I OS=Homo sapiens GN=POTEI PE=3 SV=1
AP2A1	57.7	3	3	107546	AP-2 complex subunit alpha-1 OS=Homo sapiens GN=AP2A1 PE=1 SV=3
UBA7	57.63	4	4	111694	Ubiquitin-like modifier-activating enzyme 7 OS=Homo sapiens GN=UBA7 PE=1 SV=2
ODO1	56.77	2	2	115935	2-oxoglutarate dehydrogenase mitochondrial OS=Homo sapiens GN=OGDH PE=1 SV=3
SYIM	55.28	4	3	113791	Isoleucine--tRNA ligase mitochondrial OS=Homo sapiens GN=IARS2 PE=1 SV=2
CHD1L	53.8	4	4	100984	Chromodomain-helicase-DNA-binding protein 1-like OS=Homo sapiens GN=CHD1L PE=1 SV=2
POTEJ	51.24	2	2	117390	POTE ankyrin domain family member J OS=Homo sapiens GN=POTEJ PE=3 SV=1

ANKL2	50.61	2	2	104114	Ankyrin repeat and LEM domain-containing protein 2 OS=Homo sapiens GN=ANKLE2 PE=1 SV=4
NEK9	50.34	3	3	107168	Serine/threonine-protein kinase Nek9 OS=Homo sapiens GN=NEK9 PE=1 SV=2
UBP15	50.17	1	1	112419	Ubiquitin carboxyl-terminal hydrolase 15 OS=Homo sapiens GN=USP15 PE=1 SV=3
UBP4	50.17	1	1	108565	Ubiquitin carboxyl-terminal hydrolase 4 OS=Homo sapiens GN=USP4 PE=1 SV=3
AASS	49.2	2	2	102132	Alpha-aminoadipic semialdehyde synthase mitochondrial OS=Homo sapiens GN=AASS PE=1 SV=1
PKN1	48.05	3	2	103932	Serine/threonine-protein kinase N1 OS=Homo sapiens GN=PKN1 PE=1 SV=2
INT3	44	1	1	118070	Integrator complex subunit 3 OS=Homo sapiens GN=INTS3 PE=1 SV=1
GANAB	43.52	3	3	106874	Neutral alpha-glucosidase AB OS=Homo sapiens GN=GANAB PE=1 SV=3
CAND1	42.52	2	2	136375	Cullin-associated NEDD8-dissociated protein 1 OS=Homo sapiens GN=CAND1 PE=1 SV=2
WASC5	40.39	4	4	134286	WASH complex subunit 5 OS=Homo sapiens GN=WASHC5 PE=1 SV=1
WDR6	39.9	2	2	121724	WD repeat-containing protein 6 OS=Homo sapiens GN=WDR6 PE=1 SV=1
OGDHL	36.38	1	1	114481	2-oxoglutarate dehydrogenase-like mitochondrial OS=Homo sapiens GN=OGDHL PE=1 SV=3
PK3CG	35.48	3	3	126454	Phosphatidylinositol 4 5-bisphosphate 3-kinase catalytic subunit gamma isoform OS=Homo sapiens GN=PIK3CG PE=1 SV=3
ARHG1	33.63	2	2	102435	Rho guanine nucleotide exchange factor 1 OS=Homo sapiens GN=ARHGEF1 PE=1 SV=2
IPO11	27.15	1	1	112535	Importin-11 OS=Homo sapiens GN=IPO11 PE=1 SV=1
FA65C	24.82	1	1	105290	Protein FAM65C OS=Homo sapiens GN=FAM65C PE=1 SV=4
RBM15	24.37	1	1	107188	Putative RNA-binding protein 15 OS=Homo sapiens GN=RBM15 PE=1 SV=2
PK3CB	22.48	1	1	122762	Phosphatidylinositol 4 5-bisphosphate 3-kinase catalytic subunit beta isoform

					OS=Homo sapiens GN=PIK3CB PE=1 SV=1
GTF2I	21.82	1	1	112416	General transcription factor II-I OS=Homo sapiens GN=GTF2I PE=1 SV=2
TSH3	21.81	1	1	118566	Teashirt homolog 3 OS=Homo sapiens GN=TSHZ3 PE=1 SV=2
TSH1	21.81	1	1	117916	Teashirt homolog 1 OS=Homo sapiens GN=TSHZ1 PE=1 SV=2
SMG5	21.81	1	1	113928	Protein SMG5 OS=Homo sapiens GN=SMG5 PE=1 SV=3
UBN1	21.45	2	1	121520	Ubinuclein-1 OS=Homo sapiens GN=UBN1 PE=1 SV=2
SRRT	21.39	1	1	100667	Serrate RNA effector molecule homolog OS=Homo sapiens GN=SRRT PE=1 SV=1
SYNE3	21.28	2	2	112216	Nesprin-3 OS=Homo sapiens GN=SYNE3 PE=1 SV=2
SRBD1	21.23	3	2	111776	S1 RNA-binding domain-containing protein 1 OS=Homo sapiens GN=SRBD1 PE=1 SV=2
HERC3	20.92	1	1	117188	Probable E3 ubiquitin-protein ligase HERC3 OS=Homo sapiens GN=HERC3 PE=1 SV=1
RENT1	20.4	1	1	124345	Regulator of nonsense transcripts 1 OS=Homo sapiens GN=UPF1 PE=1 SV=2
SC31A	20.14	1	1	133015	Protein transport protein Sec31A OS=Homo sapiens GN=SEC31A PE=1 SV=3
CE112	19.03	1	1	112749	Centrosomal protein of 112 kDa OS=Homo sapiens GN=CEP112 PE=1 SV=2
XPO4	18.45	1	1	130139	Exportin-4 OS=Homo sapiens GN=XPO4 PE=1 SV=2
ITAX	17.99	1	1	127829	Integrin alpha-X OS=Homo sapiens GN=ITGAX PE=1 SV=3
ANR24	17.91	1	1	124187	Ankyrin repeat domain-containing protein 24 OS=Homo sapiens GN=ANKRD24 PE=2 SV=2
ATX2L	17.83	1	1	113374	Ataxin-2-like protein OS=Homo sapiens GN=ATXN2L PE=1 SV=2
PKHG5	17.78	1	1	117451	Pleckstrin homology domain-containing family G member 5 OS=Homo sapiens GN=PLEKHG5 PE=1 SV=3
EDRF1	16.44	1	1	138528	Erythroid differentiation-related factor 1 OS=Homo sapiens GN=EDRF1 PE=1 SV=1

RGSL	16.44	1	1	125688	Regulator of G-protein signaling protein-like OS=Homo sapiens GN=RGSL1 PE=2 SV=1
E41L2	16.44	1	1	112588	Band 4.1-like protein 2 OS=Homo sapiens GN=EPB41L2 PE=1 SV=1
CSF1R	16.44	1	1	107984	Macrophage colony-stimulating factor 1 receptor OS=Homo sapiens GN=CSF1R PE=1 SV=2
PTN4	16.44	1	1	105911	Tyrosine-protein phosphatase non-receptor type 4 OS=Homo sapiens GN=PTPN4 PE=1 SV=1
CHSS3	16.44	1	1	100284	Chondroitin sulfate synthase 3 OS=Homo sapiens GN=CHSY3 PE=2 SV=3
PITM1	15.9	1	1	134847	Membrane-associated phosphatidylinositol transfer protein 1 OS=Homo sapiens GN=PITPNM1 PE=1 SV=4
NOD1	15.9	1	1	107691	Nucleotide-binding oligomerization domain-containing protein 1 OS=Homo sapiens GN=NOD1 PE=1 SV=1
XPF	15.9	1	1	104486	DNA repair endonuclease XPF OS=Homo sapiens GN=ERCC4 PE=1 SV=3
ZSWM6	15.69	1	1	133470	Zinc finger SWIM domain-containing protein 6 OS=Homo sapiens GN=ZSWIM6 PE=1 SV=2
ZSWM5	15.69	1	1	130633	Zinc finger SWIM domain-containing protein 5 OS=Homo sapiens GN=ZSWIM5 PE=2 SV=2
LCAP	15.39	1	1	117349	Leucyl-cystinyl aminopeptidase OS=Homo sapiens GN=LNPEP PE=1 SV=3
