# Supplementary material for: The snoRNA-like lncRNA LNC-SNO49AB drives leukemia by activating the RNA-editing enzyme ADAR1
Source: Cell Discov. 2022 Nov 1;8:117. doi: 10.1038/s41421-022-00460-9 (PMC9622897; doi:10.1038/s41421-022-00460-9)
Supplement: Supplementary file 10 — Supplemental Tab S3 [file 41421_2022_460_MOESM10_ESM.pdf]

Supplementary Table S3 list of dysregulated RNA editing level in LNC-SNO49AB knockdown.

Sites	NC_ Edlevel	Si-LNC-SNO49AB _Edlevel	Lev_ Change	P_value	Gene name	Annotation
chr17@54905381@-	0.7	0.143	-0.557	0.049774	C17orf67	Intron
chr21@47740295@-	0.667	0.111	-0.556	0.049774	C21orf58	Intron
chr3@182743969@-	0.889	0.333	-0.556	0.049774	MCCC1	Intron
chr3@37085996@+	0.111	0.625	0.514	0.049774	MLH1	Intron
chr6@150045919@-	0.28	0.043	-0.237	0.049707	NUP43	3UTR
chr1@109950438@-	0.174	0	-0.174	0.049645	PSMA5	Intron
chr1@85398059@-	0.25	0.45	0.2	0.049421	MCOLN2	Intron
chr16@29681565@+	0.016	0.002	-0.014	0.049322	SPN	3UTR
chr14@53243488@-	0.152	0.025	-0.127	0.0492	GNPNAT1	3UTR
chr9@4739355@-	0.5	0.176	-0.324	0.049005	AK3	Intron
chr1@109951973@-	0.278	0	-0.278	0.048754	PSMA5	Intron
chr6@42856964@+	0	0.018	0.018	0.04856	RPL7L1	3UTR
chr6@150045884@-	0.227	0	-0.227	0.048497	NUP43	3UTR
chr16@22522326@+	0.084	0	-0.084	0.048335	NPIPB5	Intron
chr16@28966426@+	0.104	0.024	-0.08	0.048279	NFATC2IP	Intron
chr17@26933400@-	0.2	0	-0.2	0.047842	RP11-192H23.4	Intron
chr10@135108204@-	0.8	0	-0.8	0.047619	TUBGCP2	Intron
chr10@51745662@+	1	0	-1	0.047619	Intergenic	Intergenic
chr10@70534194@+	0.8	0	-0.8	0.047619	CCAR1	Intron
chr10@99180817@-	0.833	0	-0.833	0.047619	AL355490.1	Intron
chr11@63524846@+	1	0	-1	0.047619	RTN3	Intron
chr11@64867189@+	1	0.167	-0.833	0.047619	VPS51	Intron
chr12@131288198@-	1	0.2	-0.8	0.047619	STX2	Intron
chr12@132330941@+	1	0.2	-0.8	0.047619	MMP17	Intron
chr12@57122797@-	1	0	-1	0.047619	NACA	Intron
chr14@93789269@-	1	0	-1	0.047619	BTBD7	Intron
chr15@28364697@-	0.8	0	-0.8	0.047619	HERC2	Intron
chr15@83698539@-	0.8	0	-0.8	0.047619	BTBD1	exon
chr16@69378556@+	1	0	-1	0.047619	Intergenic	Intergenic
chr16@87770976@-	1	0.2	-0.8	0.047619	KLHDC4	Intron
chr17@17737683@-	0.833	0	-0.833	0.047619	SREBF1	Intron
chr17@29103865@+	0.75	0	-0.75	0.047619	SUZ12P	Intron
chr17@60553407@-	0.75	0	-0.75	0.047619	Intergenic	Intergenic
chr17@7805003@+	0.8	0	-0.8	0.047619	CHD3	Intron
chr19@12009368@+	1	0	-1	0.047619	ZNF69	Intron
chr19@37306866@-	0.833	0	-0.833	0.047619	Intergenic	Intergenic
chr19@39895048@+	1	0	-1	0.047619	Intergenic	Intergenic

chr19@39895054@+	1	0	-1	0.047619	Intergenic	Intergenic
chr19@52796483@+	1	0	-1	0.047619	Intergenic	Intergenic
chr19@9924376@-	0.75	0	-0.75	0.047619	FBXL12	Intron
chr1@12593112@+	0	1	1	0.047619	Intergenic	Intergenic
chr1@12593115@+	0	1	1	0.047619	Intergenic	Intergenic
chr1@15818247@-	0.75	0	-0.75	0.047619	CASP9	3UTR
chr1@201487992@+	1	0	-1	0.047619	RP11-134G8.7	Intron
chr1@204521307@+	0.8	0	-0.8	0.047619	MDM4	3UTR
chr1@226050878@-	0.2	1	0.8	0.047619	TMEM63A	Intron
chr1@40269916@+	0.8	0	-0.8	0.047619	Intergenic	Intergenic
chr20@49502431@+	1	0	-1	0.047619	Intergenic	Intergenic
chr20@60760215@+	1	0	-1	0.047619	MTG2	Intron
chr2@114396289@+	0.833	0	-0.833	0.047619	RABL2A	Intron
chr2@170402592@-	1	0	-1	0.047619	FASTKD1	Intron
chr2@239003588@+	0.8	0	-0.8	0.047619	UBE2F-SCLY	Intron
chr2@242271026@+	0.75	0	-0.75	0.047619	2-Sep	Intron
chr2@242282150@+	1	0.2	-0.8	0.047619	2-Sep	Intron
chr3@121548994@-	0.833	0	-0.833	0.047619	IQCB1	Intron
chr3@183915199@+	0	1	1	0.047619	EIF2B5	Intron
chr3@194360426@-	0.167	1	0.833	0.047619	Intergenic	Intergenic
chr4@88949884@+	1	0	-1	0.047619	PKD2	Intron
chr5@145511278@-	0.8	0	-0.8	0.047619	LARS	exon
chr5@70859646@+	0	1	1	0.047619	BDP1	Intron
chr6@32132753@+	0.75	0	-0.75	0.047619	PPT2-EGFL8	Intron
chr6@34390431@-	0.75	0	-0.75	0.047619	RPS10-NUDT3	Intron
chr7@102042714@+	1	0	-1	0.047619	PRKRIP1	Intron
chr7@43661270@-	0.8	0	-0.8	0.047619	COA1	Intron
chr7@65853214@-	0.8	0	-0.8	0.047619	LINC00174	Intron
chr8@103255575@+	0.8	0	-0.8	0.047619	KB-431C1.4	Intron
chr8@12881603@+	0.833	0	-0.833	0.047619	KIAA1456	3UTR
chr9@139695975@-	0	1	1	0.047619	Intergenic	Intergenic
chrX@30584541@-	1	0	-1	0.047619	CXorf21	Intron
chr9@100775374@+	0.5	0.077	-0.423	0.047472	ANP32B	Intron
chr19@21475012@-	0	0.333	0.333	0.047431	ZNF708	3UTR
chr22@24079848@-	0.333	0	-0.333	0.047431	Intergenic	Intergenic
chr17@79529884@-	0.154	0	-0.154	0.047273	NPLOC4	3UTR
chr16@21886992@-	0.769	0.333	-0.436	0.04718	NPIPB4	Intron
chr13@28835819@+	0.211	0	-0.211	0.047124	PAN3	Intron

chr19@11041305@+	0.211	0	-0.211	0.047124	C19orf52	Intron
chr2@70445811@-	0.211	0	-0.211	0.047124	TIA1	Intron
chr19@17525558@+	0.429	0.205	-0.224	0.046939	CTD-2521M24.9	exon
chr8@129011982@+	0.5	0	-0.5	0.046801	PVT1	Intron
chr21@34637159@+	0.132	0.381	0.249	0.046782	AP000295.9	Intron
chr19@19901461@+	0.421	0.071	-0.35	0.04658	CTC-559E9.6	Intron
chr6@42854733@+	0	0.059	0.059	0.046528	RPL7L1	3UTR
chr16@89754697@+	0.8	0.44	-0.36	0.046363	CDK10	Intron
chr9@127711882@-	0.263	0	-0.263	0.046332	SCAI	3UTR
chr20@25426905@+	0.041	0	-0.041	0.046309	GINS1	3UTR
chr6@42175142@-	0.085	0	-0.085	0.046211	MRPS10	3UTR
chr19@1589704@-	0.17	0	-0.17	0.046142	UQCR11	Intron
chr8@94736368@-	0.812	0.4	-0.412	0.046141	Intergenic	Intergenic
chr22@36883967@-	0.462	0	-0.462	0.046078	FOXRED2	3UTR
chr1@41505334@-	0.143	0.571	0.428	0.046072	SCMH1	Intron
chr19@14681932@-	0.583	0.176	-0.407	0.045974	NDUFB7	Intron
chr5@64964627@-	0.5	0.062	-0.438	0.045796	SGTB	3UTR
chr14@53243412@-	0.171	0.042	-0.129	0.045558	GNPNAT1	3UTR
chr17@29225234@-	0.462	0	-0.462	0.045555	TEFM	Intron
chr19@35822631@+	0.174	0	-0.174	0.045508	U62631.5	exon
chr15@102285653@-	1	0.333	-0.667	0.045455	RP11-89K11.1	Intron
chr16@15794892@+	0.778	0	-0.778	0.045455	NDE1	Intron
chr22@18606435@+	0.6	0	-0.6	0.045455	PEX26	Intron
chr7@44053699@-	0.667	0	-0.667	0.045455	RP5-1165K10.2	Intron
chr8@56658410@-	0.667	0	-0.667	0.045455	TMEM68	exon
chr9@132674135@-	0.6	0	-0.6	0.045455	FNBP1	Intron
chr19@53150794@-	0.326	0.133	-0.193	0.045311	ZNF83	Intron
chr16@89834981@-	0.417	0	-0.417	0.045113	FANCA	Intron
chr16@89834982@-	0.417	0	-0.417	0.045113	FANCA	Intron
chr5@49709521@-	0.56	0.261	-0.299	0.045066	EMB	Intron
chr7@1545176@-	0.92	0.647	-0.273	0.044945	INTS1	Intron
chr9@115139586@-	0.833	0.286	-0.547	0.044911	Intergenic	Intergenic
chr6@36511530@-	0.3	0.048	-0.252	0.044825	STK38	Intron
chr12@133629013@+	1	0.417	-0.583	0.044118	ZNF84	Intron
chr1@231468076@-	0	0.5	0.5	0.044118	Intergenic	Intergenic
chr16@48388180@+	0.875	0.5	-0.375	0.044052	LONP2	3UTR
chr22@20848663@-	0.875	0.5	-0.375	0.044052	KLHL22	Intron
chr1@155285935@+	0.192	0.029	-0.163	0.04399	FDPS	Intron
chr12@133602346@+	0.556	0	-0.556	0.043956	Intergenic	Intergenic

chr13@21737087@-	0.556	0	-0.556	0.043956	SKA3	Intron
chr1@40269803@+	0.556	0	-0.556	0.043956	Intergenic	Intergenic
chr19@4653558@+	0.423	0.171	-0.252	0.04375	TNFAIP8L1	3UTR
chr19@17525783@+	0.14	0.019	-0.121	0.043712	CTD-2521M24.9	exon
chr16@1834178@+	0	0.231	0.231	0.043697	NUBP2	Intron
chr2@96495565@-	1	0.769	-0.231	0.043697	Intergenic	Intergenic
chr17@29121023@+	0.312	0	-0.312	0.043382	Intergenic	Intergenic
chr15@50657926@+	0.321	0.127	-0.194	0.04315	GABPB1-AS1	Intron
chr4@987999@+	0.548	0.308	-0.24	0.043128	IDUA	Intron
chr9@36216898@-	0.106	0	-0.106	0.043117	GNE	3UTR
chr3@10143169@+	0.055	0	-0.055	0.0431	FANCD2	3UTR
chr4@7028459@+	0.625	0.1	-0.525	0.042986	TBC1D14	Intron
chr19@39890840@+	0.067	0	-0.067	0.04293	MED29	3UTR
chr1@31990171@+	0.733	0	-0.733	0.042892	Intergenic	Intergenic
chr6@42856917@+	0.583	0.493	-0.09	0.042749	RPL7L1	3UTR
chr9@136033607@-	0.545	0	-0.545	0.042744	RALGDS	Intron
chrX@73228776@+	0.545	0	-0.545	0.042744	JPX	Intron
chr20@3851791@+	0.125	0.02	-0.105	0.042245	Intergenic	Intergenic
chr11@62385437@-	0.333	0	-0.333	0.042146	B3GAT3	Intron
chr19@58376936@+	0.286	0	-0.286	0.042146	Intergenic	Intergenic
chr5@69276760@+	0.229	0	-0.229	0.041997	Intergenic	Intergenic
chr1@89649557@-	1	0.429	-0.571	0.041899	GBP4	3UTR
chr12@27183192@+	0.108	0	-0.108	0.041759	MED21	3UTR
chr3@49361492@-	0.556	0.111	-0.445	0.041671	USP4	Intron
chr17@3578401@-	0	0.333	0.333	0.041502	P2RX5-TAX1BP3	Intron
chr1@145689763@+	0	0.107	0.107	0.041427	RNF115	3UTR
chr17@79960868@+	0.944	0.6	-0.344	0.041026	ASPSCR1	Intron
chr4@77867295@-	0.3	0	-0.3	0.041026	Intergenic	Intergenic
chr19@17525657@+	0.161	0.024	-0.137	0.040878	CTD-2521M24.9	exon
chr1@85397492@-	0.15	0.022	-0.128	0.040852	MCOLN2	Intron
chr12@54847032@-	0.625	0.091	-0.534	0.040764	Intergenic	Intergenic
chr12@108091613@+	1	0.692	-0.308	0.040741	PWP1	Intron
chr21@34965114@-	0.273	0	-0.273	0.04064	AP000304.12	Intron
chr1@9326997@+	0.145	0.021	-0.124	0.040628	H6PD	3UTR
chr16@57211379@-	0.75	0.125	-0.625	0.040559	FAM192A	Intron
chr19@58299292@-	0.714	0.125	-0.589	0.040559	Intergenic	Intergenic
chr2@219499832@-	0.167	0.778	0.611	0.040559	Intergenic	Intergenic
chr2@242191903@-	0.875	0.286	-0.589	0.040559	HDLBP	Intron

chr9@132676295@-	0.714	0.125	-0.589	0.040559	FNBP1	Intron
chr2@128602348@-	0	0.235	0.235	0.040404	RP5-935K16.1	exon
chr1@85397565@-	0.85	0.657	-0.193	0.040283	MCOLN2	Intron
chrX@47063399@+	0.5	0.059	-0.441	0.04009	UBA1	Intron
chr1@247262773@-	0.273	0.04	-0.233	0.040014	Intergenic	Intergenic
chr16@21440914@-	0.444	0.167	-0.277	0.03997	NPIPB3	Intron
chr22@20848503@-	0.417	0	-0.417	0.039645	KLHL22	Intron
chr1@1215445@+	0.346	0.083	-0.263	0.039514	RP5-902P8.10	Intron
chr16@22507786@+	0.534	0.367	-0.167	0.039489	NPIPB5	Intron
chr11@16762036@+	0.2	0.765	0.565	0.039341	C11orf58	Intron
chr7@74298166@-	0.182	0.458	0.276	0.039333	STAG3L2	Intron
chrX@100598421@-	0.458	0.182	-0.276	0.039333	Intergenic	Intergenic
chr11@20529204@+	0.25	0	-0.25	0.039244	PRMT3	Intron
chr3@160790871@+	0.175	0	-0.175	0.039235	PPM1L	3UTR
chr3@10195093@+	0.125	0	-0.125	0.039219	Intergenic	Intergenic
chr2@70449201@-	0	0.167	0.167	0.039184	TIA1	Intron
chrX@135300092@-	0.529	0.182	-0.347	0.039176	MAP7D3	3UTR
chr3@49357633@-	0.333	0	-0.333	0.03913	USP4	Intron
chrX@54589106@+	0.353	0.062	-0.291	0.039068	Intergenic	Intergenic
chr19@1590114@-	0.649	0.429	-0.22	0.0387	UQCR11	Intron
chr16@28842979@+	0.308	0	-0.308	0.038518	ATXN2L	Intron
chr16@69357374@-	0.146	0	-0.146	0.038499	COG8	Intron
chr2@96678384@+	1	0.333	-0.667	0.038462	FAHD2CP	Intron
chr19@11257704@-	0.578	0.354	-0.224	0.038435	SPC24	3UTR
chr1@145513112@+	0.211	0	-0.211	0.038274	RBM8A	3UTR
chr19@11257392@-	0.333	0.148	-0.185	0.038269	SPC24	3UTR
chr11@64506498@-	0.757	0.483	-0.274	0.038139	RASGRP2	Intron
chr11@118870551@+	0.8	0.214	-0.586	0.037926	CCDC84	Intron
chr4@40163922@+	0.455	0	-0.455	0.037926	Intergenic	Intergenic
chr6@76351462@+	0.6	0	-0.6	0.037926	SENP6	Intron
chr7@102742895@+	0.8	0.214	-0.586	0.037926	Intergenic	Intergenic
chrX@73417103@-	0.143	0.032	-0.111	0.037827	FTX	Intron
chr6@31720412@+	0.545	0	-0.545	0.037707	MSH5-SAPCD1	Intron
chr13@21738553@-	0.6	0.125	-0.475	0.037681	SKA3	Intron
chr4@2841553@+	0.722	0.312	-0.41	0.037403	SH3BP2	3UTR
chr16@22522358@+	0.511	0.327	-0.184	0.03734	NPIPB5	Intron
chr21@45644357@-	0.364	0	-0.364	0.037267	ICOSLG	3UTR
chrX@24095245@+	0.007	0	-0.007	0.037258	EIF2S3	3UTR
chr2@130915669@-	0.625	0.143	-0.482	0.037251	SMPD4	Intron
chr12@42825591@+	0.862	0.98	0.118	0.037034	PPHLN1	Intron
chr19@58372048@+	0.613	0.321	-0.292	0.037023	ZNF587	3UTR

chr3@156259314@-	0	0.075	0.075	0.036876	SSR3	3UTR
chr2@198356798@-	0.338	0.163	-0.175	0.036771	HSPD1	Intron
chr20@25669577@-	0.364	0.048	-0.316	0.036707	ZNF337	Intron
chr17@5365463@-	0	0.3	0.3	0.03663	DHX33	Intron
chr10@103570620@-	0.375	0	-0.375	0.036364	MGEA5	Intron
chr16@69338823@+	0.375	0	-0.375	0.036364	RP11-343C2.11	Intron
chr7@99093078@-	0.375	0	-0.375	0.036364	ZNF394	Intron
chr5@145525754@-	0.167	0	-0.167	0.036288	LARS	Intron
chr11@8708799@+	0.367	0.115	-0.252	0.036055	RPL27A	3UTR
chr10@74988494@-	1	0	-1	0.035714	DNAJC9	Intron
chr11@31453219@+	1	0	-1	0.035714	DNAJC24	3UTR
chr12@110837513@-	0.5	0	-0.5	0.035714	RP11-478C19.2	Intron
chr16@22412129@-	1	0	-1	0.035714	CDR2	Intron
chr16@24936653@-	1	0	-1	0.035714	ARHGAP17	Intron
chr17@61797720@-	1	0	-1	0.035714	STRADA	Intron
chr1@27204955@-	0.769	0	-0.769	0.035714	GPN2	3UTR
chr1@31738126@-	1	0	-1	0.035714	SNRNP40	Intron
chr3@16382770@+	1	0	-1	0.035714	OXNAD1	Intron
chr3@9806096@+	1	0	-1	0.035714	OGG1	Intron
chr4@25161272@-	1	0	-1	0.035714	SEPSECS	CDS
chr7@104589464@+	0.667	0.1	-0.567	0.035714	RP11-325F22.2	Intron
chr16@9204744@+	0.082	0	-0.082	0.035635	RP11-473I1.10	exon
chr3@52449313@+	0.308	0.08	-0.228	0.035629	PHF7	Intron
chr11@63589435@+	0.636	0.231	-0.405	0.035415	C11orf84	Intron
chr9@130874441@-	0.533	0.185	-0.348	0.035167	RP11-395P17.3	exon
chr2@3589521@-	0.113	0	-0.113	0.035154	RP13-512J5.1	Intron
chr11@118867599@-	0.455	0	-0.455	0.035088	RP11-110I1.12	exon
chr16@66858233@-	0.455	0	-0.455	0.035088	NAE1	Intron
chr22@23991065@-	0.455	0	-0.455	0.035088	GUSBP11	Intron
chr3@53898161@-	0.455	0	-0.455	0.035088	Intergenic	Intergenic
chr1@204524555@+	0.545	0.143	-0.402	0.034982	MDM4	3UTR
chr19@23533871@-	0.6	0	-0.6	0.034965	ZNF91	Intron
chr4@174249379@-	0.889	0.286	-0.603	0.034965	RP11-798M19.3	Intron
chrX@154353129@+	0.6	0	-0.6	0.034965	Intergenic	Intergenic
chr19@12045406@+	0.25	0	-0.25	0.034759	ZNF763	Intron

chr1@160966434@-	0.69	0.353	-0.337	0.034753	F11R	3UTR
chr3@139074076@+	0.188	0	-0.188	0.034536	MRPS22	Intron
chr12@121862453@+	0.714	0.1	-0.614	0.034502	RNF34	Intron
chr15@60714470@-	0	0.636	0.636	0.034502	NARG2	3UTR
chr17@5127110@+	0.818	0.167	-0.651	0.034502	RP11-333E1.1	Intron
chr6@6699593@-	0.636	0	-0.636	0.034502	RP1-80N2.2	Intron
chr10@96372497@+	0.097	0	-0.097	0.034431	HELLS	3UTR
chr16@21439963@-	0.393	0.125	-0.268	0.03436	NPIPB3	Intron
chr17@57281042@+	0.129	0	-0.129	0.034317	CTD-2510F5.6	Intron
chr2@179303649@+	0.129	0	-0.129	0.034317	AC009948.5	exon
chr2@98347263@+	0.304	0	-0.304	0.034302	ZAP70	Intron
chrX@73421646@-	0.167	0.035	-0.132	0.034215	RP3-368A4.5	exon
chr6@31717154@+	0.278	0.038	-0.24	0.034188	MSH5-SAPCD1	Intron
chr12@123112096@+	0.6	0	-0.6	0.033841	Intergenic	Intergenic
chr4@119623784@+	0.6	0	-0.6	0.033841	METTL14	Intron
chr7@99630537@+	0.6	0	-0.6	0.033841	ZKSCAN1	Intron
chr11@5719095@+	0.125	0.4	0.275	0.033774	TRIM22	exon
chr22@22084778@-	0.556	0	-0.556	0.033654	YPEL1	Intron
chr12@64801001@+	0.538	0.091	-0.447	0.033472	XPOT	Intron
chr19@28298888@+	0.348	0.08	-0.268	0.033471	LLNLF-65H9.1	Intron
chr11@17357097@+	1	0.25	-0.75	0.033333	NUCB2	Intron
chr11@73732212@-	0.75	0	-0.75	0.033333	C2CD3	Intron
chr16@1737188@+	0.857	0	-0.857	0.033333	HN1L	Intron
chr17@57772900@-	0.75	0	-0.75	0.033333	PTRH2	Intron
chr1@1340108@-	0.4	0	-0.4	0.033333	MRPL20	Intron
chr1@3778220@+	0.75	0	-0.75	0.033333	DFFB	Intron
chr1@86844801@-	0.75	0	-0.75	0.033333	ODF2L	Intron
chr3@15452538@-	0.857	0	-0.857	0.033333	METTL6	3UTR
chr5@126165189@+	0.4	0	-0.4	0.033237	LMNB1	Intron
chr16@22515193@+	0.254	0.097	-0.157	0.033016	NPIPB5	Intron
chr16@29524549@-	0.917	0	-0.917	0.032967	Intergenic	Intergenic
chr17@75088077@+	0	0.667	0.667	0.032967	LINC00338	Intron
chr13@28847461@+	0.133	0.018	-0.115	0.032907	PAN3	Intron
chr12@120899075@+	0.784	0.559	-0.225	0.032898	GATC	3UTR
chr20@43706098@+	0.141	0.053	-0.088	0.032887	STK4	3UTR
chr16@29680422@+	0.151	0.084	-0.067	0.032773	SPN	3UTR

chr1@1662764@-	0.606	0.304	-0.302	0.032763	RP1-283E3.8	Intron
chr15@50657927@+	0.786	0.537	-0.249	0.032614	GABPB1-AS1	Intron
chr3@178978451@+	0.898	0.956	0.058	0.032604	Intergenic	Intergenic
chr16@66923265@+	0.241	0	-0.241	0.032569	RP11-61A14.3	exon
chr17@43021910@-	0.5	0	-0.5	0.032508	KIF18B	Intron
chr14@102349629@-	0.004	0.011	0.007	0.032179	Intergenic	Intergenic
chr1@62915405@+	0.24	0	-0.24	0.032095	USP1	Intron
chr19@53065338@+	0.933	0.4	-0.533	0.031992	ZNF808	Intron
chr12@121772075@-	0.29	0.043	-0.247	0.031863	ANAPC5	exon
chr12@75890052@-	0.833	0.333	-0.5	0.031844	Intergenic	Intergenic
chr16@48386424@+	0.193	0.417	0.224	0.031664	LONP2	3UTR
chr12@56705447@-	0.375	0	-0.375	0.031621	CNPY2	exon
chr13@25009914@-	0.375	0	-0.375	0.031621	PARP4	Intron
chr6@42856983@+	0.474	0.577	0.103	0.031106	RPL7L1	3UTR
chr1@29498867@-	0.364	0	-0.364	0.031056	SRSF4	Intron
chr2@75890889@+	0.364	0	-0.364	0.031056	MRPL19	Intron
chr1@36065708@-	1	0.333	-0.667	0.030969	Intergenic	Intergenic
chr20@34141003@+	0.625	0	-0.625	0.030969	ERGIC3	Intron
chr20@48708807@-	0	0.667	0.667	0.030969	UBE2V1	Intron
chr22@23997826@-	0.667	0	-0.667	0.030969	KB-1572G7.2	Intron
chr13@25891636@+	0.643	0.125	-0.518	0.03096	NUPL1	Intron
chr2@62332882@-	0.9	0.417	-0.483	0.03096	AC018462.2	Intron
chr17@74949361@-	0.688	0.41	-0.278	0.030945	RP11-87G24.3	Intron
chrX@24096282@+	0.014	0	-0.014	0.03078	Intergenic	Intergenic
chr11@47191793@-	0.19	0	-0.19	0.030759	ARFGAP2	Intron
chr16@22296860@+	0.306	0.178	-0.128	0.030735	EEF2K	3UTR
chr7@128097770@+	0.02	0.163	0.143	0.030696	RP11-212P7.3	Intron
chr4@17803604@-	0.3	0.042	-0.258	0.030694	DCAF16	3UTR
chr10@70517289@+	0.522	0.19	-0.332	0.030692	CCAR1	Intron
chr6@42997464@+	0.607	0.25	-0.357	0.030692	RRP36	Intron
chr17@18209802@-	0.615	0.111	-0.504	0.030606	TOP3A	Intron
chr16@48387201@+	0.385	0.077	-0.308	0.030207	LONP2	3UTR
chr4@71682379@-	0.389	0	-0.389	0.030171	GRSF1	3UTR
chr2@37332016@-	0.065	0	-0.065	0.030092	EIF2AK2	3UTR
chr6@31721677@+	0.095	0	-0.095	0.029982	MSH5-SAPCD1	Intron
chr15@76228979@+	0.182	0.727	0.545	0.029973	Intergenic	Intergenic

chr3@176748737@-	0.8	0.273	-0.527	0.029973	TBL1XR1	Intron
chr16@89754742@+	0.273	0	-0.273	0.029928	CDK10	Intron
chr1@33148119@+	0.133	0	-0.133	0.0299	RBBP4	3UTR
chr2@130916022@-	0.806	0.538	-0.268	0.029671	SMPD4	Intron
chr11@65540403@-	1	0.5	-0.5	0.029412	Intergenic	Intergenic
chr12@46295587@+	0.556	0	-0.556	0.029412	ARID2	Intron
chr14@58760640@-	0.5	0	-0.5	0.029412	RP11-349A22.5	exon
chr1@27269758@+	0.5	0	-0.5	0.029412	NUDC	Intron
chr7@99748767@+	1	0.444	-0.556	0.029412	LAMTOR4	Intron
chr19@1587591@-	0.327	0.097	-0.23	0.029359	UQCR11	Intron
chr10@73994707@+	0.07	0.257	0.187	0.029301	ANAPC16	3UTR
chr16@75479470@-	0.387	0.1	-0.287	0.029266	RP11-77K12.1	Intron
chr1@1660958@-	0.825	0.633	-0.192	0.029264	RP1-283E3.8	Intron
chr17@62480024@-	0	0.368	0.368	0.029225	POLG2	Intron
chr19@48637852@-	0.368	0	-0.368	0.029225	LIG1	Intron
chr16@28966624@+	0.106	0.016	-0.09	0.029131	NFATC2IP	Intron
chr16@22507305@+	0.333	0.185	-0.148	0.029102	NPIPB5	Intron
chr1@155703676@+	0.188	0.6	0.412	0.029008	MSTO2P	Intron
chrX@73421795@-	0.347	0.16	-0.187	0.028811	RP3-368A4.5	exon
chr3@122333713@+	0.4	0	-0.4	0.028708	PARP15	Intron
chr10@112667097@-	1	0	-1	0.028571	BBIP1	exon
chr11@8676583@-	1	0	-1	0.028571	TRIM66	Intron
chr12@12903186@+	1	0	-1	0.028571	APOLD1	Intron
chr12@66515420@-	1	0	-1	0.028571	Intergenic	Intergenic
chr12@66515430@-	1	0	-1	0.028571	Intergenic	Intergenic
chr13@37410747@+	0	1	1	0.028571	Intergenic	Intergenic
chr16@68313644@+	1	0	-1	0.028571	SLC7A6	Intron
chr17@3579124@-	1	0	-1	0.028571	P2RX5-TAX1BP3	Intron
chr17@3808916@-	1	0	-1	0.028571	P2RX1	Intron
chr17@41299413@+	1	0	-1	0.028571	NBR2	Intron
chr17@74039245@-	1	0	-1	0.028571	SRP68	Intron
chr17@74773517@+	1	0	-1	0.028571	MFSD11	Intron
chr18@48470384@+	1	0	-1	0.028571	ME2	Intron
chr19@11935726@+	1	0	-1	0.028571	ZNF440	Intron
chr1@29501324@-	1	0	-1	0.028571	SRSF4	Intron
chr20@32437716@+	1	0	-1	0.028571	CHMP4B	Intron
chr2@114398052@+	1	0	-1	0.028571	RABL2A	exon
chr2@209172805@+	1	0	-1	0.028571	PIKFYVE	Intron
chr3@142209061@-	1	0	-1	0.028571	ATR	Intron

chr3@48479660@-	1	0	-1	0.028571	CCDC51	Intron
chr4@2655275@+	1	0	-1	0.028571	FAM193A	Intron
chr9@100814919@+	1	0	-1	0.028571	Intergenic	Intergenic
chr6@36511515@-	0.214	0	-0.214	0.028404	STK38	Intron
chr20@3848158@+	0.312	0.492	0.18	0.028324	MAVS	3UTR
chr1@10520076@-	0.545	0.111	-0.434	0.028086	DFFA	3UTR
chr11@62385900@-	0.286	0	-0.286	0.027836	B3GAT3	Intron
chr10@120932402@-	0.714	0	-0.714	0.027778	PRDX3	Intron
chr14@20919229@-	1	0	-1	0.027778	OSGEP	Intron
chr17@41370608@+	1	0	-1	0.027778	TMEM106 A	3UTR
chr20@50410259@-	1	0	-1	0.027778	SALL4	Intron
chr20@54970250@+	1	0	-1	0.027778	CSTF1	Intron
chr2@9561694@-	0.714	0	-0.714	0.027778	ITGB1BP1	Intron
chr9@131263809@+	1	0	-1	0.027778	Intergenic	Intergenic
chr11@43500312@+	0.667	0	-0.667	0.027772	TTC17	Intron
chr19@23400825@-	0.667	0	-0.667	0.027772	Intergenic	Intergenic
chr7@5517151@-	0.667	0	-0.667	0.027772	FBXL18	Intron
chr12@110289927@-	0.125	0	-0.125	0.027669	GLTP	3UTR
chr1@228365255@+	0.667	0.091	-0.576	0.027634	IBA57	3UTR
chr3@169521022@-	0.667	0.091	-0.576	0.027634	LRRC34	Intron
chr13@21949151@-	0.108	0.024	-0.084	0.02758	Intergenic	Intergenic
chr17@56418845@+	0.471	0.111	-0.36	0.027491	BZRAP1-AS1	Intron
chr15@60729967@-	0	0.6	0.6	0.027473	NARG2	Intron
chr16@2145303@-	0	0.6	0.6	0.027473	RP11-304L19.3	Intron
chr2@114355167@+	0.583	0.46	-0.123	0.027418	WASH2P	exon
chr7@128301415@+	0.144	0.044	-0.1	0.027388	Intergenic	Intergenic
chr12@53691057@+	0.4	0.05	-0.35	0.027371	PFDN5	exon
chr1@204526660@+	0.5	0.158	-0.342	0.027344	MDM4	3UTR
chr3@122333837@+	0.667	0.125	-0.542	0.027191	PARP15	Intron
chr3@179117033@-	0.148	0.043	-0.105	0.027155	GNB4	3UTR
chr7@65618545@+	0.041	0	-0.041	0.02709	CRCP	3UTR
chr16@23476506@-	0.108	0	-0.108	0.02705	GGA2	3UTR
chr2@220238219@-	0.128	0	-0.128	0.027038	Intergenic	Intergenic
chr17@49041732@-	0.154	0	-0.154	0.0268	SPAG9	3UTR
chr19@17525476@+	0.276	0.038	-0.238	0.026743	CTD-2521M24.9	exon
chr1@145369708@+	0.167	0.043	-0.124	0.026627	NBPF10	3UTR
chr20@5979776@+	0.615	0.2	-0.415	0.026538	Intergenic	Intergenic
chr17@37006249@-	0.25	0	-0.25	0.026528	RPL23	3UTR
chr7@156762772@+	0	0.105	0.105	0.026418	NOM1	3UTR
chr3@20211838@-	0.04	0.286	0.246	0.026387	SGOL1	3UTR

chr15@50658074@+	0.1	0	-0.1	0.026376	GABPB1-AS1	Intron
chr12@64802531@+	0.235	0.542	0.307	0.026223	XPOT	Intron
chr1@150593861@-	0	0.35	0.35	0.026212	ENSA	3UTR
chr6@56922828@+	0.667	0.167	-0.5	0.026209	Intergenic	Intergenic
chr7@100453006@+	0.368	0	-0.368	0.02614	SLC12A9	Intron
chrX@54590098@+	0.368	0	-0.368	0.02614	Intergenic	Intergenic
chr7@102185984@-	0.04	0.312	0.272	0.026067	RP11-514P8.7	Intron
chr19@58357264@+	0.364	0.042	-0.322	0.02582	ZNF587B	3UTR
chr9@139729282@+	0.444	0	-0.444	0.025795	RABL6	Intron
chr9@37778292@-	0.158	0	-0.158	0.025752	RP11-613M10.9	Intron
chr11@17356195@+	0.727	0	-0.727	0.025641	NUCB2	Intron
chr11@74677840@+	0.625	0	-0.625	0.025641	SPCS2	Intron
chr15@42833578@-	0.571	0	-0.571	0.025641	Intergenic	Intergenic
chr3@138335316@+	0.7	0	-0.7	0.025641	FAIM	Intron
chr7@44070881@-	0.625	0	-0.625	0.025641	RASA4CP	Intron
chr5@126164640@+	0.417	0.056	-0.361	0.025565	LMNB1	Intron
chr1@171561848@-	0	0.028	0.028	0.02508	Intergenic	Intergenic
chr7@100736991@+	0.548	0.317	-0.231	0.025076	Intergenic	Intergenic
chrX@2825119@-	0.92	0.783	-0.137	0.025066	ARSD	3UTR
chr4@185261977@-	0.32	0.215	-0.105	0.025061	RP11-290F5.2	exon
chr21@36170213@-	0.13	0.016	-0.114	0.025002	RUNX1	Intron
chr1@111679038@-	0.667	0	-0.667	0.025	DRAM2	Intron
chr19@55900533@+	0.873	0.722	-0.151	0.024832	RPL28	3UTR
chr12@124088715@+	0.385	0.05	-0.335	0.024789	DDX55	Intron
chr16@23478496@-	0.032	0	-0.032	0.024558	GGA2	3UTR
chr3@9860182@+	0.636	0.3	-0.336	0.024449	ARPC4-TTLL3	Intron
chr9@131049224@+	0.875	0	-0.875	0.024242	SWI5	Intron
chr9@132676381@-	0.75	0	-0.75	0.024242	FNBP1	Intron
chr19@10276161@-	0.389	0	-0.389	0.024191	DNMT1	Intron
chr3@121552737@-	0.444	0.209	-0.235	0.024006	IQCB1	Intron
chr2@202028832@+	0.316	0	-0.316	0.023895	CFLAR	3UTR
chr3@37113741@-	0.316	0	-0.316	0.023895	LRRFIP2	Intron
chr3@9855797@+	0.333	0	-0.333	0.0238	ARPC4-TTLL3	Intron
chr1@46165408@-	0	0.4	0.4	0.023746	IPP	3UTR
chr13@31023305@+	0.9	0.364	-0.536	0.023736	Intergenic	Intergenic
chr16@29920293@-	0.857	0.286	-0.571	0.023736	KCTD13	Intron
chr16@89754097@+	0.183	0.038	-0.145	0.023232	CDK10	3UTR
chr5@43574975@-	0.167	0	-0.167	0.023191	NNT-AS1	exon

chr19@58372752@+	0.303	0.065	-0.238	0.023064	ZNF587	3UTR
chr12@93898151@+	0.412	0	-0.412	0.023032	Intergenic	Intergenic
chr16@548356@+	0.429	0.062	-0.367	0.022986	RAB11FIP 3	Intron
chr19@20996737@+	0.8	0.111	-0.689	0.022977	Intergenic	Intergenic
chr19@19793781@+	0.5	0	-0.5	0.022876	ZNF101	3UTR
chr22@35798215@+	0.714	0	-0.714	0.022876	MCM5	Intron
chr3@15113893@-	0.5	0	-0.5	0.022876	ZFYVE20	3UTR
chr13@28846508@+	0.031	0.004	-0.027	0.022859	PAN3	Intron
chr2@152334893@+	0.737	0.273	-0.464	0.022746	RIF1	3UTR
chr1@109949640@-	0.429	0	-0.429	0.022727	PSMA5	Intron
chrX@118755490@-	0.273	0	-0.273	0.022628	6-Sep	Intron
chr16@3029224@-	0.189	0.024	-0.165	0.022579	PKMYT1	Intron
chr16@87777157@-	0.933	0.5	-0.433	0.02253	RP11-278A23.2	Intron
chr17@26932965@-	0.533	0	-0.533	0.022469	RP11-192H23.4	Intron
chr3@33587184@-	0.5	0	-0.5	0.022469	CLASP2	Intron
chr3@10194593@+	0.155	0.052	-0.103	0.022459	Intergenic	Intergenic
chr7@73642038@+	0.061	0	-0.061	0.022398	LAT2	Intron
chr22@36552344@-	0.625	0.15	-0.475	0.022303	APOL3	Intron
chr17@62779279@-	0.5	0	-0.5	0.022243	PLEKHM1 P	exon
chr10@75009663@-	0.103	0	-0.103	0.022225	MRPS16	3UTR
chr17@75086024@+	1	0	-1	0.022222	LINC0033 8	exon
chr19@55554662@+	1	0	-1	0.022222	CTC-550B14.7	Intron
chr2@109432649@+	1	0	-1	0.022222	CCDC138	Intron
chr3@169492776@+	1	0	-1	0.022222	MYNN	Intron
chr6@56942756@+	1	0	-1	0.022222	Intergenic	Intergenic
chr9@127966605@+	1	0	-1	0.022222	RABEPK	Intron
chr9@131263783@+	1	0	-1	0.022222	Intergenic	Intergenic
chr9@131263792@+	1	0	-1	0.022222	Intergenic	Intergenic
chr20@3851621@+	0.036	0.269	0.233	0.022191	Intergenic	Intergenic
chr6@150046511@-	0.55	0.25	-0.3	0.022188	NUP43	3UTR
chr6@42753077@+	0	0.364	0.364	0.022074	GLTSCR1 L	Intron
chr7@102204583@-	1	0.636	-0.364	0.022074	RP11-514P8.7	Intron
chr8@146102610@-	0.833	0	-0.833	0.021978	ZNF250	3UTR
chr7@73640568@+	0.545	0.143	-0.402	0.021965	LAT2	3UTR
chr3@58141791@+	0.094	0.031	-0.063	0.021667	FLNB	3UTR
chr2@37329217@-	0.182	0.039	-0.143	0.021637	EIF2AK2	3UTR

chr11@58914822@+	0.78	0.5	-0.28	0.021583	FAM111A	Intron
chr11@67773309@+	0.833	0.353	-0.48	0.021566	Intergenic	Intergenic
chr7@86791607@+	0	0.227	0.227	0.021554	DMTF1	Intron
chr16@87767788@-	0	0.4	0.4	0.021505	KLHDC4	Intron
chr12@125511958@+	0.567	0.333	-0.234	0.021443	BRI3BP	3UTR
chr1@204524546@+	0.7	0.238	-0.462	0.021391	MDM4	3UTR
chr19@12783257@-	0.632	0.278	-0.354	0.021386	Intergenic	Intergenic
chr5@175735195@+	0.8	0.952	0.152	0.021356	RP11-844P9.3	exon
chr15@40985001@-	0.714	0.214	-0.5	0.021301	RAD51-AS1	Intron
chr7@74296025@-	0.273	0	-0.273	0.02114	STAG3L2	Intron
chr1@85398168@-	0.095	0	-0.095	0.021098	MCOLN2	Intron
chr11@126078979@-	0.444	0	-0.444	0.021053	RPUSD4	exon
chr16@89803872@-	0.444	0	-0.444	0.021053	Intergenic	Intergenic
chr1@159021039@+	0.444	0	-0.444	0.021053	IFI16	Intron
chr17@29107016@+	1	0.25	-0.75	0.020979	SUZ12P	Intron
chr1@201484762@+	0.667	0	-0.667	0.020979	RP11-134G8.7	Intron
chr4@57213865@-	0.714	0	-0.714	0.020979	AASDH	Intron
chr4@57901738@-	1	0.286	-0.714	0.020979	IGFBP7	Intron
chr5@74673831@-	0.778	0	-0.778	0.020979	COL4A3BP	Intron
chr8@52768501@-	1	0.25	-0.75	0.020979	PCMTD1	Intron
chr16@69356839@-	0.129	0	-0.129	0.020942	COG8	Intron
chr19@1459301@-	0.565	0.1	-0.465	0.02094	CTB-25B13.12	Intron
chr16@29679080@+	0.027	0	-0.027	0.02077	SPN	3UTR
chr7@97598597@-	0.267	0	-0.267	0.020668	AC004967.7	Intron
chr11@77634281@-	0.5	0	-0.5	0.02064	INTS4	Intron
chr20@61528074@-	0.602	0.426	-0.176	0.020595	DIDO1	CDS
chr12@125512360@+	0.375	0	-0.375	0.020471	BRI3BP	3UTR
chr7@64464547@-	0.375	0	-0.375	0.020471	ERV3-1	exon
chr9@37164170@+	0.763	0.927	0.164	0.020429	ZCCHC7	Intron
chr12@9844945@+	0.765	0.35	-0.415	0.020202	CLEC2D	Intron
chr16@21886983@-	0.263	0	-0.263	0.020196	NPIPB4	Intron
chr1@33147928@+	0.113	0.029	-0.084	0.020169	RBBP4	3UTR
chr1@33147915@+	0.073	0.009	-0.064	0.01999	RBBP4	3UTR
chr16@29470210@+	0.318	0.074	-0.244	0.019921	SLX1B-SULT1A4	Intron
chr9@130582497@+	0.28	0.036	-0.244	0.019913	RP11-228B15.4	Intron
chr2@86252043@-	0.147	0	-0.147	0.019887	POLR1A	3UTR

chr3@196079697@-	0.429	0	-0.429	0.019763	UBXN7	3UTR
chr16@548440@+	0.316	0	-0.316	0.019656	RAB11FIP3	Intron
chr17@47789386@-	0.667	0	-0.667	0.019608	FAM117A	Intron
chr16@29679831@+	0.478	0.377	-0.101	0.019566	SPN	3UTR
chr1@18368@-	0.034	0.208	0.174	0.019512	WASH7P	exon
chrX@119571849@-	0.134	0.039	-0.095	0.019459	LAMP2	3UTR
chr5@150639439@+	0.472	0.617	0.145	0.019396	GM2A	CDS
chr19@17528806@+	0.571	0	-0.571	0.019231	CTD-2521M24.9	Intron
chr15@58745899@+	0.286	0	-0.286	0.019118	LIPC	Intron
chr2@37331555@-	0.579	0.1	-0.479	0.019086	EIF2AK2	3UTR
chr1@33148282@+	0.367	0.171	-0.196	0.019075	RBBP4	3UTR
chr1@28145573@+	0.562	0	-0.562	0.018943	STX12	Intron
chr14@50793612@+	0.192	0	-0.192	0.018909	ATP5S	exon
chr20@3851657@+	0.069	0.333	0.264	0.018374	Intergenic	Intergenic
chrX@73219017@+	0.235	0	-0.235	0.018326	JPX	exon
chr11@9312603@-	0.688	0.182	-0.506	0.018307	TMEM41B	Intron
chr14@20738672@-	1	0.111	-0.889	0.018182	TTC5	Intron
chr15@45025808@+	1	0	-1	0.018182	TRIM69	exon
chr15@58720001@+	0.75	0	-0.75	0.018182	LIPC	Intron
chr19@48689058@+	0	0.75	0.75	0.018182	C19orf68	Intron
chr7@114655313@+	1	0	-1	0.018182	MDFIC	Intron
chr8@126045876@-	0	0.889	0.889	0.018182	KIAA0196	Intron
chrX@73208536@+	0.889	0	-0.889	0.018182	JPX	Intron
chr16@22515194@+	0.5	0.281	-0.219	0.018099	NPIPB5	Intron
chr11@118870652@+	0.273	0	-0.273	0.018054	CCDC84	Intron
chr13@25010598@-	1	0.154	-0.846	0.017857	PARP4	Intron
chr17@17737679@-	1	0	-1	0.017857	SREBF1	Intron
chr17@47789506@-	0.6	0	-0.6	0.017857	FAM117A	Intron
chr19@1602079@-	1	0	-1	0.017857	UQCR11	Intron
chr20@62508694@+	1	0	-1	0.017857	TPD52L2	Intron
chr2@53928239@-	0	0.6	0.6	0.017857	ASB3	Intron
chr8@145705999@+	1	0	-1	0.017857	PPP1R16A	Intron
chrX@15810387@+	1	0	-1	0.017857	ZRSR2	Intron
chr18@23749070@-	0.1	0.012	-0.088	0.017809	Intergenic	Intergenic
chr11@66397431@+	0.5	0	-0.5	0.017544	RBM14-RBM4	Intron
chr6@111588945@+	0	0.5	0.5	0.017544	KIAA1919	3UTR
chr19@1590509@-	0.099	0.259	0.16	0.017502	UQCR11	Intron
chr6@42047354@+	0.216	0.08	-0.136	0.017496	TAF8	3UTR
chr7@100426891@-	0.583	0	-0.583	0.017405	Intergenic	Intergenic
chr1@33148196@+	0.303	0.132	-0.171	0.017378	RBBP4	3UTR
chr7@64464473@-	0.429	0	-0.429	0.017292	ERV3-1	exon

chr16@11928846@-	0.391	0	-0.391	0.017245	Intergenic	Intergenic
chr12@54846969@-	0.444	0	-0.444	0.017225	Intergenic	Intergenic
chrX@130875372@-	0.9	0.2	-0.7	0.016983	RP11-453F18__B.1	Intron
chrX@73428239@-	0.8	0.1	-0.7	0.016983	RP3-368A4.5	Intron
chr16@28839860@+	0.636	0.143	-0.493	0.016837	ATXN2L	Intron
chr11@13725471@+	0.739	0.308	-0.431	0.016816	FAR1	Intron
chr12@125512073@+	0.5	0.214	-0.286	0.01681	BRI3BP	3UTR
chr18@32828603@+	0.181	0.046	-0.135	0.016719	ZNF397	Intron
chr17@5083472@-	0.267	0	-0.267	0.016596	ZNF594	3UTR
chr6@42047492@+	0.413	0.162	-0.251	0.016554	TAF8	3UTR
chr7@102090258@+	0.075	0	-0.075	0.016535	ORAI2	3UTR
chr19@58268274@+	0.143	0	-0.143	0.016431	ZNF776	3UTR
chr1@41505342@-	0.133	0.615	0.482	0.016323	SCMH1	Intron
chr7@74296346@-	0.506	0.314	-0.192	0.016255	STAG3L2	Intron
chr3@10192720@+	0.015	0.167	0.152	0.016223	VHL	3UTR
chr4@185260430@-	0.013	0.12	0.107	0.016199	Intergenic	Intergenic
chr7@44620446@-	0.429	0	-0.429	0.015942	TMED4	3UTR
chrX@77083118@-	0.13	0	-0.13	0.015863	MAGT1	3UTR
chr11@9771767@+	0.12	0.024	-0.096	0.015846	SWAP70	3UTR
chr19@4653139@+	0.242	0	-0.242	0.015815	TNFAIP8L1	3UTR
chr19@53101253@+	0.368	0.045	-0.323	0.01574	Intergenic	Intergenic
chr21@45643965@-	0.273	0.036	-0.237	0.015675	ICOSLG	3UTR
chr4@83817800@-	0.132	0	-0.132	0.015636	THAP9-AS1	Intron
chr14@100762714@-	0.843	0.6	-0.243	0.015551	SLC25A29	5UTR
chr12@98990652@+	0.667	0.263	-0.404	0.01555	SLC25A3	Intron
chr19@49992562@+	0.5	0.933	0.433	0.015499	RPL13A	Intron
chr21@34923319@+	0.029	0.003	-0.026	0.015336	SON	CDS
chr1@167609900@+	0	0.119	0.119	0.015316	RCSD1	exon
chr17@26932641@-	0.48	0.077	-0.403	0.015241	RP11-192H23.4	Intron
chr7@128301263@+	0.124	0.016	-0.108	0.015225	Intergenic	Intergenic
chr11@9315225@-	0.778	0.125	-0.653	0.01522	TMEM41B	Intron
chr4@119622531@+	0.8	0.143	-0.657	0.01522	METTL14	Intron
chr12@132330946@+	1	0.2	-0.8	0.015152	MMP17	Intron
chr15@67504505@-	0.8	0	-0.8	0.015152	AAGAB	Intron
chr16@24935629@-	1	0.143	-0.857	0.015152	ARHGAP17	Intron
chr16@69415528@-	1	0	-1	0.015152	TERF2	Intron

chr16@87777103@-	0.857	0	-0.857	0.015152	RP11-278A23.2	Intron
chr17@27885601@-	0.833	0	-0.833	0.015152	Intergenic	Intergenic
chr17@27885610@-	0.833	0	-0.833	0.015152	Intergenic	Intergenic
chr17@73792463@+	0.857	0	-0.857	0.015152	UNK	Intron
chr18@56420526@+	0.857	0	-0.857	0.015152	RP11-108P20.1	exon
chr19@45453817@-	0	0.857	0.857	0.015152	CTB-129P6.11	Intron
chr5@64826637@-	0.857	0	-0.857	0.015152	CENPK	Intron
chr6@137108055@+	0.833	0	-0.833	0.015152	Intergenic	Intergenic
chr6@76409730@+	0.857	0	-0.857	0.015152	SENP6	Intron
chr3@156259340@-	0.683	0.429	-0.254	0.01502	SSR3	3UTR
chr6@150045998@-	0.333	0.043	-0.29	0.014914	NUP43	3UTR
chr19@20010683@+	0.6	0	-0.6	0.014706	Intergenic	Intergenic
chr19@37736441@+	0.6	0	-0.6	0.014706	Intergenic	Intergenic
chr2@62330050@-	0.333	0.036	-0.297	0.014563	AC018462.2	Intron
chr20@43256321@-	0.219	0	-0.219	0.014549	ADA	Intron
chr3@10143253@+	0.085	0.01	-0.075	0.014341	FANCD2	3UTR
chr2@37329214@-	0.318	0.115	-0.203	0.014245	EIF2AK2	3UTR
chr3@122333847@+	0	0.444	0.444	0.014229	PARP15	Intron
chrX@2824433@-	0.754	0.5	-0.254	0.014145	ARSD	3UTR
chrX@118755701@-	0.184	0	-0.184	0.014135	6-Sep	Intron
chr17@37336458@-	0.545	0	-0.545	0.014087	CACNB1	Intron
chr7@66312186@+	0.545	0	-0.545	0.014087	Intergenic	Intergenic
chr19@12782285@-	0.1	1	0.9	0.013986	Intergenic	Intergenic
chr6@13651759@-	0.75	0	-0.75	0.013986	RANBP9	Intron
chr19@1587698@-	0.092	0	-0.092	0.013961	UQCR11	Intron
chr2@160093888@-	0.667	0.071	-0.596	0.013932	WDSUB1	Intron
chr7@44620424@-	0.625	0.077	-0.548	0.013932	TMED4	3UTR
chr14@23418549@-	0.571	0.083	-0.488	0.013814	RP11-298I3.5	Intron
chr5@37290566@-	0.892	0.615	-0.277	0.013812	NUP155	3UTR
chr5@130537181@+	0.186	0	-0.186	0.013785	LYRM7	3UTR
chr12@121769691@-	0.327	0.074	-0.253	0.013616	ANAPC5	exon
chr17@57281058@+	0.438	0.143	-0.295	0.01351	CTD-2510F5.6	Intron
chr1@144829720@+	0.381	0.074	-0.307	0.013477	NBPF9	3UTR
chr19@4654537@+	0.175	0	-0.175	0.013349	TNFAIP8L1	3UTR
chr10@73994767@+	0.184	0	-0.184	0.013335	ANAPC16	3UTR
chr1@16948999@-	0.205	0.024	-0.181	0.013302	CROCCP2	Intron
chr19@12783178@-	0.574	0.31	-0.264	0.013255	Intergenic	Intergenic

chr17@2320651@-	0.6	0.303	-0.297	0.013074	METTL16	3UTR
chr17@74947271@-	0	0.278	0.278	0.013021	RP11-87G24.3	Intron
chr9@140103239@+	0.643	0.1	-0.543	0.012878	NDOR1	Intron
chr19@53383696@-	0.161	0	-0.161	0.012876	ZNF320	3UTR
chr19@5709756@-	0.048	0.5	0.452	0.01285	LONP1	Intron
chrX@73421663@-	0.3	0.088	-0.212	0.012849	RP3-368A4.5	exon
chr7@104612707@+	0.44	0.115	-0.325	0.01283	Intergenic	Intergenic
chr11@5689818@-	0.636	0	-0.636	0.012821	TRIM5	Intron
chr20@3088980@-	1	0.364	-0.636	0.012821	UBOX5	3UTR
chr9@99664630@-	1	0.4	-0.6	0.012821	HIATL2	Intron
chrX@153643677@+	0.636	0	-0.636	0.012821	TAZ	Intron
chr12@120898729@+	0.346	0.833	0.487	0.012767	GATC	3UTR
chr19@53121887@-	0	0.127	0.127	0.012754	ZNF83	3UTR
chr11@67854935@-	0.684	0.125	-0.559	0.012754	CHKA	Intron
chr17@19349456@-	0.995	1	0.005	0.012714	AC004448.5	Intron
chr5@121362875@+	0.3	0.102	-0.198	0.012661	SRFBP1	3UTR
chr4@69242133@+	0.053	0.019	-0.034	0.012638	MT2P1	exon
chr5@71516156@-	0.043	0.008	-0.035	0.012581	MRPS27	3UTR
chr17@27886620@-	0.3	0.033	-0.267	0.012418	Intergenic	Intergenic
chr16@28969406@+	0.5	0	-0.5	0.012384	NFATC2IP	Intron
chr16@543736@+	0.545	0	-0.545	0.012384	RAB11FIP3	Intron
chr19@58357271@+	0.75	0.28	-0.47	0.012377	ZNF587B	3UTR
chr12@120606978@-	0.793	0.412	-0.381	0.012355	GCN1L1	Intron
chr22@24505439@+	0.6	0	-0.6	0.012255	CABIN1	Intron
chr16@89648315@+	0.308	0	-0.308	0.012138	CPNE7	Intron
chr16@11018191@+	0.067	0.007	-0.06	0.012001	CIITA	3UTR
chr16@85734115@-	1	0	-1	0.011905	C16orf74	Intron
chr2@32539578@+	1	0	-1	0.011905	YIPF4	3UTR
chr4@773297@-	0.526	0	-0.526	0.011889	RP11-440L14.1	Intron
chr12@69158117@+	0.833	0.211	-0.622	0.011858	SLC35E3	Intron
chr16@21441018@-	0.05	0.429	0.379	0.011802	NPIPB3	Intron
chr22@45573271@+	0.226	0	-0.226	0.011699	NUP50	Intron
chr9@100775399@+	0.5	0	-0.5	0.011696	ANP32B	Intron
chr15@66628805@-	0.185	0	-0.185	0.011486	TIPIN	3UTR
chr20@48560699@+	0.571	0	-0.571	0.011438	RNF114	Intron
chr7@74296420@-	0.286	0.071	-0.215	0.011412	STAG3L2	Intron
chr4@87844347@-	0.429	0.067	-0.362	0.011406	C4orf36	Intron
chr16@11018312@+	0.074	0.013	-0.061	0.011366	CIITA	3UTR
chr21@36226806@-	1	0.333	-0.667	0.011364	RUNX1	Intron

chr17@2321606@-	0.233	0	-0.233	0.011358	METTL16	3UTR
chr22@26872510@-	0.345	0.686	0.341	0.011356	HPS4	exon
chr16@30264379@-	0.672	0.417	-0.255	0.01128	RP11-347C12.1	Intron
chr15@100328941@+	0.583	0.077	-0.506	0.011167	Intergenic	Intergenic
chr17@7805724@+	0.583	0.077	-0.506	0.011167	CHD3	Intron
chr12@98942643@+	0.065	0.007	-0.058	0.011092	TMPO	3UTR
chr10@12194219@+	0.214	0.8	0.586	0.011088	SEC61A2	Intron
chr11@62506299@+	0.19	0.021	-0.169	0.011028	TTC9C	Intron
chr19@37736454@+	0.667	0	-0.667	0.010989	Intergenic	Intergenic
chr16@22504924@+	0.321	0.113	-0.208	0.010888	NPIPB5	Intron
chr12@93898249@+	0	0.6	0.6	0.010836	Intergenic	Intergenic
chr15@83668161@-	0.692	0	-0.692	0.010836	C15orf40	Intron
chr9@131089937@+	1	0.4	-0.6	0.010836	COQ4	Intron
chr22@45573201@+	0.282	0	-0.282	0.010712	NUP50	Intron
chr19@13871486@+	0.378	0.097	-0.281	0.010704	CCDC130	Intron
chr19@17655814@+	0.224	0.027	-0.197	0.010619	FAM129C	Intron
chr1@33417060@+	0.776	0.973	0.197	0.010619	Intergenic	Intergenic
chr15@50652848@+	0.278	0.051	-0.227	0.010614	GABPB1-AS1	Intron
chr19@53998144@+	0.045	0.25	0.205	0.010357	ZNF813	3UTR
chr4@57326126@+	0.203	0.109	-0.094	0.01034	PAICS	3UTR
chr22@23919936@-	0	0.235	0.235	0.010334	IGLL1	Intron
chr3@9860263@+	0.297	0	-0.297	0.010272	ARPC4-TTLL3	Intron
chr16@21872269@-	0.217	0.593	0.376	0.010235	NPIPB4	Intron
chr1@145369498@+	0.271	0.054	-0.217	0.010219	NBPF10	3UTR
chr13@50122343@-	1	0.125	-0.875	0.010101	RCBTB1	Intron
chr17@1656461@+	0.8	0	-0.8	0.010101	SERPINF2	Intron
chr20@3088923@-	0.8	0	-0.8	0.010101	UBOX5	3UTR
chrX@129342755@-	0.8	0	-0.8	0.010101	ZNF280C	Intron
chr10@96372507@+	0	0.115	0.115	0.010053	HELLS	3UTR
chr22@45573203@+	0.306	0	-0.306	0.010046	NUP50	Intron
chr19@17663490@+	0.452	0.17	-0.282	0.010039	FAM129C	3UTR
chr9@115447479@-	0.259	0	-0.259	0.010029	INIP	3UTR
chr11@118870269@+	0.444	0	-0.444	0.009997	CCDC84	Intron
chr19@21303034@+	0	0.444	0.444	0.00996	ZNF714	3UTR
chr1@92744902@-	0.545	0.077	-0.468	0.009884	GLMN	Intron
chr19@39982329@+	0.35	0.074	-0.276	0.009875	TIMM50	exon
chr6@42905085@+	0.269	0	-0.269	0.009834	CNPY3	Intron
chr19@58378350@+	0.318	0	-0.318	0.009827	Intergenic	Intergenic
chr15@50653040@+	0.162	0	-0.162	0.009805	GABPB1-AS1	Intron
chr20@61563506@-	0.75	0	-0.75	0.009657	DIDO1	Intron

chr19@48637832@-	0.444	0	-0.444	0.009626	LIG1	Intron
chr1@144829583@+	0.27	0	-0.27	0.009368	NBPF9	3UTR
chr16@3710896@+	0.8	0.118	-0.682	0.009342	DNASE1	exon
chr20@17943121@-	0.571	0.19	-0.381	0.009305	OVOL2	Intron
chr19@13870963@+	0.184	0	-0.184	0.009117	CCDC130	Intron
chr19@14681778@-	0.667	0	-0.667	0.00905	NDUFB7	Intron
chr2@209173606@+	1	0.375	-0.625	0.00905	PIKFYVE	Intron
chr6@31716275@+	0.625	0	-0.625	0.00905	MSH5-SAPCD1	Intron
chr19@17660891@+	0.385	0	-0.385	0.009031	FAM129C	Intron
chr11@126164873@+	0.533	0.12	-0.413	0.009013	TIRAP	Intron
chr5@130538308@+	0	0.182	0.182	0.008982	LYRM7	3UTR
chr13@28854116@+	0.6	0.071	-0.529	0.008841	PAN3	Intron
chr3@25775662@-	0.6	0.071	-0.529	0.008841	NGLY1	exon
chr1@201929994@+	0.75	0	-0.75	0.008791	TIMM17A	Intron
chr15@50661858@+	0.2	0	-0.2	0.008739	Intergenic	Intergenic
chr17@62466487@+	0.312	0	-0.312	0.008702	Intergenic	Intergenic
chr4@185262085@-	0.153	0.254	0.101	0.008648	RP11-290F5.2	exon
chr12@123243123@+	0.323	0	-0.323	0.008627	DENR	Intron
chr19@21302864@+	0.387	0	-0.387	0.008522	ZNF714	3UTR
chr11@67773314@+	0.357	0	-0.357	0.008435	Intergenic	Intergenic
chr1@19655675@-	0.444	0	-0.444	0.008428	Intergenic	Intergenic
chr4@71682381@-	0.632	0.1	-0.532	0.00841	GRSF1	3UTR
chr9@131263736@+	1	0	-1	0.008333	Intergenic	Intergenic
chrX@73210784@+	1	0	-1	0.008333	JPX	Intron
chr13@32973189@+	0.203	0.037	-0.166	0.008318	BRCA2	3UTR
chr3@15452554@-	1	0.333	-0.667	0.008242	METTL6	3UTR
chr16@22515225@+	0.765	0.5	-0.265	0.008233	NPIPB5	Intron
chr8@48890109@+	0.167	0.112	-0.055	0.008212	MCM4	3UTR
chr16@22515116@+	0.672	0.5	-0.172	0.0082	NPIPB5	Intron
chr3@50373260@-	0.162	0	-0.162	0.008182	RASSF1	Intron
chr7@128142904@+	0.174	0	-0.174	0.00817	METTL2B	3UTR
chr8@124360308@-	0.179	0	-0.179	0.00812	ATAD2	Intron
chr1@53291425@+	0.412	0.087	-0.325	0.008067	ZYG11B	3UTR
chr12@133272845@+	1	0	-1	0.007937	RP13-672B3.2	Intron
chr12@42753700@+	1	0	-1	0.007937	PPHLN1	Intron
chr16@29921779@-	1	0	-1	0.007937	KCTD13	Intron
chr19@38224615@-	1	0	-1	0.007937	Intergenic	Intergenic
chr1@109946169@-	1	0	-1	0.007937	PSMA5	Intron
chr21@15432725@+	1	0	-1	0.007937	AP001347.6	Intron
chr5@64826654@-	1	0	-1	0.007937	CENPK	Intron

chr8@124122255@+	1	0	-1	0.007937	TBC1D31	Intron
chrX@73177282@+	1	0	-1	0.007937	JPX	Intron
chr4@185262071@-	0.28	0.17	-0.11	0.007811	RP11-290F5.2	exon
chr3@52723782@+	0.706	0.167	-0.539	0.007775	GNL3	Intron
chr10@51733352@+	0.167	0	-0.167	0.007714	LINC00843	Intron
chr18@33745924@+	0.048	0.571	0.523	0.007692	ELP2	Intron
chr1@145513094@+	0.389	0	-0.389	0.007625	RBM8A	3UTR
chr4@119221381@-	0.098	0.333	0.235	0.007611	PRSS12	Intron
chr4@774116@-	0.227	0	-0.227	0.00757	RP11-440L14.1	exon
chr1@33149256@+	0.306	0.073	-0.233	0.007542	RBBP4	3UTR
chr5@32382341@-	0.231	0.889	0.658	0.007519	ZFR	Intron
chr12@57121335@-	0.385	0	-0.385	0.00748	NACA	Intron
chr19@5909783@+	0.633	0.259	-0.374	0.007462	AC104532.2	Intron
chr20@48735434@-	0.778	0.154	-0.624	0.00732	TMEM189	Intron
chr12@98943033@+	0.849	0.6	-0.249	0.007269	TMPO	3UTR
chr15@50653027@+	0.176	0	-0.176	0.00726	GABPB1-AS1	Intron
chr15@91507234@+	0.571	0	-0.571	0.007224	Intergenic	Intergenic
chr4@39835147@-	0.714	0.077	-0.637	0.007224	PDS5A	Intron
chr6@31721683@+	0.127	0	-0.127	0.007205	MSH5-SAPCD1	Intron
chr11@88055700@-	0.206	0.023	-0.183	0.007079	CTSC	3UTR
chr12@9844915@+	0.4	0.04	-0.36	0.007057	CLEC2D	Intron
chr14@102513236@+	0.75	0	-0.75	0.006993	DYNC1H1	Intron
chr1@201485050@+	0	0.75	0.75	0.006993	RP11-134G8.7	Intron
chrX@130875381@-	0.8	0	-0.8	0.006993	RP11-453F18__B.1	Intron
chr1@36068275@-	0.273	0	-0.273	0.006949	PSMB2	3UTR
chr9@99654044@-	0.273	0	-0.273	0.006949	Intergenic	Intergenic
chr19@58417912@-	0.714	0.111	-0.603	0.006948	CTD-2583A14.9	Intron
chr14@100763003@-	0.857	0.381	-0.476	0.006928	SLC25A29	Intron
chr7@100452736@+	0.917	0.5	-0.417	0.006838	SLC12A9	Intron
chr11@62506385@+	0.133	0	-0.133	0.006833	TTC9C	Intron
chr1@24299071@-	0.6	0	-0.6	0.006823	SRSF10	Intron
chr11@88055688@-	0.654	0.357	-0.297	0.006674	CTSC	3UTR
chr6@43583172@+	0.365	0.094	-0.271	0.006632	POLH	3UTR
chr19@53579185@-	0.625	0	-0.625	0.006536	ZNF160	5UTR

chr2@96496491@-	0.941	0.542	-0.399	0.006433	Intergenic	Intergenic
chr16@28926520@-	1	0.375	-0.625	0.006429	RABEP2	Intron
chr19@13870306@+	0.353	0	-0.353	0.006354	CCDC130	3UTR
chr3@197339878@-	0.231	0	-0.231	0.006344	AC024560.3	Intron
chr3@50133653@+	0.467	0	-0.467	0.006322	RBM6	Intron
chr19@16754467@-	0.846	0	-0.846	0.006303	CTC-429P9.4	Intron
chr4@185260491@-	0.586	0.409	-0.177	0.006241	Intergenic	Intergenic
chr8@48890084@+	0.014	0	-0.014	0.0062	MCM4	3UTR
chr19@6584626@-	0.556	0	-0.556	0.006192	CD70	Intron
chr13@111552387@-	0.625	0.383	-0.242	0.006164	ANKRD10	Intron
chr20@43706947@+	0.821	0.587	-0.234	0.006141	STK4	3UTR
chr1@220231484@-	0.22	0.047	-0.173	0.006125	BPNT1	3UTR
chr17@5083520@-	0.273	0	-0.273	0.00608	ZNF594	3UTR
chr2@97936714@+	0.577	0.158	-0.419	0.006076	Intergenic	Intergenic
chr1@20978523@-	0.046	0	-0.046	0.006063	DDOST	3UTR
chr10@18941769@-	1	0	-1	0.006061	RP11-139J15.7	Intron
chr22@42964815@-	1	0	-1	0.006061	RRP7B	Intron
chr16@592643@+	0.549	0.31	-0.239	0.00601	CAPN15	Intron
chr11@93468592@-	0.214	0.079	-0.135	0.005983	TAF1D	exon
chr13@28863612@+	0.108	0.011	-0.097	0.005937	PAN3	Intron
chr1@11089825@+	0.062	0.533	0.471	0.005923	RP4-635E18.8	exon
chr19@51872982@-	0.538	0	-0.538	0.005912	Intergenic	Intergenic
chr11@5689464@-	1	0.538	-0.462	0.005797	TRIM5	Intron
chr4@185261203@-	0.478	0.652	0.174	0.005782	Intergenic	Intergenic
chr6@42856110@+	0.949	0.835	-0.114	0.005761	RPL7L1	3UTR
chr3@194370920@-	0.789	0.286	-0.503	0.005667	LSG1	Intron
chr11@9771768@+	0.145	0.025	-0.12	0.00562	SWAP70	3UTR
chr3@194129022@-	0.364	0	-0.364	0.005593	ATP13A3	Intron
chr13@21949164@-	0.088	0	-0.088	0.005568	Intergenic	Intergenic
chr4@17633827@+	0.222	0	-0.222	0.005517	MED28	3UTR
chr10@74999877@-	0.818	0.125	-0.693	0.005477	DNAJC9	Intron
chr7@74298740@-	0.134	0.028	-0.106	0.005475	STAG3L2	Intron
chr1@204524533@+	0.667	0.143	-0.524	0.00547	MDM4	3UTR
chr16@29681559@+	0.091	0.046	-0.045	0.005433	SPN	3UTR
chr1@21110579@-	0.909	0.167	-0.742	0.005414	HP1BP3	Intron
chrX@73428184@-	0.833	0.091	-0.742	0.005414	RP3-368A4.5	Intron
chr7@74296356@-	0.192	0.042	-0.15	0.00537	STAG3L2	Intron
chr5@34906661@-	0.44	0.05	-0.39	0.005358	RAD1	3UTR
chr4@57326275@+	0	0.019	0.019	0.005257	PAICS	3UTR

chr6@42998961@+	0	0.333	0.333	0.005216	RRP36	Intron
chr17@57279282@+	0.367	0.59	0.223	0.005193	CTD-2510F5.6	Intron
chr6@42905264@+	0.129	0.439	0.31	0.005117	CNPY3	Intron
chr17@62485855@-	0.367	0.108	-0.259	0.005101	POLG2	Intron
chr12@69237509@+	0.2	0.043	-0.157	0.005082	MDM2	3UTR
chr13@21738609@-	0.542	0	-0.542	0.005041	SKA3	Intron
chr17@73095543@+	0.9	0	-0.9	0.004995	SLC16A5	Intron
chr22@23997823@-	1	0.2	-0.8	0.004995	KB-1572G7.2	Intron
chr7@75717767@-	0.1	1	0.9	0.004995	Intergenic	Intergenic
chr2@32535766@+	0.196	0.5	0.304	0.004973	YIPF4	3UTR
chr6@31721686@+	0.183	0.019	-0.164	0.004938	MSH5-SAPCD1	Intron
chr2@217073593@+	1	0.333	-0.667	0.004902	Intergenic	Intergenic
chr11@64506488@-	0.325	0.036	-0.289	0.004849	RASGRP2	Intron
chr20@48561297@+	0.714	0	-0.714	0.004808	RNF114	Intron
chr8@95998231@+	0.714	0	-0.714	0.004808	NDUFAF6	Intron
chr11@17357107@+	1	0	-1	0.004762	NUCB2	Intron
chr3@9858374@+	1	0	-1	0.004762	ARPC4-TTLL3	Intron
chr11@67862624@-	0.5	0	-0.5	0.004682	CHKA	Intron
chr7@152345309@-	0.467	0.048	-0.419	0.004678	XRCC2	3UTR
chr1@155286488@+	0	0.129	0.129	0.004669	FDPS	Intron
chr11@66127691@+	0.875	0	-0.875	0.004662	RP11-867G23.8	Intron
chr19@17389251@+	0.875	0	-0.875	0.004662	CTD-2278I10.6	Intron
chr19@6583866@-	1	0.125	-0.875	0.004662	CD70	Intron
chr8@12881678@+	0.833	0	-0.833	0.004662	KIAA1456	3UTR
chr19@58376896@+	0	0.381	0.381	0.004658	Intergenic	Intergenic
chr7@97597060@-	0.667	0	-0.667	0.0046	Intergenic	Intergenic
chr2@128603150@-	0.733	0.318	-0.415	0.00458	RP5-935K16.1	exon
chr3@121552682@-	0.389	0.086	-0.303	0.004555	IQCB1	Intron
chr1@110909907@+	0	0.462	0.462	0.004555	Intergenic	Intergenic
chr17@57770569@+	1	0	-1	0.004545	CLTC	Intron
chr18@2717364@+	1	0	-1	0.004545	SMCHD1	Intron
chr1@1595048@-	0.294	0	-0.294	0.004514	SLC35E2B	3UTR
chr15@50653018@+	0.406	0.1	-0.306	0.004345	GABPB1-AS1	Intron
chr4@2656020@+	0.833	0.125	-0.708	0.004316	FAM193A	Intron
chr12@24969821@-	0.026	0.002	-0.024	0.004255	BCAT1	3UTR
chr17@5286154@+	0.591	0.143	-0.448	0.004049	RABEP1	Intron

chr19@5912840@+	0.52	0.1	-0.42	0.004043	AC104532.2	Intron
chr14@65400829@+	0.154	0	-0.154	0.004007	CHURC1	3UTR
chr16@88784379@-	0.5	0.217	-0.283	0.003966	PIEZO1	exon
chr1@100611293@+	0.304	0	-0.304	0.003898	TRMT13	Intron
chr12@122439884@-	0.455	0	-0.455	0.00389	Intergenic	Intergenic
chr12@110379911@-	0.667	0	-0.667	0.00387	GIT2	Intron
chr9@4736905@-	0.636	0	-0.636	0.00387	AK3	Intron
chr16@19715610@-	0.421	0	-0.421	0.003758	KNOP1	3UTR
chr12@117136934@+	0.933	0.333	-0.6	0.003745	RP11-497G19.2	Intron
chr10@75537382@+	0	0.5	0.5	0.003666	FUT11	Intron
chrX@48438888@+	0.909	0	-0.909	0.003663	Intergenic	Intergenic
chr11@61617000@+	0.073	0.4	0.327	0.00356	FADS2	Intron
chr10@103570675@-	0.786	0.222	-0.564	0.003559	MGEA5	Intron
chr6@36512563@-	0.342	0.059	-0.283	0.003539	STK38	Intron
chr4@185260395@-	0.122	0.353	0.231	0.003518	Intergenic	Intergenic
chr19@6584433@-	0.444	0	-0.444	0.003504	CD70	Intron
chr19@13884346@+	0.781	0.393	-0.388	0.003464	MRI1	3UTR
chr7@100427794@-	0.833	0	-0.833	0.003394	Intergenic	Intergenic
chrX@73421607@-	0.391	0.138	-0.253	0.003264	RP3-368A4.5	exon
chr17@49042216@-	0.357	0.079	-0.278	0.003259	SPAG9	3UTR
chr12@64803499@+	0.778	0	-0.778	0.003234	XPOT	5UTR
chr7@73646282@-	0.041	0	-0.041	0.003233	RFC2	3UTR
chr3@122333766@+	0	0.5	0.5	0.003122	PARP15	Intron
chr15@66627887@-	0.385	0	-0.385	0.00311	Intergenic	Intergenic
chr22@23992964@-	1	0	-1	0.00303	ASLP1	Intron
chr16@2145299@-	0.111	1	0.889	0.002997	RP11-304L19.3	Intron
chr1@173765073@-	0.833	0	-0.833	0.002997	Intergenic	Intergenic
chr6@99875859@+	1	0.167	-0.833	0.002997	RP11-98I9.4	Intron
chr3@45721989@+	0.294	0.078	-0.216	0.002973	LIMD1	3UTR
chr6@31720288@+	0	0.462	0.462	0.00289	MSH5-SAPCD1	Intron
chr8@48889633@+	0.108	0.047	-0.061	0.002814	MCM4	3UTR
chr1@1419690@+	0.588	0.067	-0.521	0.002763	ATAD3B	exon
chr10@73994762@+	0.513	0.161	-0.352	0.002727	ANAPC16	3UTR
chr1@28144656@+	0.643	0	-0.643	0.002605	STX12	Intron
chr17@26932730@-	0.462	0.05	-0.412	0.00257	RP11-192H23.4	Intron
chr16@11025557@+	0.167	0	-0.167	0.002555	CIITA	Intron
chrX@48436419@+	0	0.016	0.016	0.002545	RBM3	3UTR

chr17@10576406@-	0.375	0.952	0.577	0.002535	CTC-297N7.8	exon
chr5@34906737@-	0.514	0.12	-0.394	0.002467	RAD1	3UTR
chr7@65996065@-	0.714	0	-0.714	0.002451	GS1-124K5.11	exon
chr3@44537773@-	0.75	0.182	-0.568	0.002383	Intergenic	Intergenic
chr3@121552007@-	0.03	0.323	0.293	0.002342	IQCB1	Intron
chr19@53121832@-	0.231	0	-0.231	0.002324	ZNF83	exon
chr16@11018329@+	0.053	0	-0.053	0.002279	CIITA	3UTR
chr11@3841723@+	0.778	0	-0.778	0.002262	AC090587.2	exon
chr16@89834947@-	0.75	0	-0.75	0.002262	FANCA	Intron
chr16@89834964@-	0.778	0	-0.778	0.002262	FANCA	Intron
chr7@99748759@+	0.778	0	-0.778	0.002262	LAMTOR4	Intron
chr16@11025657@+	0.045	0.412	0.367	0.002254	CIITA	Intron
chr16@592641@+	0.64	0.373	-0.267	0.002213	CAPN15	Intron
chr3@50149654@+	0.533	0	-0.533	0.002199	RBM5	Intron
chr20@3849206@+	0.273	0.026	-0.247	0.002192	MAVS	3UTR
chr19@46216833@-	0.235	0.023	-0.212	0.002191	FBXO46	Intron
chr19@48638507@-	0.667	0	-0.667	0.002167	LIG1	Intron
chr2@96856501@-	1	0	-1	0.002165	STARD7	Intron
chr13@25891637@+	1	0.375	-0.625	0.002127	NUPL1	Intron
chr2@75896968@+	0.5	0	-0.5	0.002122	MRPL19	Intron
chrX@77083041@-	0.2	0.5	0.3	0.002066	MAGT1	3UTR
chr3@49047939@+	0.281	0	-0.281	0.002037	WDR6	Intron
chr19@49492141@-	1	0	-1	0.00202	GYS1	Intron
chr1@44934509@+	1	0	-1	0.00202	RNF220	Intron
chr13@111552341@-	0.568	0.325	-0.243	0.002012	ANKRD10	Intron
chr22@47067626@+	1	0.167	-0.833	0.001998	GRAMD4	Intron
chr10@51733181@+	0.316	0	-0.316	0.00194	LINC00843	Intron
chr16@22520632@+	0.517	0.125	-0.392	0.001883	NPIPB5	Intron
chr7@139055410@+	0	0.2	0.2	0.00183	LUC7L2	Intron
chr1@85398063@-	0.35	0.077	-0.273	0.001775	MCOLN2	Intron
chrX@73428789@-	0.929	0.333	-0.596	0.001699	RP3-368A4.5	Intron
chr6@31718821@+	0.32	0	-0.32	0.001699	MSH5-SAPCD1	Intron
chr15@64654629@-	0.615	0	-0.615	0.001648	CTD-2116N17.1	Intron
chrX@30741826@+	0	0.8	0.8	0.001634	GK	Intron
chr5@172357620@+	0.5	0	-0.5	0.001556	ERGIC1	Intron
chr2@128602366@-	0.765	0.188	-0.577	0.001555	RP5-935K16.1	exon

chr1@1662547@-	0.277	0.056	-0.221	0.001549	RP1-283E3.8	Intron
chr13@111552356@-	0.31	0.097	-0.213	0.001456	ANKRD10	Intron
chr19@58378232@+	0.324	0	-0.324	0.001435	Intergenic	Intergenic
chr11@65540353@-	1	0	-1	0.001399	Intergenic	Intergenic
chr16@66921776@+	1	0.143	-0.857	0.001399	PDP2	3UTR
chr3@185354141@-	1	0	-1	0.001399	Intergenic	Intergenic
chr8@145644293@+	0.889	0	-0.889	0.001399	Intergenic	Intergenic
chr7@99081203@+	0.304	0	-0.304	0.001384	ZNF789	Intron
chr21@34637098@+	0	0.333	0.333	0.00137	AP000295.9	Intron
chr16@11018290@+	0.165	0.045	-0.12	0.001352	CIITA	3UTR
chr20@34217719@-	0.579	0	-0.579	0.001352	CPNE1	Intron
chr7@102284947@-	0.636	0	-0.636	0.001346	POLR2J2	Intron
chr22@47067686@+	1	0.444	-0.556	0.001283	GRAMD4	Intron
chr5@180614307@-	0	1	1	0.001263	Intergenic	Intergenic
chr6@42047554@+	0.138	0	-0.138	0.00126	TAF8	3UTR
chr16@29679978@+	0.01	0.08	0.07	0.001214	SPN	3UTR
chr1@46075604@+	0.352	0	-0.352	0.001143	NASP	exon
chr7@75044166@-	0.071	0.01	-0.061	0.001133	Intergenic	Intergenic
chr2@198357189@-	0	0.214	0.214	0.001083	HSPD1	Intron
chr16@89586934@+	0.028	0.31	0.282	0.001048	SPG7	Intron
chr4@2940164@-	0.203	0.016	-0.187	0.001022	NOP14	3UTR
chr7@74298734@-	0.681	0.453	-0.228	0.000996	STAG3L2	Intron
chr16@57090342@+	0.947	0.513	-0.434	0.000994	NLRC5	Intron
chr2@96481129@-	0.279	0.045	-0.234	0.000992	LINC00342	exon
chr14@50066850@+	0.873	0.612	-0.261	0.000963	RHOQP1	exon
chr14@58740375@+	0.241	0	-0.241	0.000958	Intergenic	Intergenic
chr17@5327686@+	0.157	0.516	0.359	0.000936	RPAIN	Intron
chr8@146051203@-	0.316	0.929	0.613	0.000928	Intergenic	Intergenic
chr4@185262119@-	0.044	0.131	0.087	0.000874	RP11-290F5.2	exon
chr3@195368757@+	1	0.059	-0.941	0.000835	Intergenic	Intergenic
chr2@96496466@-	0.812	0.238	-0.574	0.00083	Intergenic	Intergenic
chr19@13884419@+	0.64	0.136	-0.504	0.000824	MRI1	3UTR
chr19@53382117@-	0.22	0.041	-0.179	0.00081	ZNF320	3UTR
chr9@136035124@-	0.486	0.08	-0.406	0.000796	RALGDS	Intron
chr4@119221257@-	1	0.4	-0.6	0.0007	PRSS12	Intron
chr2@198356725@-	0.345	0.065	-0.28	0.000679	HSPD1	Intron
chr19@12740516@+	0	0.375	0.375	0.000653	ZNF791	3UTR
chr16@22508517@+	0.909	0.25	-0.659	0.000635	NPIPB5	Intron
chr6@42856042@+	0.007	0.107	0.1	0.000627	RPL7L1	3UTR
chr19@12009442@+	1	0	-1	0.000583	ZNF69	Intron

chr12@25202487@+	0.421	0.085	-0.336	0.000567	LRMP	Intron
chr17@76203545@+	0	0.138	0.138	0.000527	AFMID	3UTR
chr15@65251819@-	0.75	0	-0.75	0.000516	Intergenic	Intergenic
chr17@4929924@+	1	0	-1	0.0005	KIF1C	3UTR
chr7@128301346@+	0.293	0.079	-0.214	0.000485	Intergenic	Intergenic
chr6@35805924@-	0.815	0.345	-0.47	0.00048	SRPK1	Intron
chr16@29681699@+	0.117	0.03	-0.087	0.00048	SPN	3UTR
chr16@29678950@+	0.03	0	-0.03	0.000472	SPN	3UTR
chr20@35410369@-	0.714	0.15	-0.564	0.000433	SOGA1	3UTR
chr16@21877807@-	0.391	0.658	0.267	0.00042	NPIPB4	Intron
chr5@79923329@-	0.215	0.1	-0.115	0.000414	DHFR	3UTR
chr12@121752090@-	0.9	0	-0.9	0.000411	ANAPC5	Intron
chr16@89586884@+	0.364	0.077	-0.287	0.00041	SPG7	Intron
chr4@88968440@+	0.875	0.111	-0.764	0.000397	PKD2	Intron
chr14@53243394@-	0.714	0.364	-0.35	0.000392	GNPNAT1	3UTR
chr16@89835007@-	0.769	0	-0.769	0.000364	FANCA	Intron
chr20@3851763@+	0.214	0	-0.214	0.000333	Intergenic	Intergenic
chr19@16747552@-	0.833	0.238	-0.595	0.000328	CTC-429P9.4	Intron
chr2@86249840@-	0.03	0.414	0.384	0.000283	POLR1A	3UTR
chr17@29223763@+	0.533	0	-0.533	0.000273	Intergenic	Intergenic
chr17@42256868@-	0.923	0	-0.923	0.000258	ASB16-AS1	Intron
chr19@58357294@+	0.467	0	-0.467	0.000239	ZNF587B	3UTR
chr12@123244067@+	1	0	-1	0.000229	DENR	Intron
chr14@31351049@+	1	0	-1	0.000229	COCH	Intron
chr22@41327595@+	1	0.182	-0.818	0.000221	XPNPEP3	3UTR
chr16@29679886@+	0.371	0.23	-0.141	0.000194	SPN	3UTR
chr7@74297433@-	0	0.101	0.101	0.000174	STAG3L2	Intron
chr13@21738550@-	0.8	0	-0.8	0.000159	SKA3	Intron
chr19@1459302@-	0.826	0.1	-0.726	0.000158	CTB-25B13.12	Intron
chr11@64506044@-	0.926	0.448	-0.478	0.000145	RASGRP2	Intron
chr12@123243223@+	0.515	0.043	-0.472	0.000145	DENR	Intron
chr1@1252441@-	0.744	0.227	-0.517	0.000137	CPSF3L	exon
chr7@152342999@-	0.87	0.613	-0.257	0.000135	XRCC2	exon
chr12@25201081@+	0.333	0.048	-0.285	0.000127	LRMP	Intron
chr8@41400029@+	0.349	0	-0.349	7.56E-05	GINS4	3UTR
chrX@73421601@-	0.217	0	-0.217	7.20E-05	RP3-368A4.5	exon
chr19@38807560@-	0.8	0	-0.8	7.14E-05	YIF1B	Intron
chr15@90618748@-	0.27	0	-0.27	6.36E-05	RP11-617F23.1	Intron

chr6@31718813@+	0.72	0.154	-0.566	5.93E-05	MSH5-SAPCD1	Intron
chr7@102189501@-	0.778	0.1	-0.678	3.96E-05	RP11-514P8.7	Intron
chr8@52735902@-	0.812	0.067	-0.745	3.85E-05	PCMTD1	Intron
chr4@17802955@-	0	0.239	0.239	2.71E-05	DCAF16	3UTR
chr6@109641808@+	1	0	-1	2.29E-05	CCDC162P	Intron
chr2@37327590@-	0.134	0	-0.134	7.04E-06	EIF2AK2	3UTR
chr7@44841489@+	0.771	0.389	-0.382	4.14E-06	PPIA	3UTR
chr12@98942885@+	0.607	0.27	-0.337	2.25E-07	TMPO	3UTR
chr1@1661033@-	0	0.321	0.321	7.59E-08	RP1-283E3.8	Intron
