# Supplementary material for: The snoRNA-like lncRNA LNC-SNO49AB drives leukemia by activating the RNA-editing enzyme ADAR1
Source: Cell Discov. 2022 Nov 1;8:117. doi: 10.1038/s41421-022-00460-9 (PMC9622897; doi:10.1038/s41421-022-00460-9)
Supplement: Supplementary file 11 — Supplemental Tab S4 [file 41421_2022_460_MOESM11_ESM.pdf]

Supplementary Table S4 List of the dysregulated genes of LNC-SNO49AB knockdown.

gene_name	si-LNC-SNO49AB_FPKM	NC_FPKM	FDR	log2FC	regulated
HECW1	0.701549	0.093827	0.001523	1.278972	up
LASP1	9.104875	25.17236	0	-1.66633	down
CASP10	3.50389	1.956379	3.78E-10	1.06284	up
CFLAR	4.113246	6.483619	9.55E-15	1.094915	up
ARX	0.34718	1.030269	9.12E-07	-1.54014	down
NOS2	0.495156	0.098721	6.55E-09	2.200268	up
BAIAP3	0.43224	2.010235	5.74E-14	-1.63151	down
CRAMP1	3.312109	0.117597	0	4.388777	up
SLC6A13	0.457129	2.287902	2.77E-10	-1.92496	down
NME1-NME2	11.984322	0	0	8.713988	up
RUNX3	0.245766	0.579383	0.001683	-1.20535	down
NRXN3	0.418633	0.098796	3.68E-08	1.872123	up
STAP1	6.916653	2.031216	0	1.72025	up
MFAP3	4.968895	8.507713	0	-2.27601	down
RRM2B	22.248684	10.56899	0	1.120413	up
CELF2	52.942091	25.00839	0	1.199516	up
PPP1R3F	0.363432	0.710013	5.18E-06	-1.62683	down
MSH4	1.771253	0.82063	4.44E-16	1.414437	up
WNT8A	1.398702	0.363437	1.25E-06	1.84517	up
BORCS8-MEF2B	5.425428	1.367325	0	2.778051	up
KDM4A	17.660991	6.214681	0	1.691328	up
SLC9A3	0.820491	1.621252	3.23E-08	-1.08197	down
STON1-GTF2A1L	1.177783	0.020588	0	5.865539	up
HES2	0.053931	0.246765	0.004078	-1.9382	down
VASH1	2.212453	3.846899	1.27E-11	-1.07111	down
ADGRL1	2.076966	4.207432	0	-1.22385	down
ST6GAL1	26.428909	73.45811	0	-1.59619	down
MRPS34	24.37294	69.76111	0	-1.48806	down
VPS9D1	1.41162	3.409028	0	-1.67619	down
FGFR1	0.458799	0.18119	2.27E-05	1.365538	up
ACER3	2.127547	3.436938	0	-1.87669	down
TULP3	3.489895	7.529976	2.42E-14	-1.18059	down
ZNF264	1.416151	0.01	0	6.452313	up
EFR3B	0.749606	0.73253	3.46E-06	1.095773	up
SCAMP1	6.964915	13.13749	1.33E-15	-1.09486	down
NID2	1.298347	0.629222	0.00062	1.037812	up
TFAP2C	0.060952	0.441082	1.59E-06	-2.60547	down
CMTM1	3.558719	0.490344	0	2.939024	up

BIRC5	28.322301	15.39641	9.5E-14	1.000724	up
PCBP4	13.213572	5.462898	0	1.300631	up
LYZ	0.860128	2.36364	1.07E-07	-1.50071	down
MCOLN1	1.520567	4.652848	0	-1.6979	down
WHRN	0.333163	0.071871	0.002069	1.927059	up
BLNK	6.357911	3.095787	3.85E-09	1.068926	up
MZF1	14.699026	7.789267	1.11E-15	1.055321	up
GGT1	2.430008	0.883161	5.92E-14	1.548046	up
POLDIP3	20.101732	39.32656	2.89E-14	-1.00677	down
APOL1	2.906876	1.23886	1.93E-06	1.139918	up
GRAP2	0.371341	0.18767	3.48E-07	2.433746	up
KIAA0391	7.024535	4.768055	0	1.719584	up
NINL	0.450881	1.633325	0	-1.83006	down
SLA2	0.229438	0.628334	0.003905	-1.40401	down
STK4	14.719425	20.66661	0	-1.64964	down
SALL4	0.251716	0.957166	2.54E-09	-1.31688	down
ADNP	23.062528	61.15743	0	-1.36645	down
RASSF2	32.954231	9.125558	0	1.866844	up
DHRS12	2.187537	0.85681	0.008329	1.018827	up
NME3	20.181521	8.04831	0	1.604056	up
MON1B	8.223415	15.20098	0	-2.81594	down
CORO7-PAM16	11.206891	18.39051	4.06E-14	-1.06738	down
GABPB1	19.010388	13.27992	1.22E-15	1.028477	up
TJP1	0.613668	0.0408	1.88E-10	2.513031	up
BLOC1S6	26.274721	7.597299	0	2.546186	up
NCALD	1.567692	0.947531	0.004154	1.036304	up
LEPROTL1	19.597902	6.836068	0	1.718972	up
TUBB4A	1.342679	3.060776	1.48E-07	-1.10778	down
TLE6	0.204608	0.84095	0.006695	-1.55027	down
ZNF419	6.52091	2.369552	3.79E-13	1.277961	up
CD33	0.377394	1.665773	4.42E-08	-2.02178	down
ATP1A3	8.401678	20.1489	0	-1.26683	down
PIK3R2	0.321068	0.605009	3.47E-05	-1.72555	down
ERF	9.736235	5.03699	8.88E-16	1.142152	up
GTPBP10	7.49377	3.937581	5.63E-14	1.052732	up
RASA4	1.179059	0.252361	1.48E-06	1.615966	up
PTCD1	1.246208	3.475558	0	-1.55179	down
ZKSCAN1	22.316404	8.002505	0	1.471573	up
PCOLCE	4.957168	2.392986	5.86E-14	1.371088	up
FSD1L	2.588759	2.166625	2.34E-13	-1.22786	down
DNTT	20.482347	6.055174	0	1.758117	up
PLEKHA1	1.023809	0.349803	1.19E-11	1.775366	up
TSPAN14	4.896591	9.319138	0	-1.38471	down
B9D1	0.608947	1.58543	0.004874	-1.5986	down

PRKAR1A	77.410416	77.48938	6.66E-16	-1.04816	down
PMP22	0.278477	2.342641	6.88E-15	-2.49191	down
CLCN3	20.31985	13.15497	0	1.144396	up
CYP27B1	0.95494	2.029507	0.000173	-1.05935	down
ELK3	7.176916	2.093285	0	2.008118	up
GPN3	11.190348	19.15218	2.26E-12	-1.04972	down
NOP2	9.939134	1.0849	0	3.149779	up
MAPK14	19.798692	11.18121	1.11E-16	1.093768	up
KCTD20	37.47976	11.72382	0	1.823802	up
PHACTR1	19.332409	14.61843	0	-1.16342	down
SLC16A10	0.525757	0.714384	1.07E-05	-1.4613	down
POLR3G	4.712763	8.620222	0	-1.40632	down
PCDH12	0.793434	0.38282	2.35E-05	1.044732	up
SMAD5	14.208441	23.63141	0	-1.26613	down
PLCH1	0.518095	0.083819	2.62E-06	2.286761	up
PDE1A	0.542154	0.221476	0.00128	1.197652	up
APC2	1.35831	3.930356	0	-1.42964	down
LGALS8	11.930852	4.429464	0	1.322218	up
RCAN3	3.701533	1.093103	0	2.372121	up
RNF146	9.286098	3.321872	0	1.578604	up
MYL12B	124.79442	39.07579	0	1.68911	up
TJP2	2.136587	0.877325	1.95E-09	1.138656	up
HSDL2	4.780163	5.901759	4.88E-14	1.060415	up
KCNIP2	1.092737	0.565718	5.73E-08	2.062352	up
NUP43	9.529816	13.59034	0	-1.12247	down
PYROXD1	7.134865	3.757211	0	1.532503	up
SARDH	0.522304	1.686422	2.38E-06	-1.25555	down
CSE1L	27.733027	59.75824	0	-1.10758	down
STX16	4.915996	23.2057	0	-3.02903	down
SNRNP27	5.513241	16.67194	0	-1.94743	down
CDKN1A	16.45999	34.45043	4.44E-16	-1.08995	down
RUNX2	11.399068	5.239198	1.41E-13	1.013416	up
AHNAK	0.376692	0.088241	2.37E-05	1.188258	up
EMG1	3.070699	8.573193	0	-1.6122	down
SLC10A3	3.651747	1.551723	8.25E-09	1.219749	up
HNRNPH2	0.618271	29.83131	0	-5.54106	down
HELB	1.432257	1.390742	0	1.642243	up
TUBA4A	21.874303	7.144595	0	1.59511	up
GNG11	0.798535	0.262561	8.27E-06	1.550935	up
GNAI1	1.633835	0.600426	0	2.025267	up
CALU	10.91892	26.36377	0	-1.34889	down
MYO5C	0.566703	0.25572	3.65E-05	1.146689	up
MINDY2	7.881468	4.076803	1.33E-15	1.074993	up
CALML4	4.050519	1.874731	6.52E-08	1.266973	up

PUDP	7.450058	19.11619	0	-1.45782	down
KIF1A	0.221341	1.043012	5.72E-05	-2.74665	down
TRPM4	0.137972	0.578982	4.54E-07	-2.0564	down
PPAN	16.142866	43.65965	0	-1.45124	down
AKAP12	0.394972	0.048461	1.25E-13	2.533259	up
GGT7	0.526534	1.26735	1.16E-05	-1.14943	down
ZNF227	10.331993	4.898981	2.05E-12	1.035682	up
ZNF428	2.059764	5.401307	2.03E-05	-1.02272	down
SCLY	8.106649	15.90936	0	-1.37543	down
REEP2	0.412956	1.267809	0.000385	-1.3585	down
DCAF8	37.720415	18.2324	0	1.985487	up
RBM38	23.380313	12.63049	0	1.194724	up
WNT2B	0.109697	0.21785	2.28E-13	-2.77204	down
EMP1	0.677578	0.311679	6.28E-05	1.115448	up
APC	12.228453	5.079636	0	1.106062	up
CEMIP2	3.888425	1.801926	6.88E-15	1.185331	up
KHDC1	1.397143	3.7942	0	-2.87393	down
ZC3H10	0.87282	2.831513	0	-1.96033	down
MPHOSPH6	7.333451	15.01235	0	-1.37549	down
KIAA0513	0.594355	0.144719	5.72E-06	1.42528	up
CCDC102A	1.614208	2.657993	5.77E-06	-1.03592	down
STX6	14.491844	4.561343	0	1.664756	up
TMBIM1	16.112276	5.957475	1.67E-15	1.196141	up
THSD1	0.759908	0.164212	1.18E-09	2.115617	up
MTHFS	5.212661	10.25086	3.87E-07	-1.00571	down
IL1RN	0.955513	1.731542	0.002887	-1.23851	down
MYC	98.597976	13.48599	0	3.76726	up
TUBB2B	0.417263	1.156271	1.78E-06	-1.8286	down
ETFBKMT	0.339039	0.27252	8.08E-11	2.496517	up
TMBIM6	126.054982	52.55252	0	1.335075	up
HNRNPA1L2	3.368872	11.95502	0	-1.81793	down
LPAR6	0.02701	5.379733	0	-7.02788	down
RHOF	16.873184	7.434494	0	1.105806	up
TCF12	51.022677	152.4349	0	-1.33649	down
DET1	1.015533	2.728345	1.4E-08	-1.27742	down
GNAL	0.351872	0.150991	0	3.740146	up
TMEM91	2.787632	1.265772	1.77E-06	1.316205	up
CNKSR1	1.624742	2.6107	1.94E-06	-1.08593	down
MAP3K6	0.806577	1.916364	2.86E-06	-1.00197	down
ETNK2	0.632827	1.328844	0.002966	-1.0287	down
RAB5A	17.779364	8.791692	0	1.184393	up
RBMS3	0.181387	0.04737	6.6E-05	1.852755	up
CTDSPL	1.231196	0.548207	0.004513	1.027426	up
AC073896.1	9.803782	0	0	7.588185	up

LIMD1	9.050087	12.03828	0	-1.16822	down
CISD2	22.328914	2.431733	0	1.917339	up
TNFAIP8	6.125074	12.19901	2.94E-14	-1.16158	down
ARMT1	13.231592	5.723391	0	1.24797	up
PURB	2.671225	6.263201	0	-1.22759	down
AGBL3	0.214355	0.963926	1.67E-05	-1.61328	down
ZNF182	3.702534	0.566772	0	2.648136	up
CSGALNACT1	2.292938	1.12581	3.77E-10	1.337025	up
HMBOX1	3.571091	5.018676	0	-1.14941	down
STOM	5.853586	10.08679	1.33E-15	-1.18683	down
SURF4	31.206065	12.47439	0	1.375197	up
CNNM2	1.205263	1.189544	8.94E-11	-1.18405	down
MRPL49	19.112665	8.376782	0	1.292778	up
TM7SF2	2.170747	8.350754	0	-2.11017	down
PIH1D2	0.755149	0.203419	0.001163	1.916603	up
TIMM8B	29.431884	12.00781	2.34E-14	1.128929	up
KCNA6	0.276213	0.067528	0.001609	1.854336	up
DIP2C	0.223324	0.067068	0.0002	1.623939	up
QTRT2	13.854166	6.790978	4.91E-14	1.031303	up
PABPC3	1.068832	0.499025	0.000398	1.075419	up
BANK1	2.143738	0.712024	5.68E-14	1.54526	up
BCL2L11	9.493972	3.10278	0	2.274847	up
DGKE	19.044639	10.12501	0	1.358988	up
IMPACT	0.271053	0.059225	0.002324	1.969693	up
ROBO4	0.948537	0.345986	1.16E-08	1.426252	up
PIEZO2	0.337937	0.066219	1.92E-14	2.401227	up
APOOL	6.255023	8.603798	0	-1.335	down
CABP1	0.415126	1.01903	4.52E-09	-1.95378	down
FAM86C1	3.014981	1.869015	3.1E-07	1.1489	up
NBL1	1.133532	2.485208	2.52E-07	-1.2352	down
UBE2Z	31.725875	11.11183	0	1.615112	up
SPON2	3.170218	5.665931	0	-1.6241	down
CCDC117	4.874863	23.79652	0	-2.66688	down
ZNF222	1.124297	2.539722	1.45E-05	-1.22168	down
NPR2	1.56545	1.172543	0	1.49104	up
ZNF233	0.10617	1.077324	4.44E-16	-3.14325	down
CALM3	53.111046	136.3177	0	-1.38464	down
ANKLE1	4.209483	20.35466	0	-2.39451	down
SCIMP	2.774632	0.836456	4.24E-14	1.671067	up
CCDC78	2.078858	4.556462	3.11E-07	-1.02527	down
NEU3	3.128497	10.68578	0	-2.11215	down
FLVCR1	6.078223	3.529584	1.57E-14	1.084484	up
COL6A3	0.607771	0.27472	8.82E-06	1.033631	up
EIF4E3	0.210834	0.077872	0.000723	1.371239	up

SERPINI1	1.359304	0.612718	0.001004	1.224821	up
SPTA1	1.024967	0.33895	3.03E-09	1.446075	up
ATXN7	0.259025	0.909676	6.75E-14	-1.77062	down
PTX3	0.509551	0.099979	0.001067	2.136644	up
RHOBTB3	13.946279	28.81022	0	-1.30343	down
CFAP97	37.481758	47.49072	3.89E-15	-1.03101	down
SAP30L	3.170493	6.349561	2.29E-14	-1.08732	down
RELL2	0.680099	1.933763	4.57E-10	-1.54785	down
ZNF12	5.57407	3.375977	1.16E-13	1.094744	up
PEX2	5.991166	13.43366	0	-1.21506	down
BAALC	0.633622	0.104211	1.25E-11	2.96057	up
NIPSNAP3B	6.965355	5.147752	2E-15	1.108083	up
ABCA1	2.279769	0.555649	1.83E-11	1.15453	up
DDIAS	2.051709	5.602292	0	-1.82063	down
CACNB2	0.315815	0.572612	7.01E-10	-1.99165	down
TCP11L2	3.931446	1.796004	4.18E-07	1.047317	up
AMN	1.24978	5.55473	0	-2.10432	down
FRS2	7.121235	4.836725	0	-1.45582	down
ZMAT1	5.161289	1.986122	0	1.447007	up
FAM111A	17.586268	7.253262	0	1.144262	up
ANKDD1A	0.524939	1.590719	3.36E-07	-1.38161	down
MS4A14	0.282196	0	3.15E-10	4.945988	up
FBXO22	4.688079	5.154276	0	1.575708	up
FN3K	0.780846	1.256544	0	-1.75289	down
PRRT2	0.364477	2.971852	0	-3.46929	down
ZNF23	0.978062	3.743402	0	-1.72605	down
TPM4	254.721958	122.6826	0	1.097039	up
CORO6	1.485563	3.236699	2.64E-11	-1.16417	down
TUBA1A	60.212573	132.9824	5.55E-16	-1.05401	down
GPT	0.963354	0.040975	1.78E-15	3.972025	up
KRT80	0.374739	0.810635	0.001401	-1.08958	down
CD320	4.946762	23.19642	0	-2.23864	down
GNG4	0.945065	1.554198	1.22E-09	-1.28751	down
POLR2J3	0.226739	5.807581	0	-3.52461	down
HEXIM2	4.824414	8.646251	9.84E-08	-1.05938	down
LRRC28	5.294388	3.968063	4.69E-14	1.192409	up
ZNF608	4.729168	2.061168	0	1.278352	up
TMEM129	10.630685	4.120621	0	1.594564	up
CCDC126	1.4309	0.869669	7.84E-06	1.342293	up
CD52	33.473637	8.245491	0	2.006979	up
HIC2	2.328713	5.354782	0	-1.197	down
GP9	4.319128	9.91064	1.98E-09	-1.17622	down
RAC3	2.705876	5.45745	1.14E-06	-1.11895	down
LIMS1	19.485163	5.053385	0	2.638687	up

SPATA24	3.648362	1.581943	1.5E-07	1.558503	up
RASA4B	1.189313	0.003078	0	5.983059	up
GSTA4	4.275335	1.679843	8.98E-09	1.305021	up
S1PR1	0.732189	0.231632	3.63E-05	1.576093	up
COL24A1	0.209272	1.084412	1.5E-05	-1.29561	down
PLEKHG5	0.392382	0.892603	0.000176	-1.00128	down
RRM2	38.955555	12.39038	0	1.789295	up
PRNP	8.957197	28.35112	0	-1.97624	down
SRGAP2C	4.828483	1.763963	0	1.862656	up
DRC3	1.522262	1.097465	0	2.275556	up
SMN1	35.547977	12.23764	0	1.650804	up
MACROD2	1.117142	0.403522	0	2.613154	up
GNG12	0.927421	0.33257	3.26E-08	1.443284	up
ZNF24	28.296808	4.904228	0	2.566748	up
CTSW	0.390814	1.850443	5.93E-10	-2.16909	down
EFEMP2	0.258855	1.308204	6.74E-08	-2.06523	down
ZNF596	0.402614	0.814237	4.81E-08	-2.1442	down
DCP2	25.909488	8.562236	0	2.112726	up
PDP2	7.57613	3.941777	0	1.309775	up
LCLAT1	3.727998	11.7644	0	-1.85535	down
AC097637.1	0	0.321488	3.73E-12	-5.22449	down
SPDYE2B	1.142276	0	0	8.331497	up
HAP1	3.086679	4.367325	9.77E-15	-1.43712	down
TIGD3	0.478452	1.268922	0.000156	-1.35247	down
RGMB	0.820892	0.791741	0.000958	1.053083	up
PITPNA	4.059761	1.362132	0	3.098106	up
ZNF497	0.361046	0.140293	0.000911	1.848845	up
IQCK	0.650568	0.522097	6.49E-07	1.807781	up
GAPT	1.830955	0.665568	7.3E-05	1.246453	up
AMIGO3	0.161483	4.17693	0	-4.551	down
ZNF613	1.759811	2.159666	7.36E-10	-1.29378	down
TPRN	5.416266	14.85595	0	-1.28738	down
B3GNT4	0.107442	0.607775	1.1E-06	-2.17709	down
EID2B	0.200697	1.535715	4.44E-16	-2.79181	down
RNF152	7.279046	3.591263	0	1.144405	up
CDK5R1	2.634254	1.057412	1.04E-13	1.305979	up
BASP1	7.027015	9.176152	1.71E-14	-1.23345	down
IRX5	0.668215	1.416796	0.001788	-1.0409	down
PXMP2	5.655135	14.02991	0	-1.51755	down
RIMKLA	0.106623	0.178664	0.000465	-1.67556	down
MIEF2	1.160245	3.681212	0	-2.71925	down
SLC25A22	9.845085	20.18929	0	-1.27413	down
MAMDC4	2.2323	4.37575	6.63E-14	-1.10577	down
GPR150	0.839592	0.061608	7.57E-13	3.336525	up

WDR6	22.675838	51.20417	0	-1.11699	down
ZNF223	0.215934	0.768258	3.26E-06	-2.04558	down
ERFE	1.303044	1.503497	3.8E-05	-1.13874	down
APOLD1	26.947924	9.630404	0	1.348265	up
RTL3	0.154496	0.560837	0.004903	-1.62891	down
PCED1B	4.278031	2.245123	1.68E-10	1.223278	up
AKAP5	0.362972	0.509185	0.003529	-1.21767	down
ZNF48	2.095671	5.296794	0	-2.02715	down
EIF2S3B	0.618384	4.567452	0	-2.82696	down
CXCR2	0.682542	0.325797	4.8E-05	2.143429	up
FKRP	5.019988	2.908109	0	1.680948	up
TRAPPC5	1.999494	9.396033	0	-1.66762	down
DDN	0.765283	1.675252	6.21E-07	-1.12054	down
SGSH	0.566984	1.225877	0.000102	-1.08244	down
TMIE	1.860449	3.805122	5.1E-07	-1.0326	down
SLX1B	0.492626	14.49881	0	-5.41448	down
YIPF6	5.075919	1.157046	0	2.175151	up
RFX7	2.080046	9.352482	0	-2.14732	down
CMC4	6.445933	2.482388	2.25E-09	1.281152	up
CAMK1D	11.505743	5.862733	0	1.992179	up
SELENOF	68.056594	21.86376	0	1.787841	up
FHL3	0.324698	1.548322	0.004747	-1.54453	down
TREX2	2.745052	8.01111	0	-1.86145	down
DIABLO	9.826517	22.8448	0	-1.28345	down
VPS33B	0.045265	1.033671	0	-3.88472	down
C22orf46	1.0706	2.266113	4.29E-09	-1.07191	down
CCR10	2.161324	0.800903	1.11E-06	1.309176	up
KRBA2	0.464873	2.160932	0	-2.18092	down
HIST2H2BE	3.2686	1.593272	1.52E-06	1.027122	up
44444	2.493356	5.514873	0	-2.23742	down
TMEM186	3.994204	0.614235	0	2.654624	up
TMEM121	0.233336	1.499395	9.54E-09	-1.97359	down
BRI3BP	12.373144	25.53802	0	-1.14632	down
MRPL30	1.399073	2.15644	2.2E-07	-1.29873	down
METTL7A	18.351756	5.202777	0	1.891654	up
SP1	14.375761	25.75043	0	-2.20417	down
PDIA2	0.052989	0.557308	1.36E-05	-2.99003	down
CLCNKA	0.219485	0.635238	0.004311	-1.45672	down
PPARA	2.536112	3.555556	0	-1.561	down
ZNF546	1.03591	1.503354	0	-3.25879	down
PCDHB13	0	0.725389	0	-6.81544	down
VMAC	3.113983	0.063846	0	5.176685	up
NHEJ1	2.043164	11.86763	0	-2.79155	down
ZNF70	3.594106	1.285602	8.88E-16	1.260299	up

PLSCR3	3.177403	7.037709	0	-1.66894	down
NCR3LG1	0.220475	0.511042	0.000169	-1.18367	down
ZNF383	2.939719	0.990664	0	2.078698	up
CNR2	0.077493	0.322164	7.79E-06	-1.9694	down
GRAPL	0.216559	1.610368	5.62E-06	-2.19993	down
S100A5	1.716813	0.119613	1.08E-06	3.010356	up
ZNF569	4.781138	2.677316	4.02E-14	1.190052	up
ZNF605	0.565628	1.187581	6.96E-11	-1.2721	down
IPO4	1.479694	14.42467	0	-3.38211	down
ZNF700	9.274023	5.888148	0	1.404978	up
ZNF100	6.007227	10.8161	0	-1.16656	down
ZNF763	1.65318	0.961425	0.001807	1.053801	up
ZGPAT	0.840151	2.287358	2.74E-08	-1.42413	down
ZNF98	0	0.906128	0	-7.71051	down
FBXL22	0.116386	0.428709	0.003017	-2.16507	down
SSPO	0.00583	0.067897	6.61E-07	-3.1579	down
RAB40C	7.546347	4.209561	0	1.391162	up
ZNF461	2.468955	1.552156	1.96E-12	1.213071	up
ZNF607	4.763156	0.824411	0	2.648482	up
ZNF583	1.023175	0.729077	5.42E-13	2.341024	up
ZNF28	1.982055	3.487555	0	-2.82868	down
SLC34A3	0	0.520562	2.03E-11	-5.15126	down
CIPC	7.350651	1.797994	0	2.130525	up
ZNF525	4.908024	7.737372	0	-1.1892	down
LCN8	22.782582	6.438585	0	1.834399	up
MPIG6B	2.88135	2.039141	2.36E-09	1.39597	up
LST1	2.409064	0.587754	0.000181	1.745748	up
ZNF814	3.112825	1.859447	0	1.595404	up
ZNF805	0.261941	1.193394	1.03E-09	-2.07045	down
MROH6	2.249975	4.405781	3.86E-11	-1.01002	down
ZNF155	0.328339	1.215865	3.56E-12	-1.75494	down
SYCE1L	5.541697	1.914689	0	1.674915	up
SAP25	3.454046	1.073357	8.01E-09	1.610361	up
CCDC85C	0.381849	1.90321	0	-2.34572	down
SMN2	11.847554	36.70913	0	-1.59422	down
XKR4	0.232128	0.069244	2.16E-12	1.723499	up
HAUS7	13.311476	6.559271	4.4E-11	1.085159	up
DDX47	5.134242	37.65773	0	-2.86964	down
LTB4R2	0.363542	2.056012	0	-2.68349	down
MEF2B	0.030035	1.73059	0	-4.9855	down
RPL17-C18orf32	27.634688	13.21715	2.72E-14	1.068587	up
IFI30	17.159031	8.129418	7.05E-12	1.073396	up
AC131097.2	1.123846	0.480745	1.43E-10	1.197893	up

PAM16	1.958702	6.478565	2.95E-11	-1.76044	down
ZNF579	2.027725	4.88147	2.52E-13	-1.23752	down
LINGO3	3.216635	6.516422	3.02E-09	-1.01534	down
TRIM16	1.797595	0.180545	0	2.769212	up
HNRNPA1P48	0.310179	2.999654	0	-3.15377	down
NPIPA3	0.421169	6.669919	0	-3.47159	down
SAPCD1	9.551692	1.21345	0	2.914986	up
PPP5D1	1.273775	2.555782	9.48E-11	-1.60181	down
ANKRD63	0.526205	1.631866	0.000353	-1.54941	down
PET117	7.093263	3.260638	2.82E-08	1.110716	up
UQCRHL	14.159188	1.494782	0	3.226456	up
JRK	0.808137	7.906863	0	-4.67862	down
HNRNPUL2-BSCL2	3.309527	10.14884	0	-1.30137	down
RAMACL	1.940443	0.080178	0	4.324736	up
TMEM250	10.943756	4.729508	0	1.528994	up
TXNDC5	26.748909	61.37504	0	-1.20679	down
AMY2B	3.075937	1.437717	9.45E-09	1.297011	up
LY6G5B	12.657863	2.305163	0	2.135268	up
TMEFF1	0.507129	0.01565	2.43E-11	3.854478	up
PPAN-P2RY11	13.908003	0.681441	0	4.315751	up
KCTD7	3.695333	1.545477	0	1.749265	up
EFNA4	2.003867	4.734445	0	-1.54647	down
TICAM2	0.105491	1.409173	0	-3.5302	down
AL365232.1	1.313059	0.626988	2.77E-06	1.01243	up
P2RY11	1.095462	13.62396	0	-3.60566	down
C4A	0.065687	0.323224	4.06E-07	-2.1757	down
TNFSF12-TNFSF13	3.172327	1.5284	5.49E-05	1.027413	up
ZNF324B	3.242613	2.379864	1.43E-11	1.425264	up
RNF103-CHMP3	1.180878	0.472142	2.84E-14	1.53829	up
ARPC4-TTLL3	2.724986	6.132768	1.44E-11	-1.57014	down
CDK3	1.516861	3.116742	5.7E-05	-1.0466	down
TMED7-TICAM2	1.52944	0.409361	1.78E-15	1.854008	up
CENPS-CORT	0.899169	2.412963	6.73E-11	-1.77113	down
PCDHGB2	1.730665	1.001292	5.05E-07	1.148103	up
LRRC24	1.397525	5.187457	4.44E-16	-1.45056	down
ATP6V1G2-DDX39B	77.372819	35.02122	0	1.298421	up
STX16-NPEPL1	12.898298	2.768271	0	2.466575	up
AL133352.1	31.654062	9.315761	0	1.564417	up
AC135050.2	6.674866	2.695896	1.53E-07	1.008275	up

AP002990.1	22.534559	48.46406	0	-1.10511	down
AC011462.1	0.362352	0.777418	0.00057	-1.07406	down
TIFAB	0.182425	0.015434	3.35E-07	3.069928	up
ZNF10	0.705394	1.472614	0.002966	-1.0287	down
AC048338.1	10.829229	0.943072	0	3.483502	up
CNPY2	28.767458	6.62887	0	2.452125	up
MGAM2	0.201543	0.069643	0.002679	1.489085	up
TEN1	0.854941	14.73002	0	-4.65456	down
AL139011.2	2.680519	12.39593	0	-2.51832	down
AC068896.1	2.251458	7.417234	0	-2.52887	down
TRIM34	2.248574	6.28358	0	-1.47568	down
AL121594.1	2.724741	7.871139	8.88E-15	-1.17421	down
UBE2F-SCLY	4.686369	0.040063	0	7.25079	up
BLOC1S5-TXNDC5	49.914007	10.18022	0	2.689064	up
AC087632.1	1.963175	4.767195	2.69E-05	-1.19466	down
ST20-MTHFS	9.400036	4.528555	0	1.405626	up
AL136295.4	15.159755	5.870854	4E-15	1.066982	up
PMF1-BGLAP	8.435201	2.887085	7.55E-15	1.506773	up
AC026464.4	2.343021	0	0	9.360037	up
ISY1-RAB43	0.366837	0.131466	0.003867	1.431472	up
TMEM256-PLSCR3	7.710103	3.221152	0	1.658915	up
AC018512.1	2.445858	8.656769	0	-1.89601	down
AC135178.2	1.878432	0.451154	0	1.899402	up
AC134669.1	7.942193	2.078738	0	2.909197	up
AC074143.1	0.562696	2.03567	9.82E-07	-1.62799	down
NBPF15	2.480202	1.052521	1.11E-16	1.455715	up
AC067968.1	2.200263	0.559603	0	3.186287	up
AC093227.2	1.007841	4.31947	0	-2.19825	down
FDX2	0.367452	3.654107	0	-3.37864	down
LIN37	2.609652	1.022238	5.05E-09	1.739627	up
AC006486.1	0	8.001597	0	-9.26508	down
AC010463.1	16.224124	8.208089	4.44E-16	1.103104	up
AC003002.3	1.612321	0.703058	1.12E-12	1.191391	up
HIST2H4B	6.259376	2.933198	1.03E-11	1.243505	up
BORCS7-ASMT	0.375321	0	1.59E-10	4.978091	up
CD24	108.511551	45.65689	0	1.226253	up
AC104109.3	0.482333	45.44753	0	-6.27511	down
RBAK-RBAKDN	2.915171	4.891527	3.61E-10	-1.0473	down
AL359736.1	0.87475	1.829339	1.97E-08	-1.02155	down
TBC1D3K	0.905434	0	0	5.889013	up

SMIM11B	4.711387	0.652469	0	2.393353	up
SOCS7	2.793074	7.193755	0	-1.81759	down
HIST1H3B	0.508551	2.822906	0.000136	-2.21211	down
HIST1H3E	2.424445	0.553118	0	2.141844	up
F8A2	1.420618	5.241469	3.33E-16	-1.85489	down
LENG9	1.192673	2.750679	9.18E-07	-1.18326	down
FP565260.3	4.526891	2.936947	6.66E-16	1.156172	up
AC068946.1	7.897739	0.062778	0	6.143004	up
AC106886.5	0.414221	1.700652	0	-2.02517	down
AC068547.1	2.32511	2.379717	2.89E-12	1.490768	up
ZNF511-PRAP1	2.110578	8.37668	0	-1.96093	down
SCO2	0.082422	6.271338	0	-5.48574	down
AC004922.1	0.614579	15.83367	0	-3.26077	down
AC027644.4	3.558784	1.439046	0	1.33542	up
THSD8	0.100593	0.899122	4.52E-06	-3.25299	down
AC007240.1	33.094214	51.16535	0	-1.2836	down
DIABLO	2.736273	0.584025	0	2.179808	up
BX255925.3	0.232866	11.66692	0	-5.51234	down
AL451062.3	2.182379	15.12834	0	-2.73134	down
AL358113.1	0.965945	2.458759	0	-1.71537	down
AC006064.6	13.045521	32.44905	0	-1.22525	down
ABCF2	0.257509	0.813834	0.000352	-1.59161	down
AC012476.1	0.062425	4.973588	0	-6.0027	down
POLR2J3	0.902614	2.842619	0	-2.03158	down
SOD2	4.349273	0.206024	0	4.931369	up
AC097625.1	0.037273	1.204122	7.77E-16	-3.63012	down
AL031281.2	4.33536	2.617375	0	2.31382	up
AC010197.2	0.912231	0	0	6.724258	up
AC004687.2	0.437607	0.176394	0.000371	1.277642	up
AC087721.2	92.064033	65.07284	6E-15	1.00142	up
AC112128.1	2.597398	3.274241	1.06E-11	-1.07073	down
AC000120.3	0.219966	0.628959	0.00418	-1.43809	down
