# Supplementary material for: The snoRNA-like lncRNA LNC-SNO49AB drives leukemia by activating the RNA-editing enzyme ADAR1
Source: Cell Discov. 2022 Nov 1;8:117. doi: 10.1038/s41421-022-00460-9 (PMC9622897; doi:10.1038/s41421-022-00460-9)
Supplement: Supplementary file 12 — Supplemental Tab S5 [file 41421_2022_460_MOESM12_ESM.pdf]

Supplementary Table S5 List of the dysregulated genes of ADAR1 knockdown.

gene_name	si-ADAR1_FPKM	NC_FPKM	FDR	log2FC	regulated
ARX	0.348094	1.030269	1.59E-08	-1.5375	down
AGK	0.056695	1.929502	0	-4.99043	down
GOPC	9.352931	24.34056	0	-1.32861	down
EIF2AK2	6.284977	14.90035	0	-1.29894	down
ITIH4	0.080799	0.459631	1.74E-06	-2.17606	down
DRD4	0	0.263733	9.49E-12	-5.35096	down
AP3M2	3.6588	6.059662	0	-1.21135	down
VASH1	1.692678	3.846899	0	-1.24785	down
ACER3	1.926408	3.436938	0	-1.21708	down
TNPO1	22.234593	41.54869	0	-1.29182	down
SCAMP1	4.73711	13.13749	0	-1.57943	down
HIVEP1	1.524871	1.572564	0	-1.39695	down
SRPK1	18.938945	39.62571	0	-1.09961	down
NINL	0.857234	1.633325	1.19E-12	-1.14956	down
STK4	11.136436	20.66661	0	-1.70298	down
NDRG4	0.698633	0.953082	0.000708	-1.09541	down
MON1B	9.785736	15.20098	0	-2.23234	down
COTL1	11.557947	25.91677	0	-1.19247	down
ACSBG1	0.421306	0.268366	5.01E-13	-3.02222	down
CASP2	17.367806	40.22339	0	-1.36524	down
FSD1L	2.963196	2.166625	0	-1.34932	down
SHB	0.038428	0.348459	2.27E-06	-2.84488	down
TSPAN14	4.383942	9.319138	0	-1.45235	down
DCUN1D4	5.408232	8.460025	0	-1.28374	down
CORO1C	14.248628	30.41153	0	-1.08783	down
TBC1D30	0.787526	1.155775	1.25E-06	-1.18158	down
GOLT1B	3.460691	7.085781	0	-1.22436	down
PHACTR1	19.11062	14.61843	0	-1.00119	down
POLR3G	4.076714	8.620222	0	-1.46098	down
PECR	1.155175	2.658925	2.02E-11	-1.26884	down
TCEANC2	1.16652	2.497572	0	-1.71591	down
KMO	0.140182	0.40267	1.53E-05	-1.4871	down
SLC35A3	2.313864	3.542964	0	-2.32603	down
CLU	0.402336	1.307826	7.17E-05	-1.61235	down
SNRNP27	4.315493	16.67194	0	-2.17144	down
AL365205.1	5.393611	15.50529	0	-1.39682	down
RNF113A	0.121982	14.53369	0	-6.64856	down
ADORA2A	0.348846	0.865804	0.000411	-1.70246	down
CCDC62	0.176481	0.490181	0.000856	-1.39393	down
POLN	0.584241	2.335354	0	-1.60295	down
SLX1A	1.015671	3.113505	0	-2.21974	down
SCLY	7.893117	15.90936	0	-1.64566	down

BTG1	6.050405	19.70839	0	-1.57756	down
BIVM	0.185186	0.769608	4.05E-14	-2.19686	down
GLUL	31.894141	72.5129	0	-1.20156	down
ESR2	0.145337	0.558864	0.002303	-1.44927	down
CELF6	0.244527	1.021566	0.003758	-1.20784	down
TMEM91	0.572173	1.265772	4.35E-05	-1.78007	down
SIK1	0.241564	0.629176	6.63E-07	-1.35849	down
CCDC39	0.056104	0.658669	3.33E-06	-2.60753	down
TNFAIP8	6.901343	12.19901	0	-1.07516	down
AGBL3	0.169465	0.963926	3.27E-10	-2.06372	down
HMBOX1	2.997839	5.018676	0	-1.12256	down
NFIB	0.131027	0.374987	1.55E-05	-1.30953	down
TM7SF2	2.817102	8.350754	0	-1.68522	down
EEF1AKMT1	0.84694	5.953344	0	-2.8345	down
C12orf45	0.569867	4.320617	0	-2.39794	down
FER	1.221754	2.441211	0	-2.3754	down
FARP1	0.767952	1.543756	0	-1.74166	down
RABGEF1	8.084955	17.68829	0	-1.10085	down
APOOL	5.11123	8.603798	0	-1.45254	down
CABP1	0.770296	1.01903	1.12E-05	-1.32048	down
CDC42SE2	8.930345	22.07239	0	-1.36744	down
ARHGAP27	10.512117	15.42625	0	-1.28999	down
ADGRG5	0.304146	0.656974	0.002413	-1.07262	down
ZNF233	0.550968	1.077324	1.20E-08	-1.73757	down
HSF2BP	0.461974	1.145963	0.00104	-1.26661	down
ICOSLG	2.454796	4.468066	0	-1.996	down
C19orf47	6.918713	10.04739	0	-1.04982	down
ADAR	18.678395	51.08824	0	-1.43388	down
SENP3	9.232261	26.47749	0	-1.63652	down
NEU3	4.623501	10.68578	0	-1.26739	down
KRTCAP2	0.479763	18.27164	0	-5.11529	down
RHOBTB3	14.072836	28.81022	0	-1.34128	down
AMN	2.692304	5.55473	0	-1.02789	down
C16orf46	0.118653	0.58796	9.35E-05	-2.1438	down
ANKDD1A	0.592663	1.590719	0	-2.08901	down
PBX3	42.747876	61.3581	0	-1.04734	down
PRRT2	0.44818	2.971852	0	-3.04826	down
TUBA1A	50.029207	132.9824	0	-1.08557	down
RBPJ	29.514803	29.7698	0	-1.09923	down
CD2BP2	8.909511	19.62147	0	-1.18237	down
HSD17B13	0.072645	0.375642	0.001205	-2.1225	down
CLCN5	1.798689	2.599743	0	-1.0155	down
ZNF562	6.772819	8.511689	0	-1.53543	down
PRNP	10.982269	28.35112	0	-1.49022	down

RNASEH2C	10.703492	22.06765	0	-1.08029	down
AC097637.1	0	0.321488	4.88E-15	-5.64203	down
TLR10	2.188329	5.576119	0	-1.59816	down
AKIRIN1	10.327299	22.32635	0	-1.06997	down
SH3PXD2B	0.170798	0.387004	6.21E-10	-1.66727	down
ZDHHC14	0.440816	1.271895	7.06E-12	-1.38476	down
CHRNA7	1.542513	2.010383	0	-2.23898	down
MIEF2	2.680493	3.681212	0	-1.38321	down
MRFAP1	23.477666	51.94888	0	-1.05934	down
SLX1B	0.747229	14.49881	0	-4.83893	down
RFX7	2.911495	9.352482	0	-1.67528	down
DIABLO	9.124589	22.8448	0	-1.3514	down
PDIA2	0.044885	0.557308	1.89E-07	-3.27852	down
LMLN	1.104563	1.210955	1.04E-13	-1.49847	down
ZBTB3	1.139136	1.840635	8.05E-12	-1.48465	down
ZFP91	0.386148	34.69106	0	-6.48019	down
ZNF397	6.53942	11.65019	0	-1.07448	down
PPARA	2.169319	3.555556	0	-1.73006	down
ZNF546	0.617074	1.503354	0	-1.47156	down
PCDHB13	0	0.725389	0	-7.23963	down
CHP1	3.391108	12.0855	0	-2.13542	down
ZNF286A	3.166477	4.620699	0	-1.18414	down
LCN10	5.091319	17.27705	0	-1.95827	down
ZNF559	3.942248	7.142147	0	-1.26026	down
ZNF724	0.290483	4.144372	0	-5.06526	down
ZNF493	6.717923	11.17383	0	-1.47671	down
NTNG2	2.29014	2.246127	0	-1.65768	down
IPO4	7.57635	14.42467	0	-1.06876	down
ZNF785	2.373128	6.97166	0	-1.23133	down
CYSRT1	0.359052	1.302065	5.77E-06	-1.78532	down
ZNF98	0	0.906128	0	-8.13622	down
ZNF841	1.105108	2.802481	9.21E-15	-1.17314	down
MPEG1	0.171736	0.889457	0	-2.33722	down
SLC34A3	0.028373	0.520562	7.37E-10	-3.52548	down
POU3F3	0	2.189142	0	-8.10614	down
GP1BB	0.547541	1.542602	3.20E-06	-1.46542	down
AGER	4.100088	10.00527	0	-1.83213	down
CCDC85C	0.387968	1.90321	0	-2.11221	down
TSN	9.733885	37.56224	0	-1.88903	down
ZBTB9	2.513189	5.043448	9.99E-16	-1.05609	down
DDX47	0.719933	37.65773	0	-6.1001	down
IRF9	0.575473	4.767282	0	-3.65565	down
GANC	6.660295	10.32349	0	-1.48058	down
C17orf67	1.787227	2.965025	0	-1.48583	down

VPS16	5.384694	10.62411	0	-1.07117	down
SAPCD1	0.337044	1.21345	0.000524	-1.90133	down
PPP5D1	1.712966	2.555782	2.15E-10	-1.33504	down
RNF103	1.814361	5.328437	0	-1.63859	down
ACAD11	0.990861	4.019334	0	-3.94554	down
AMACR	1.546314	3.281401	0	-1.21867	down
CNTF	0.595833	1.481773	6.13E-06	-1.30619	down
EFNA4	2.176195	4.734445	0	-1.28065	down
ZNF487	0.886996	1.440114	3.48E-05	-1.73594	down
TRIM39-RPP21	2.388346	5.834081	0	-1.19309	down
RBM14-RBM4	9.073785	26.89786	0	-1.54357	down
TNFSF12-TNFSF13	0.359042	1.5284	5.23E-11	-2.0425	down
MSANTD3-TMEFF1	0.409434	1.128572	6.05E-06	-1.46786	down
NPIPB11	1.552958	2.336235	1.11E-16	-1.34136	down
LRRC24	1.137543	5.187457	0	-1.77409	down
CHMP4A	0.707455	4.103717	0	-2.4092	down
NPIPA2	0.322176	1.812228	0	-2.68219	down
AP002990.1	18.643202	48.46406	0	-1.38976	down
NPIPB8	1.499589	2.805525	0.001598	-1.13438	down
AC018523.2	1.006154	2.592855	3.16E-07	-1.35634	down
AP002748.4	0	1.629908	0	-7.89111	down
AC026786.1	1.840378	6.094713	0	-1.27269	down
TEN1	1.183752	14.73002	0	-3.92874	down
PDF	0.018694	10.96161	0	-7.66672	down
AL139011.2	5.427544	12.39593	0	-1.72583	down
TRIM34	2.195415	6.28358	0	-1.5233	down
PMF1-BGLAP	1.183243	2.887085	4.13E-10	-1.72805	down
AC090360.1	0.168148	1.031423	1.26E-05	-2.24472	down
UPK3BL1	9.50E-05	1.640964	0	-5.68228	down
FDX2	0.470792	3.654107	0	-3.17039	down
AC006486.1	1.173198	8.001597	0	-2.77144	down
AC104109.3	0	45.44753	0	-9.8113	down
SOCS7	2.969558	7.193755	0	-1.76017	down
HIST1H3B	0.506649	2.822906	4.40E-06	-2.35764	down
HIST1H2BH	0.277378	1.093547	2.69E-09	-1.93119	down
AC008073.3	0.011609	0.980954	2.98E-10	-4.08006	down
AC106886.5	0.719796	1.700652	0	-1.24785	down
ZNF511-PRAP1	3.853	8.37668	1.15E-12	-1.12145	down
AP001273.2	1.464445	4.117583	0	-1.07411	down

SCO2	0.999885	6.271338	0	-2.83579	down
AC004922.1	0.762654	15.83367	0	-3.25998	down
THSD8	0	0.899122	3.78E-11	-5.29109	down
AC007240.1	29.134567	51.16535	0	-1.48297	down
AL451062.3	4.984909	15.12834	0	-1.62787	down
AL358113.1	1.600692	2.458759	0	-1.94568	down
AC006064.6	10.503464	32.44905	0	-1.56565	down
POLR2J3	1.026653	2.842619	0	-1.51095	down
AC097625.1	0.132097	1.204122	4.96E-09	-2.00795	down
AC097634.4	1.368487	2.7967	1.17E-14	-1.04073	down
AC000120.3	0.045878	0.628959	2.34E-14	-3.41709	down
CASP10	3.189984	1.956379	1.11E-16	1.085037	up
ABCC8	0.480186	0.053815	0.002273	1.676714	up
NOS2	0.3282	0.098721	0.000909	1.634092	up
CDKL5	0.308715	0.218911	6.53E-10	1.791319	up
NME1-NME2	29.824673	0	0	10.44935	up
PLEKHB1	1.80983	1.113927	1.43E-08	1.485258	up
AKR7A2	39.913814	17.07089	0	1.227428	up
WNT8A	4.37965	0.363437	0	3.452442	up
BORCS8-MEF2B	1.684651	1.367325	0.001036	1.05325	up
KDM4A	14.904267	6.214681	0	1.45603	up
STON1-GTF2A1L	1.203309	0.020588	0	6.008362	up
DGCR2	14.60279	6.116689	0	1.258251	up
ZNF264	0.828308	0.01	0	5.749923	up
EFR3B	0.898778	0.73253	2.58E-14	1.357494	up
NID2	1.183532	0.629222	3.37E-06	1.11881	up
P2RX7	5.865546	2.936589	0	1.208587	up
PCBP4	16.27201	5.462898	0	1.576131	up
CAPN3	3.100657	1.221782	6.91E-05	1.126427	up
RASSF7	8.244247	3.413953	0	1.338162	up
UPB1	0.429059	0.178685	0.001326	1.61427	up
APOL1	3.516226	1.23886	0	1.519389	up
DHRS2	0.602209	0.023966	2.38E-08	3.57481	up
RASSF2	32.486564	9.125558	0	1.83556	up
RNMT	18.22646	8.911578	0	1.033501	up
TJP1	0.193731	0.0408	4.50E-06	2.060021	up
BLOC1S6	22.101499	7.597299	0	2.19874	up
LEPROTL1	17.531088	6.836068	0	1.534872	up
BNIP3L	10.837478	4.313267	0	1.505543	up
ZNF419	4.940435	2.369552	3.38E-12	1.05368	up
PIK3R2	2.58591	0.605009	0	2.348971	up
ERF	9.36231	5.03699	0	1.039696	up

RASA4	0.904562	0.252361	7.39E-11	1.816217	up
ZKSCAN1	18.188147	8.002505	0	1.215478	up
PCOLCE	4.195801	2.392986	8.66E-15	1.211379	up
PLEKHA1	0.356563	0.349803	8.25E-06	1.28957	up
CDH23	0.094605	0.028673	0.000193	2.36101	up
PTGES3L-AARSD1	5.673291	0.96327	0	3.267744	up
KCTD10	15.796735	6.217522	0	1.086117	up
NOP2	17.625187	1.0849	0	4.137426	up
GCNT2	0.545796	0.268441	0.008896	1.001462	up
NME5	1.347404	0.304959	1.48E-06	1.989043	up
CPEB4	1.174473	0.531305	0	1.536315	up
RNF146	7.838925	3.321872	0	1.651745	up
MED28	13.571566	4.715445	0	1.704601	up
MYL12B	128.709391	39.07579	0	1.742866	up
TJP2	3.607208	0.877325	0	1.698103	up
KIAA1217	0.229495	0.024214	1.09E-12	3.056632	up
GLT8D2	0.879871	0.164929	8.07E-05	2.06689	up
BHLHE41	0.421344	0.11839	3.94E-05	1.728458	up
TUBA4A	21.480311	7.144595	0	1.581298	up
MTERF1	8.015049	3.23056	0	1.401869	up
SPINK2	1.468985	0.332812	0.002607	1.924052	up
MGAT3	0.308716	0.128203	6.11E-15	3.911418	up
C22orf23	0.48233	0.003412	1.91E-08	4.302741	up
TMOD2	0.523051	0.234551	1.38E-07	1.127093	up
PGPEP1	2.54228	1.940986	7.92E-14	1.17456	up
AOC2	0.415466	0.001138	6.68E-12	4.659885	up
DCAF8	27.424847	18.2324	0	1.337738	up
RBM38	28.424124	12.63049	0	1.373979	up
STARD13	0.575052	0.766389	0.000145	1.215268	up
PRRG4	0.562524	0.232808	2.45E-05	1.200075	up
DNAJC14	2.137416	0.738989	1.09E-08	1.241154	up
STX6	15.798429	4.561343	0	1.778275	up
TMBIM1	10.795333	5.957475	0	1.241501	up
SRSF1	88.724909	35.10279	0	1.33235	up
THBS1	0.15743	0.030488	1.21E-05	2.21709	up
CENPO	15.08386	6.259261	0	1.176898	up
ETFBKMT	0.307191	0.27252	1.72E-05	1.959479	up
TMBIM6	125.799166	52.55252	0	1.305662	up
GNAL	0.44847	0.150991	0	3.620209	up
IGFBP4	0.639248	0.211617	0.001691	1.548521	up
DNMT3L	1.655359	0.037689	0	5.216744	up
AC020909.1	3.796386	0.269485	0	3.624514	up
GABPB2	2.640337	1.712098	0	1.560407	up

SOX13	0.599941	0.381967	0.001794	1.187127	up
PTPN7	25.556447	13.09415	0	1.136328	up
CTDSPL	1.877153	0.548207	0	2.182355	up
ADPRH	0.648431	0.500542	2.31E-06	1.450686	up
ARMT1	13.531306	5.723391	0	1.26598	up
ZC3HAV1L	21.75171	10.49164	0	1.1663	up
NLGN4X	0.404386	0.169714	0.000504	1.202365	up
ZNF182	2.623671	0.566772	0	2.152133	up
SURF4	35.819826	12.47439	0	1.536287	up
TMEM25	1.246341	0.33981	2.56E-05	1.533513	up
BCL2L11	8.638917	3.10278	0	2.150606	up
DGKE	19.603089	10.12501	0	1.377752	up
PIEZO2	0.278939	0.066219	1.48E-11	2.119143	up
CLDN12	6.052698	2.811181	0	1.009252	up
MYO1E	0.335331	0.104929	3.89E-09	1.641608	up
FAM86C1	4.978507	1.869015	0	1.76594	up
IER2	16.682584	1.700108	0	3.332745	up
COL6A3	1.349907	0.27472	0	1.764424	up
EIF4E3	0.186046	0.077872	0.006823	1.201089	up
UVSSA	4.259336	3.181375	0	1.649667	up
BAALC	0.560407	0.104211	9.78E-05	2.240007	up
ABCA1	1.385922	0.555649	0	1.195717	up
NDST2	5.499926	1.037646	0	1.963042	up
TPM4	247.067816	122.6826	0	1.045412	up
SHOX2	0.305976	0.076767	0.009368	1.840446	up
ZNF608	4.058349	2.061168	0	1.011487	up
TMEM129	12.233765	4.120621	0	1.79389	up
ZBTB43	2.691679	0.70583	0	2.31375	up
CCDC126	1.242205	0.869669	5.11E-05	1.189624	up
RRM2	41.42025	12.39038	0	1.855889	up
SMN1	28.543231	12.23764	0	1.310462	up
ZNF24	25.536523	4.904228	0	2.422612	up
VANGL1	0.638273	0.477134	5.05E-10	1.240054	up
SPDYE2B	1.294602	0	0	8.925507	up
RGMB	1.041874	0.791741	5.96E-08	1.266577	up
IQCK	1.413868	0.522097	2.40E-09	1.905299	up
PDIK1L	8.841368	5.266018	0	1.15255	up
CENPS	11.881801	5.886257	5.29E-12	1.006665	up
ORAI3	13.638809	4.137488	0	2.294715	up
CDK5R1	3.416229	1.057412	0	1.671076	up
GPR150	1.287214	0.061608	0	4.007131	up
APOLD1	28.611691	9.630404	0	1.405685	up
CXCR2	0.702173	0.325797	3.41E-06	2.218251	up
CLN8	1.085651	1.527288	2.92E-09	1.013127	up

BRICD5	5.681866	3.124146	0	1.329835	up
CAMK1D	12.107059	5.862733	0	2.040176	up
GPR19	1.405595	0.56941	0.009122	1.131244	up
VPS33B	2.338783	1.033671	4.27E-09	1.15291	up
CCR10	2.180846	0.800903	1.84E-08	1.313334	up
TMEM186	4.025424	0.614235	0	2.662396	up
TMEM121	2.9068	1.499395	3.72E-09	1.149355	up
ZNF749	5.563502	1.595386	0	1.998389	up
VMAC	2.876799	0.063846	0	5.111126	up
ZNF383	1.831102	0.990664	0	1.313409	up
ZNF177	1.923065	0.847977	1.06E-10	1.588292	up
ZNF569	4.077268	2.677316	7.77E-16	1.015276	up
HSH2D	14.99891	5.741136	0	1.716946	up
ZNF700	8.630677	5.888148	0	1.303711	up
KIAA1671	0.162116	0.019451	0.000272	2.116684	up
RAB40C	9.519945	4.209561	0	1.622263	up
STKLD1	0.66065	0.130402	0.004624	1.603486	up
CIPC	7.907761	1.797994	0	2.147155	up
LCN8	16.630138	6.438585	0	1.38093	up
OXLD1	6.876882	2.296714	1.25E-11	1.380323	up
PBX2	32.269391	8.349042	0	1.693049	up
MSH5	3.799795	1.479303	0	1.571124	up
ZNF814	2.666031	1.859447	0	1.378834	up
TRIM39	3.544024	1.444277	0	1.525271	up
ZNF468	16.298857	7.825011	0	1.034051	up
SYCE1L	5.629451	1.914689	0	1.690803	up
SPDYE2	2.648906	1.483762	0	2.924625	up
LEPROT	6.033731	1.97571	0	1.787583	up
LBH	149.301832	52.30173	0	1.532171	up
TOMM6	94.462662	0.000409	0	9.744324	up
CEBPZOS	30.909268	15.85295	0	1.306005	up
TRIM16	1.338433	0.180545	0	2.663648	up
HNRNPA1P48	9.853376	2.999654	0	1.69507	up
RAMACL	0.706967	0.080178	5.94E-10	2.943275	up
TMEM250	12.454178	4.729508	0	1.634031	up
LY6G5B	12.748924	2.305163	0	2.151792	up
EGFL8	2.631583	0.134704	0	4.431221	up
TMEFF1	0.678392	0.01565	0	4.398146	up
PPAN-P2RY11	4.708858	0.681441	0	2.752073	up
DNAJC25-GNG10	10.995256	0	0	9.337485	up
TMEM110-MUSTN1	0.623462	0	5.78E-10	5.204981	up

ZNF324B	4.255129	2.379864	0	1.915898	up
RNF103-CHMP3	3.085432	0.472142	0	3.246367	up
GLI4	2.899199	1.420424	0.000114	1.030919	up
AL136295.1	20.052305	8.187458	0	1.391244	up
AP002884.3	4.266614	0.304293	0	4.071297	up
EID1	97.279839	44.18369	0	1.126497	up
AC010132.3	11.362998	0.022763	0	7.308519	up
AC048338.1	10.489385	0.943072	0	3.434067	up
LIMS3	1.527133	0	0	6.633649	up
GATC	14.977929	4.737413	0	2.068298	up
CNPY2	28.423672	6.62887	0	2.532695	up
SPECC1L-ADORA2A	0.70077	0	0	7.470105	up
BCL2L2-PABPN1	2.149856	0	0	6.91119	up
UBE2F-SCLY	6.576475	0.040063	0	7.878916	up
AL136295.4	13.894801	5.870854	0	1.021129	up
AC138811.2	15.100805	5.351694	0	1.476663	up
PECAM1	0.673677	0.57228	0.002038	1.102092	up
BOLA2-SMG1P6	6.888875	3.392667	1.55E-15	1.214051	up
ISY1-RAB43	2.518447	0.131466	0	4.17791	up
AC119396.1	0.917533	0.28441	0.000592	1.628427	up
AC135178.2	0.991568	0.451154	1.76E-05	1.119508	up
AC005943.1	4.035908	0.552334	0	2.773224	up
AC004223.3	1.104548	0	0	7.632051	up
AC024592.3	1.713073	0.76798	1.22E-05	1.867689	up
LIN37	2.241769	1.022238	2.17E-05	1.373448	up
CCL5	3.951856	1.933572	3.15E-07	1.039029	up
CD24	120.013302	45.65689	0	1.238225	up
AL713999.1	25.838641	8.260551	0	1.401804	up
TBC1D3K	1.036705	0	0	6.490788	up
SMIM11B	5.215665	0.652469	0	2.519955	up
FP565260.3	4.8052	2.936947	0	1.304854	up
F8A3	2.552675	0.937926	8.89E-11	1.411199	up
SENP3-EIF4A1	49.487232	21.86174	0	1.166328	up
VSIG10L2	0.467803	0.126362	1.68E-07	1.732042	up
AC027644.4	3.851234	1.439046	0	1.391992	up
DIABLO	4.352926	0.584025	0	2.841846	up
AC104472.3	6.755476	3.262425	0	1.03823	up
ARHGAP11B	3.897249	0.000789	0	6.458839	up
Z83844.3	1.276443	0	0	7.351069	up

AC117378.1	4.301919	0.363463	0	3.360423	up
AL031281.2	7.157319	2.617375	0	2.627852	up
AP000944.2	5.994725	1.465215	0	1.965021	up
AC010197.2	0.442894	0	0	6.105811	up
AC008012.1	2.059206	0.881575	0	1.206938	up
AC087721.2	93.25721	65.07284	0	1.00856	up
