# Supplementary material for: The snoRNA-like lncRNA LNC-SNO49AB drives leukemia by activating the RNA-editing enzyme ADAR1
Source: Cell Discov. 2022 Nov 1;8:117. doi: 10.1038/s41421-022-00460-9 (PMC9622897; doi:10.1038/s41421-022-00460-9)
Supplement: Supplementary file 13 — Supplemental Tab S6 [file 41421_2022_460_MOESM13_ESM.pdf]

Supplementary Table S6 List of primers, siRNAs and antibodies in this study.

Primer	Sequence (5'-3')
LNC-SNO49AB-F	TCAGCCACAGTCCTAACCTA
LNC-SNO49AB-R	CAGTCAAGCACCTCACAACC
SNHG29-F	TGAGTATTGGAGCCAGGAGC
SNHG29-R	CTGCTGCCAGCTCTAAAACA
Pre-SNHG29-F	ACAGTGTAGCCTATCCCAGAG
Pre-SNHG29-R	CCGAGACTAAACAGCCAAGAG
SNORD49B-RT	GTCGTATCCAGTGCAGGGTCCGAGGTATTCGCACT GGATACGACTCGTCAG
SNORD49B-F	CTGATGATACTTGTAATAGGAAG
SNORD49A-RT	GTCGTATCCAGTGCAGGGTCCGAGGTATTCGCACT GGATACGACAATCAGAC
SNORD49A-F	TGCTCTGATGAAATCACTAATAG
SNORNA -R	GTGCAGGGTCCGAGGT
LNC-SNO49AB -FL-F	CTTTTCATTC CCGTTGTTAT GG
LNC-SNO49AB -FL-R	AATCAGACAGGAGTAGTCTTCG
LNC-SNO49AB -5race –N-R-outer	GTTATCGCTTCTGACGGCACTT
LNC-SNO49AB -5race –N-R-inner	GTCAAGCACCTCACAACCCTCC
LNC-SNO49AB -3race –N-R-outer	AATAGGAAGTGCCGTCAGAAGC
LNC-SNO49AB -3race –N-R-inner	GAAGTGCCGTCAGAAGCGATAA
ADAR1-q-F	CTGAGACCAAAAGAAACGCAGA
ADAR1-q-R	GCCATTGTAATGAACAGGTGGTT
TRSA-F	TAATACGACTCACTATAGGGAAAAAAAA
TRSA-R	GAATTCTTTTTTTTTTTCTGCAGTGG
ADAR1-1065 -R	ATAAGAATGCGGCCGC CTACAAATGCCATATGGGAGGGG
ADAR1-1506 -R	ATAAGAATGCGGCCGC CTACTTCAGCTGGCACTCTGTCAG
ADAR1-2529 -R	ATAAGAATGCGGCCGC CTACTGGTCATGGAAGGTGCTGCC
ADAR1-FL-R-NOT1	ATAAGAATGCGGCCGC CTATACTGGGCAGAGATAAAAGTTC
ADAR1-502aa-F-bstbI	CCGTTCGAA AAGAACCCCATCAGCGGGCTGTTAG
ADAR1-502aa-F-N-HA	CCGTTCGAAATGTACCCTTATGACGTACCTGACTA TGCTGGAAAGAACCCCATCAGCGG
ADAR1-843aa-F-bstbI	CCGTTCGAA CAGATAGCCATGCTGAGCCACCGG
ADAR1-RBD-R-NOT1	ATAAGAATGCGGCCGC TACCTCTGTGAAACCCATGCGTTCTGCC

LRRC75A-qF	TATCGCTACGCCAGCTTCC	
LRRC75A-qR	TGGGACAGTGGATGTAGCGT	
**siRNA/smart silencer**	**Sequence (5'-3')**	
si-ADAR1-1	CCACUAUUCCACAGAGAAATT	
si-ADAR1-2	CCAUGAACCCAAGUUCCAATT	
si-LNC-SNO49AB	CCAGUAGCAGGGUUAGCUUTT	
smart silencer	GCATAGGGTGGAACTTAAAT	
	CTCCTTCGTTAAGAAATGTC	
	GAAACAAACTCCTTCGTTAA	
	TCTCTGGTGTAGGGTTTAA	
	GTAACGTTATCATGTCATT	
	TAACCTAGAACTGCATTTA	
**Antibody**	**Company**	**Catalog number**
ADAR1	Abcam	ab88574
FLAG	Sigma	F1804-1MG
HA	CST	3724S
FBL	Abcam	Ab5821
NOP58	Proteintech	14409-1-AP
NOP56	Proteintech	18181-1-AP
15.5K	Proteintech	15802-1-AP
